# Using positive imagination to reduce negativity in information processing and hesitant attitudes towards childhood COVID‐19 vaccinations in parents: A randomized controlled trial

**DOI:** 10.1111/bjhp.12808

**Published:** 2025-06-03

**Authors:** Jiehu Yuan, Meihong Dong, Wendy Wing Tak Lam, Qiuyan Liao

**Affiliations:** ^1^ School of Public Health, Li Ka Shing Faculty of Medicine The University of Hong Kong Hong Kong China; ^2^ Hospital‐Acquired Infection Control Department Affiliated Foshan Hospital of Southern Medical University Foshan Guangdong China

**Keywords:** childhood vaccination, information processing, positive imagination, psychological distress, vaccine‐hesitant attitudes

## Abstract

**Objectives:**

We aim to investigate the impact of negative information processing on parental vaccine hesitancy in Hong Kong and design an intervention to reduce negativity in pandemic and vaccine‐related information processing.

**Design:**

Six hundred and forty‐seven parents were recruited for baseline assessment. One week later, participants were randomly assigned to either the positive imagination simulation (PIS) intervention group or the neutral recall simulation (NRS) control group. Participants completed outcome assessments immediately and 2 weeks after the intervention.

**Methods:**

We first examined whether affective response to pandemic and vaccine‐related news mediated the association between parents' distress and acceptance of childhood COVID‐19 vaccination using baseline data. The PIS intervention leveraged positive psychology and personalized imagery techniques to enhance positive affect. To test intervention effectiveness, ANCOVAs were conducted to examine whether PIS versus NRS could reduce negative affective response to pandemic and vaccine‐related news (immediate effect) and COVID‐19 vaccine‐hesitant attitudes (effect at the 2‐week post‐intervention point).

**Results:**

The baseline assessment showed that greater distress was linked to a more negative affective response to pandemic and vaccine‐related news, which was associated with lower acceptance for childhood COVID‐19 vaccination. The intervention positively impacted valence rating (*F*(1, 627) = 8.46, *p* = .004) and affective state rating (*F*(1, 627) = 4.88, *p* = .028) on pandemic and vaccine‐related news. This improved positivity spilled over to significantly enhance parents' trust in COVID‐19 vaccine‐related information and alleviate their vaccine safety concerns 2 weeks post‐intervention.

**Conclusions:**

Our study highlights the promising impact of positive affect priming in increasing positivity in information processing and, consequently, reducing vaccine‐hesitant attitudes that are modifiable through positive information processing.

**Statement of contribution**

**What is already known on this subject?**

- Psychological distress can cause a negativity bias in information processing through cognitive mechanisms of attention, interpretation and memory.
- Current vaccination communication primarily targets cognitive processes, rendering it less effective in addressing distress‐induced vaccine hesitancy.
- Interventions based on positive imagination held potential for increasing positivity and motivating people to engage in various health behaviours.

**What does this study add?**

- Distress‐induced negative information processing biased parental vaccination decision.
- A novel positive imagination intervention fosters positivity during subsequent information processing.
- The positive affect priming increased information trust and reduced parental vaccine safety concerns 2 weeks after the intervention.

## INTRODUCTION

The COVID‐19 vaccine rollout initially targeted adults, but later expanded to cover children aged 6 months to 17 years in response to the escalating COVID‐19 infection rates among this age group (World Health Organization, 2022). While most COVID‐19 cases in children are either asymptomatic or mild, 18.4 per 100,000 children aged 0–4 and 10.6 per 100,000 children aged 5–17 require hospitalization due to COVID‐19 infection (Centers for Disease Control and Prevention, 2023). Additionally, COVID‐19 increases the risk of severe multisystem inflammatory syndrome (MIS‐C) among previously healthy children (Kamidani et al., 2020). COVID‐19 vaccination is not only effective in protecting children against COVID‐19 but is also a crucial strategy to minimize school interruptions in children (Kamidani et al., 2020). However, parental vaccine hesitancy represents a significant obstacle to children's vaccination uptake, with over 30% of parents expressing COVID‐19 vaccine hesitancy globally (Lazarus et al., 2022).

### Psychological distress as a potential contributor to vaccine hesitancy

Existing reviews highlight factors contributing to parental COVID‐19 vaccine hesitancy, predominantly focusing on vaccine‐specific factors such as parents' confidence in vaccine safety and effectiveness, as well as perceived low severity of the disease (Bianchi et al., 2023; Galanis et al., 2022; Khan et al., 2022). However, parents may not necessarily follow the rational course of cost‐and‐benefit analysis to make their vaccination decisions for children, especially in the pandemic context when the media is saturated with negative news and misinformation (Lockyer et al., 2021; Yuan, Dong, et al., 2023; Yuan, Lam, et al., 2023). Recent studies identified a negative correlation between parental mental distress and childhood COVID‐19 vaccination acceptance among both healthy parents (Evans et al., 2021; Xu et al., 2021) and those with a diagnostic history of one or more mental health conditions (Milan & Dáu, 2021). However, these studies only reported the correlations between parental distress and COVID‐19 vaccination acceptance, leaving the underlying mechanisms unexplored.

A recent study found that psychological distress was associated with more negative processing of vaccine‐related information, leading to higher vaccine hesitancy (Yuan, Dong, et al., 2023; Yuan, Lam, et al., 2023). Previous studies have also suggested that psychological distress affects people's information processing through cognitive mechanisms of attention, interpretation and memory (Bomyea et al., 2016). First, cumulative stressors prompt the use of affective heuristics as a cognitive‐saving strategy (Finucane et al., 2000), resulting in biased information processing where parents selectively focus on the negative aspects of the vaccine (Albery et al., 2021; Yan et al., 2020) and establish a negative affective association (Klasko‐Foster et al., 2020; Yuan, Dong, et al., 2023; Yuan, Lam, et al., 2023). Second, persistent distress primes negative interpretation of ambiguous cues (Elwood et al., 2007; Nortje et al., 2004; White et al., 2008), leading to overestimation of vaccine risks and thereby potential refusal of childhood vaccination (Evans et al., 2021). Third, distress enhances recall of negative emotional memories (Luethi, 2008) while impairing recall of neutral information (Gagnon & Wagner, 2016), further reinforcing parents' negativity bias by making negative vaccine‐related events more salient in their implicit memory (Azarpanah et al., 2021). Taken together, psychological distress can alter information processing to be more affect‐driven, resulting in biased vaccination decisions. Existing studies on COVID‐19 vaccine hesitancy emphasized more on objective attributes of information, such as information sources and adequacy (Alfieri et al., 2021; Ruggiero et al., 2021; Temsah et al., 2021), while neglecting the role of individuals' information processing in shaping their vaccination decisions.

### Risk communication strategies to address distressed parents' vaccine hesitancy

Based on one recent review, studies have implemented various risk communication strategies to enhance parental confidence in childhood COVID‐19 vaccination, but informational interventions have shown limited effectiveness (Huang et al., 2023). One widely employed messaging technique for promoting children's vaccination is the use of either gain‐framed or loss‐framed messages (Penţa & Băban, 2017; Wang et al., 2022). These strategies primarily influence cognitive processes by manipulating individuals' perceived costs and benefits of taking vaccination (Penţa & Băban, 2017). However, they may not adequately consider biases in information evaluation resulting from psychological distress (Albery et al., 2021). Additionally, loss‐framed messages may even amplify negative mood and lead to psychological reactance and a preference for the status quo (Zhao & Nan, 2010). For parents distressed by the pandemic itself and related containment measures, it is important to address their biases in information processing by enhancing their affective state (Argyris et al., 2021; Rapisarda et al., 2021). Positive imagination tasks, involving vivid and personalized mental simulation (Daniel et al., 2016), have consistently demonstrated effectiveness in eliciting positive emotions (Murphy et al., 2015), reducing cognitive biases (Holmes et al., 2009) and motivating people to engage in various health behaviours, ranging from healthier eating habits (Knäuper et al., 2011) to increased engagement in preventive behaviours against the pandemic (Sinclair et al., 2021). Overall, employing emotionally charged and personalized imagination techniques has the potential to evoke greater positive affect in parents and reduce negative information processing, potentially alleviating vaccine hesitancy.

### The current study

During the COVID‐19 pandemic, parental distress in Hong Kong surged, with 59.2% reporting elevated stress levels (Wong et al., 2022). On the contrary, despite vaccines being widely available and free of charge for all children aged 3–11 years since March 2022, the COVID‐19 vaccination rate in this age group remained stagnant, staying below 60% during our study periods (Figure S2). This rate was significantly lower than the 69.5% COVID‐19 vaccination acceptance for children reported by parents in other countries during the same period (Lazarus et al., 2023). Additionally, since no COVID‐19 vaccine mandate was in place for children aged 5–11 at the time of the study (Centre for Health Protection, 2023), the vaccination decision for children was entirely dependent on parents. Within this context, this study examines the association between parental psychological distress and affective response to pandemic and vaccine‐related news and acceptance of children's COVID‐19 vaccination. We aimed to test the effect of positive imagination on reducing parents' negative affective evaluation of pandemic and vaccine‐related information and their concern about vaccine safety. For the first aim, we hypothesized that greater psychological distress would lead to a more negative affective response to pandemic and vaccine‐related news (H1a), which in turn was associated with lower parental acceptance of children's COVID‐19 vaccination (H1b). Upon confirming these associations, we proceeded to test a positive imagination simulation (PIS) intervention designed to induce positivity during information processing. We hypothesized that this intervention would be more effective than a neutral recall simulation (NRS) control group in enhancing parental positive affective response to pandemic and vaccine‐related news (H2a), particularly for parents with higher distress levels (H2b). Based on the associations identified in H1a and H1b, we anticipated that increasing positivity could have a positive effect on subsequent vaccination attitudes. Therefore, as a non‐preregistered exploratory analysis, we hypothesized that the positive impact of our intervention would have an impact on parental vaccine‐hesitant attitudes towards childhood COVID‐19 vaccinations. While the PIS intervention might not change overall vaccine hesitancy or deeply rooted values, it could potentially influence attitudes that are modifiable by positive information processing. Specifically, we hypothesized that our intervention might have a spillover effect by increasing parental trust in COVID‐19 vaccine information and reducing concerns about the vaccine safety after 2 weeks (H2c). These two aspects are relevant to our intervention because inducing positive affect during information processing could lead parents to pay more attention to positive information about COVID‐19 vaccines and resist the anti‐COVID‐19 vaccine arguments, thereby increasing trust and reducing safety concerns. Furthermore, the improvement in vaccine‐hesitant attitudes within the PIS group would be mediated by an increase in positive affect (H2d).

## METHOD

### Participants

Eligible participants were adult parents with at least one young child eligible for COVID‐19 vaccination during the COVID‐19 pandemic in Hong Kong. Additional criteria included being able to read and comprehend Chinese and confirming no medical contraindications for vaccination in their children. Participants were primarily recruited from a panel of parents who had previously participated in our study on parental attitudes towards a school‐based seasonal influenza vaccination programme in Hong Kong (Dong et al., 2023). The panel involved 1390 parents with at least one young child attending kindergarten or primary school, recruited through random‐digital‐dialled telephone interviews. To account for potential attrition, participants were encouraged to invite eligible friends to join. We preregistered our sample size as 600, determined to achieve 80% power to detect the expected intervention effect of 0.26 using G*Power 3.1.9.7 (Faul et al., 2007). The expected effect size was based on the post‐test outcomes reported in a meta‐analysis of positive psychology interventions (Bolier et al., 2013). Since our study focuses on affective reactions, we selected a similar non‐clinical outcome, affective appraisal, from this meta‐analysis to estimate the effect size. The final sample comprised 87% (564/647) of participants recruited from our representative panel (random sample) and 13% (83/647) referred by friends (convenience sample). We examined the differences between the two samples obtained using different recruitment methods and found no significant differences in participants' demographics and major characteristics measured at baseline. The only exception was that the random sample had a higher COVID‐19 vaccination acceptance rate compared to the convenience sample (*p* = .02) (Table S1). This indicates that our recruitment method did not significantly impact the main results related to participants' information processing. This study was approved by the Institutional Review Board of the University of Hong Kong/Hospital Authority Hong Kong West Cluster (Reference No.: UW 22‐102).

### Procedures

**FIGURE 1:**
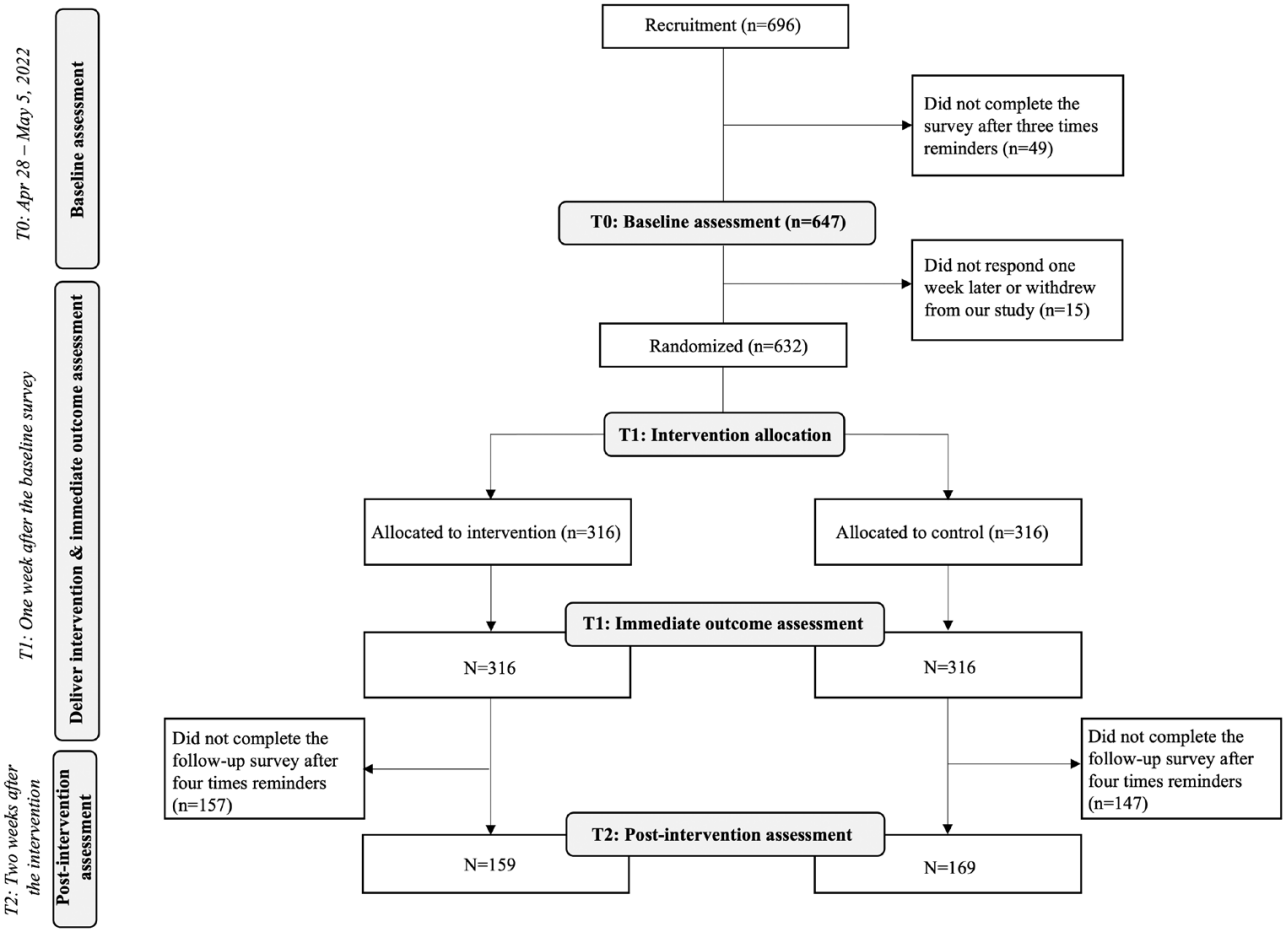
Number of potential eligible parents who consented to participate at the three study phases.

This study was preregistered on ClinicalTrials.gov (Ref No.: NCT05298865) and involved a baseline assessment phase (T0), an intervention and immediate outcome assessment phase (T1) and a post‐intervention assessment phase (T2) (Figure 1).

In April 2022, we initiated the baseline assessment (T0) by sending invitation messages to eligible participants who had previously provided oral consent to be re‐contacted for health‐related study via their mobile phones. The message introduced the study's purpose and outlined the procedures for completing two major surveys at consecutive time points. Each participant received a unique participant code to link their assessment data. To proceed with the baseline assessment, participants were required to read the consent information on the survey's cover page to indicate their consent and input their participant codes. To improve the response rate, we sent four additional message reminders to those who had not completed the survey. These reminders were delivered at different timeslots over four consecutive days, covering both working and non‐working hours. Upon completion of the survey, the participants were thanked, and they were reminded that another survey would be delivered to their mobile phones 1 week later.

One week after completing the baseline survey, participants were invited to complete a follow‐up survey. The study's purpose was generally stated as: ‘*This study aims to understand your associative abilities and your views on information related to the COVID‐19 pandemic*’. Participants were then randomized into either a control group or an intervention group. Randomization was achieved using the randomizer function in the Qualtrics platform to ensure equal allocation. As an incentive, participants who completed both the T0 and T1 surveys received a supermarket coupon valued at approximately USD 6. To gauge the spillover impact of our intervention, participants were re‐contacted for a follow‐up outcome assessment 2 weeks after the intervention (T2). The consent and reminder procedures for the T1 and T2 surveys mirrored those used in the T0 survey.

### Measurement

#### Baseline assessment at T0

##### Psychological distress

We assessed participants' probable anxiety and depression using the Patient Health Questionnaire (PHQ‐4), a brief 4‐item measurement (Kroenke et al., 2009). Participants indicated the frequency of experiencing the situations in the past 2 weeks related to worrying, feeling down or hopeless, lack of interest or pleasure, and feeling nervous or anxious. They responded on a 4‐point Likert scale, ranging from 0 (Not at all) to 3 (Nearly every day).

##### Affective response to pandemic and vaccine‐related news

We used six pre‐tested real‐world news articles, randomly assigned to either the pre‐ (T0) or post‐stimulus (T1) sections, to assess participants' affective response to pandemic and vaccine‐related news. The news covered topics including the societal impact of the pandemic, current pandemic situation and COVID‐19 vaccines and treatments for children. Hereafter, we refer to all the news used in our study as pandemic and vaccine‐related news. The news items were adapted to ensure balance in word count and structure. Each news item was presented in two paragraphs on a single screen page, with the upper paragraph containing risk‐related information (e.g., describing the mortality rate of the pandemic), and the bottom paragraph featuring solution‐related information (e.g., discussing how vaccination can mitigate the risk). This content structure was aimed at balancing the news's affective valence. We pretested the news items to ensure that they were neutral in affective valence and easy to understand (File S1). Participants rated their valence evaluation (‘How positive or negative do you think the news is?’) and affective state (‘How optimistic or pessimistic do you feel after reading the news?’) on a bipolar scale ranging from −3 to 3. Participants were presented with three news items in the T0 and T1 surveys, respectively, with the order randomized.

###### Vaccination acceptance

We assessed children's vaccination acceptance by inquiring parents about their children's COVID‐19 vaccine uptake status. Those who answered ‘No’ were subsequently asked: ‘How likely will you take your child for COVID‐19 vaccination in the next 3 months?’ Participants rated their intention on a 7‐point Likert scale (definitely will not, very unlikely, unlikely, even, likely, very likely or definitely will). If participants had more than one child, they answered based on their youngest child eligibility for COVID‐19 vaccination. Those who expressed a positive intention or reported that their child had already received the vaccine were grouped to indicate higher acceptance of COVID‐19 vaccines, while others were classified as having low vaccination acceptance. This approach to assessing vaccination acceptance is consistent with methods used in recent COVID‐19 vaccination research (Lazarus et al., 2023; Xiao et al., 2022; Yuan, Dong, et al., 2023; Yuan, Lam, et al., 2023).

##### Vaccine‐hesitant attitudes

We used a five‐item short scale adapted from the Parent Attitude about Childhood Vaccines Instrument (PACV) to measure parental vaccine‐hesitant attitudes (Opel, 2014). This scale reliably predicts children's up‐to‐date vaccination status for all recommended childhood vaccines (Oladejo et al., 2016), and has been applied to studies on parental COVID‐19 vaccine hesitancy recently (Bianco et al., 2022; Higgins et al., 2023). For the current study, we used a modified version of PACV short scale to better fit the context of Hong Kong while capturing distinct attitudinal aspects of parental vaccine hesitancy. This short scale was pretested with ~10 parents to ensure its acceptability and comprehensibility. Specifically, participants responded on a 5‐point agreement scale with five statements regarding their trust in vaccine‐related information (e.g., ‘I trust the information I receive about COVID‐19 vaccines’), preference for building immunity through infection but not vaccinations (e.g., ‘I favor my child to get immunity from natural methods over that from COVID‐19 vaccination’), preference for fewer vaccinations at once, concerns about COVID‐19 vaccine safety and overall COVID‐19 vaccine hesitancy. The reliability check demonstrated a satisfactory level (Cronbach's *α* = .80). The complete measurements are presented in Table S2.

##### Demographics

Participants' demographics, including parental age, gender, educational attainment, employment status, marital status and monthly household income, were recorded at the end of the baseline assessment.

#### Intervention and immediate outcome assessment at T1

##### Manipulation

Participants assigned to the PIS group were informed that they would engage in interactive tasks designed to assess their imagination skills. The simulation involved envisioning three life scenarios using pictures that matched their self‐reported gender. This approach aimed to enhance the personal relevance and engagement with the tasks (Schubert et al., 2020). Participants received step‐by‐step instructions, starting with imagining their best friend's name, personality and voice, before delving into more substantial details about scenario imagination. The scenario design incorporated components from positive psychology interventions (Bolier et al., 2013; Lyubomirsky, 2007), including the PERMA model, which encompasses positive emotions, engagement, relationships, meaning and accomplishments (Seligman, 2018), and the Synergetic Change Model, which includes emotions, goals and habits, virtues and relationships, comprehension and coping, and attention and awareness (Rusk et al., 2018). Table 1 provides details of our scenario examples and corresponding strategies based on positive psychology theories (Rusk et al., 2018; Seligman, 2018). To ensure that participants had sufficient time to complete the imagination tasks, they were instructed to pause for at least 5 s before proceeding to the next page using the timing function in Qualtrics. In the NRS control condition, participants were instructed that the task was aimed at testing their recall capacity. Specifically, participants were instructed to recall neutral objects from their daily lives. Both groups had equal amounts of time for completing the tasks. We aimed to enhance the positive affective state in the PIS group. The full materials for the PIS and NRS are available in File S2.

**TABLE 1:** Scenario, imagination instructions and corresponding theoretical components drawn from positive psychology in the positive imagination simulation intervention group.

Scenario	Instruction	Theoretical components in positive psychology
Spending time with best friend	Please imagine you are having afternoon tea with your best friends at the restaurant.	Investing in social connections Savouring the moment
	Please imagine you are sharing recent good news with your friends, imagine how you feel about it.	Investing in social connections Thinking about positive life experiences
Travelling with family	Please imagine you are experiencing new things together with your family during the journey.	Positive future forecasting Investing in social connections
	Please imagine during the journey, you are gratitude that your family are being with you at this moment.	Savouring the moment Practicing gratitude
Writing gratitude letter with child	Please imagine you are writing a gratitude letter with your child after school, imagine how you feel when your child is being close to you.	Savouring the moment Investing in social connections
	Please imagine you are sharing your gratitude letter with your child, who you want to show gratitude and why?	Practicing gratitude Investing in social connections

##### Manipulation check

Immediately after the simulation tasks, participants provided overall ratings for the three measurements. First, they rated the valence of the imagination scenarios or recall objects on a scale from −3 (Very negative) to 3 (Very positive). Second, participants rated their affective state after completing the tasks on a scale from 1 (Feeling very bad) to 5 (Feeling very good). Finally, participants rated the vividness of their mental images during the practice on a scale from 1 (No mental image at all) to 5 (I have an extremely vivid image in my head).

##### Affective response to pandemic and vaccine‐related news

After completing the imagination or recall tasks, participants were directed to the next page where they rated a new set of three news items. These news items were different from those used for the baseline assessment. Participants were instructed to rate the affective valence of each news item and how they felt after reading each one. Aggregated affective valence scores (Cronbach's *α* = .83) and affective state scores (Cronbach's *α* = .86) were generated as a post‐intervention immediate assessment outcome.

#### Post‐intervention assessment at T2

##### Vaccine‐hesitant attitudes

**FIGURE 2:**
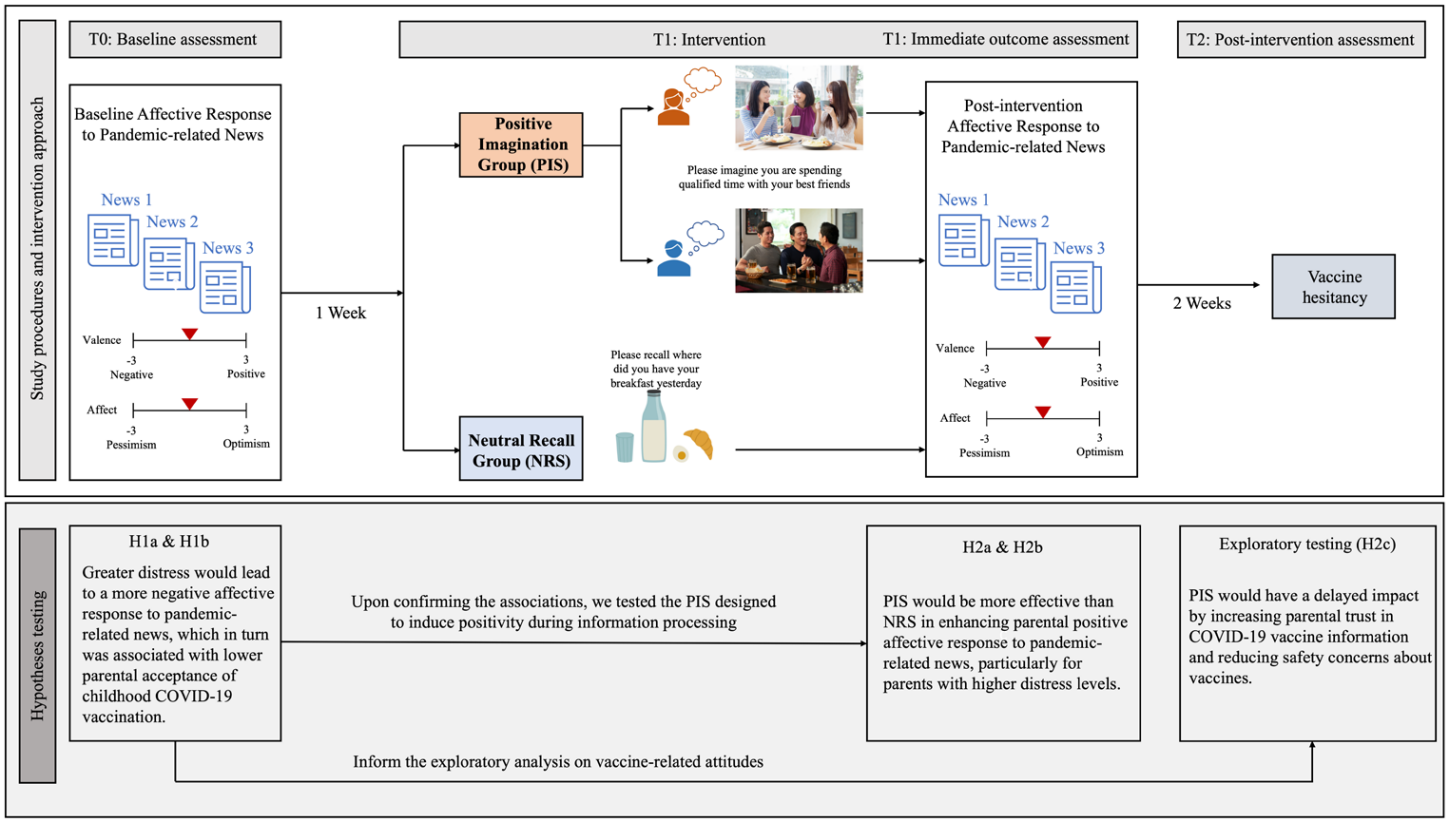
A schematic of the full experimental design and hypotheses testing.

Two weeks after the intervention, we re‐assessed participants' vaccine‐hesitant attitudes towards childhood COVID‐19 vaccines using the same PACV short scale used in the baseline survey (Cronbach's *α* = .80). Figure 2 presents a schematic of the experimental design of the current study, along with our hypotheses testing.

### Statistical analysis

First, we conducted descriptive analyses to examine major demographics and baseline assessments of psychological distress levels, vaccination acceptance and affective response to pandemic and vaccine‐related news. To test H1a, we used the Shapiro–Wilk test to check normality and Spearman correlations to investigate the relationship between psychological distress and affective response to pandemic and vaccine‐related news using the baseline assessment data. To test H1b, we used a structural equation modelling (SEM) to test a mediation model hypothesizing that affective response to pandemic and vaccine‐related news would mediate the association between parental psychological distress and their acceptance of childhood COVID‐19 vaccination. We followed the recommendation of Zhao et al. (2010) to establish our mediation through a single test by generating the bootstrap test of the indirect effect *a* × *b*. We used the weighted least squares mean and variance‐adjusted (WLSMV) estimator to estimate the parameters because the outcome of vaccination acceptance was dichotomized. Bootstrapping with 1000 iterations was conducted to obtain 95% confidence intervals for the indirect effects on Mplus 8.3 (Muthén & Muthén, 2017). We reported the indirect effect size and the proportion of variance accounted for by the mediator to quantify the indirect effect (Rijnhart et al., 2019). We assessed model fit using Comparative Fit Index (CFI), Tucker–Lewis Index (TLI) and Root Mean Square Error of Approximation (RMSEA). A good model fit is characterized by CFI ≥0.90, TLI ≥0.90 and RMSEA ≤0.08 (Kline, 2023).

For intervention effects, we first assessed randomization using the Pearson chi‐square test and independent samples *t*‐tests. For the manipulation check, we used independent *t*‐tests to compare PIS and NRS in inducing positive affect and vivid mental images. We tested H2a using Analysis of Covariance (ANCOVA) on the post‐stimuli affective ratings on news controlled for the baseline ratings. To examine the modification effect of psychological distress level on the intervention's immediate effectiveness (H2b), we used a two‐way ANCOVA model with psychological distress as a moderator. To test H2c, we conducted an ANCOVA to compare each PACV item between the PIS and NRS groups, controlling for the respective baseline item. A recent study used a similar method to analyse each item of the PACV short scale, revealing changes in specific attitudes that address different aspects of vaccine hesitancy (Higgins et al., 2023). We also tested the intervention effect on overall vaccine hesitancy using the aggregated scores of the five items. For H2d, we used PROCESS models in SPSS 26.0 to determine the indirect paths from condition to vaccine‐hesitant attitudes, using valence rating and affective state as an aggregating indicator of affective response to pandemic and vaccine‐related news (Cronbach's *α* = .93) (Preacher & Hayes, 2004). We examined the patterns of missing data for the variables included in primary analyses using Little's MCAR test in SPSS 26.0. The results showed that the data were missing completely at random (MCAR), supported by a non‐significant *p*‐value of .78. Consequently, all missing data were handled using a complete case analysis approach (Little & Rubin, 2019). The immediate effect of the intervention, as well as the effect at the two‐week post‐intervention point, were evaluated using a completer analysis approach, given that the data were MCAR. Other analyses were conducted using R v4.2.3.

## RESULTS

### Baseline assessment

#### Participants

A total of 647 parents completed the baseline assessment (Table 2). Most of our participants were female (86.2%) and aged between 35 and 44 (60.6%). Almost all participants were currently married (92.7%). Approximately half had a full‐time job (49.1%) and an educational attainment of tertiary or above (49.4%). Of the participants, 480 (74.2%) indicated that their child had received COVID‐19 vaccines or that they intended to take their child for the vaccines at the time of our baseline survey. Parental psychological distress at the baseline was 2.55 (SD = 2.50), with 44.8% of parents being categorized as having mild to severe psychological distress based on the cut‐off ≥3 for the PHQ‐4 sum scores. Baseline ratings for news valence evaluation and affective state were 1.36 (SD = 3.51) and 1.24 (SD = 3.42), respectively, indicating that the news items were overall neutral in terms of affective valence.

**TABLE 2:** Baseline characteristics of participants and comparison between the intervention group and the control group (*N* = 647).

Characteristics	The whole sample (*n* = 647)	Intervention/PIS (*n* = 316)	Control/NRS (*n* = 316)	Difference *p* (intervention versus control)^a^
Sex (Female)	554 (86.2%)	273 (86.4%)	274 (86.7%)	.59
Age groups (years)				
18–34	167 (26.0%)	89 (28.5%)	73 (23.2%)	.14
35–44	389 (60.6%)	178 (57.1%)	204 (64.8%)	
Above 45	86 (13.4%)	45 (14.4%)	38 (12.1%)	
Educational attainment				
Secondary or below	326 (50.6%)	160 (51.1%)	157 (49.7%)	.72
Tertiary or above	318 (49.4%)	153 (48.9%)	159 (50.3%)	
Marriage status (being married)	596 (92.7%)	288 (91.7%)	296 (94.3%)	.28
Household monthly income (HKD)^b^				
<30,000	274 (46.4%)	138 (47.6%)	126 (44.1%)	.62
30,000–59,999	198 (33.5%)	97 (33.4%)	98 (34.3%)	
>60,000	119 (20.1%)	55 (19.0%)	62 (21.7%)	
Employment				
Housewife	260 (40.3%)	126 (39.9%)	124 (39.4%)	.56
Part‐time employed	53 (8.2%)	24 (7.6%)	33 (10.5%)	
Full‐time employed	317 (49.1%)	148 (46.8%)	146 (46.3%)	
Unemployed/seeking a job	15 (2.3%)	8 (2.5%)	4 (1.3%)	
Accepting COVID‐19 vaccines for children^c^	480 (74.2%)	234 (74.1%)	233 (73.7%)	.93
Psychological distress level (SD), range	2.55 (2.50), 0–12	2.57 (2.55), 0–12	2.48 (2.41), 0–12	.66
Affective response to the pandemic and vaccine‐related news at baseline				
News valence evaluation rating (SD), range^d^	1.36 (3.51), −9–9	1.40 (3.41), −9–9	1.37 (3.64), −9‐9	.92
Affective state rating after reading the news (SD), range^d^	1.24 (3.42) −9–9	1.32 (3.36), −9–9	1.19 (3.47), −9–9	.64

^a^ Significant difference was assessed using *χ* ^2^ test for categorical variables and independent samples *t*‐tests for continuous variables.

^b^ 1 HKD = 0.13 USD.

^c^ Participants who accepted COVID‐19 vaccines were those whose child had already received a COVID‐19 vaccine or those who reported that they would ‘certainly’, ‘very likely’ or ‘likely’ to take their child for COVID‐19 vaccination in the next 3 months.

^d^ Sum score was calculated by aggregating participants' ratings on the affective response of the three pandemic and vaccine‐related news items.

#### Correlation between psychological distress and affective response to pandemic and vaccine‐related news

We used the baseline data to test H1a that greater parental distress is associated with a more negative affective response to pandemic and vaccine‐related news. Due to the non‐normal distribution of parental distress scores (Shapiro–Wilk *p* < .001), Spearman's correlation was employed to assess their associations. We found that higher psychological distress was significantly correlated with a more negative pandemic and vaccine‐related news valence rating (*r* (637) = −.20, 95% confidence interval [95% CI]: −0.28 to −0.12, *p* < .001), and a more negative affective state rating after reading the news (*r* (637) = −.24, 95% CI: −0.32 to −0.16, *p* < .001). Therefore, H1a is supported.

#### Negative affective response to pandemic and vaccine‐related news mediates the association of parental distress with acceptance of childhood COVID‐19 vaccination

**FIGURE 3:**
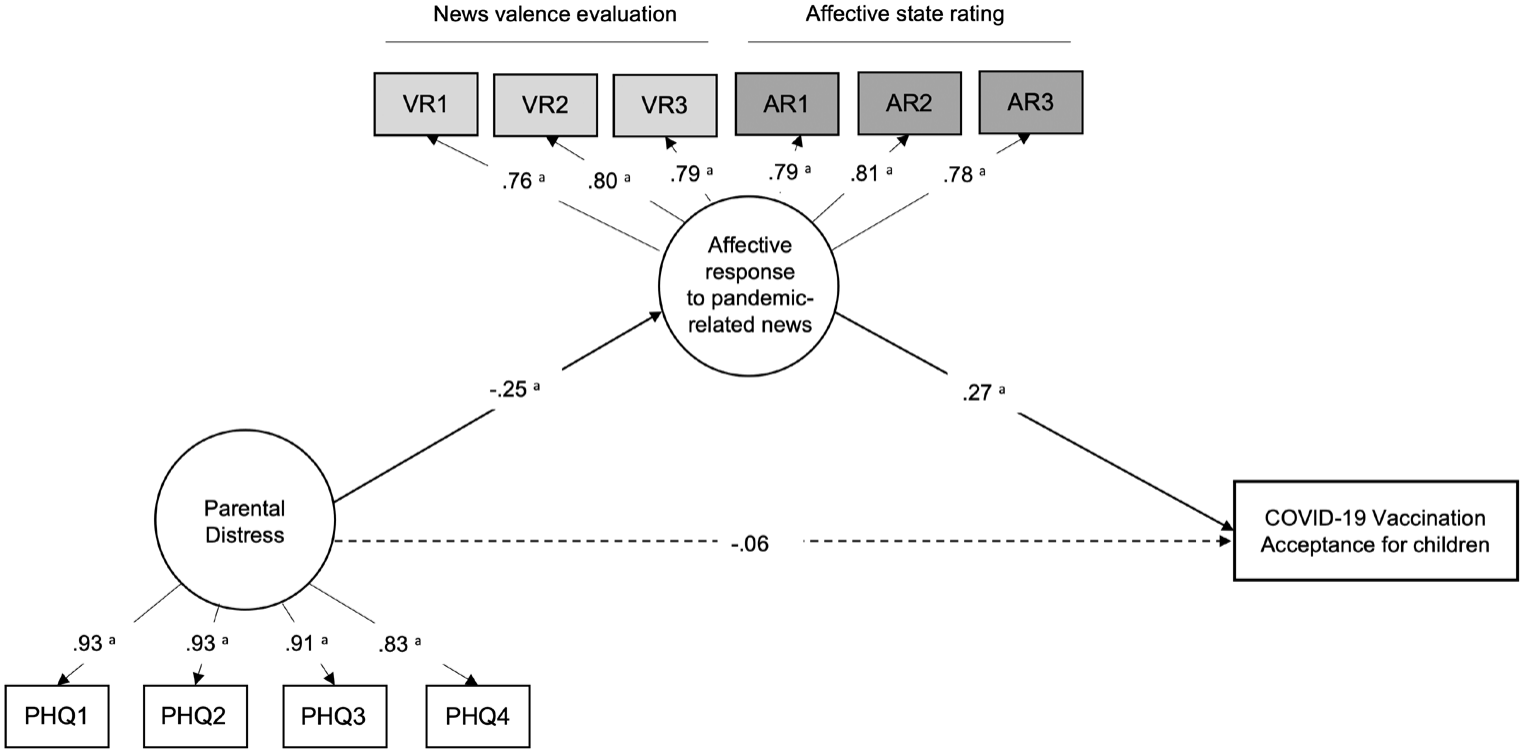
Structural equation model of the association between parental distress level and COVID‐19 vaccination acceptance via affective response to pandemic and vaccine‐related news (*N* = 647). Standardized estimates were provided for each path. The circular shape represents the latent variable and the rectangular shape represents the observed variable. ^a^ *p* < .001. The solid lines indicate statistically significant paths, whereas the dotted lines represent non‐significant results.

We then tested H1b, whether affective response to pandemic and vaccine‐related news mediates the association of parental distress with their acceptance of childhood COVID‐19 vaccination using SEM. Figure 3 shows the SEM results. The model fits well to our data (RMSEA = 0.08 [90% CI: 0.070–0.091], CFI = 0.96, TLI = 0.95). It shows that higher parental psychological distress was associated with more negative affective response to pandemic and vaccine‐related news (*β* = −.25, *p* < .001) which in turn was associated with lower parental acceptance of the COVID‐19 vaccination in children (*β* = .27, *p* < .001). The direct effect of parental distress on acceptance of COVID‐19 vaccination in children was insignificant, indicating an indirect‐only mediation model. The direct effect was 0.06 (95% CI: −0.060 to 0.174), which was insignificant because the confidence interval includes zero. The overall indirect effect was significant at −0.07 (95% CI: −0.117 to −0.038), which explained 54% of the total effect of parental distress via an affective response to pandemic and vaccine‐related news on childhood COVID‐19 vaccination acceptance. Therefore, H1b was supported. Other standardized associations are reported in Table S3.

#### Intervention and immediate outcome assessment

##### Randomization check

In total, 632 participants participated in the intervention and completed the immediate outcome assessment. Participants were randomly allocated to the NRS and PIS conditions at a 1:1 ratio (Figure 1). None of the baseline assessments or demographics differed significantly between the two conditions (Table 2).

##### Manipulation check

**FIGURE 4:**
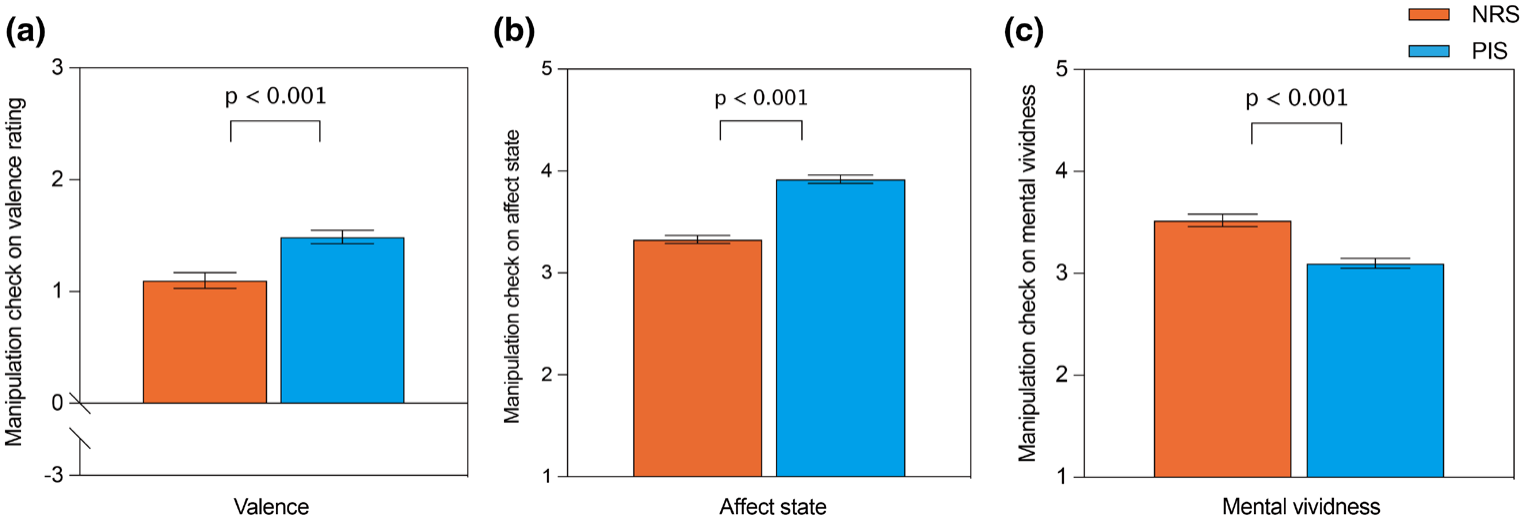
Manipulation checks on valence evaluation, affect state and mental image vividness after completing the imagination or recall tasks between the two conditions. The orange bar represents the mean rating score of the NRS group and the blue bar represents the mean rating score of the PIS group. (a) shows participants' valence rating of stimuli materials immediately post‐stimuli on a bipolar scale from − 3 to 3, where a higher number indicates a more positive valence rating. A gap in the Y‐axis was used to aid the visualization. (b) shows participants' ratings of the affect state immediately post‐stimuli on a 5‐point scale, where a higher number indicates a more positive affective state. Panel C shows participants' ratings of perceived vividness of mental images immediately post‐stimuli on a 5‐point scale, where a higher number indicates greater vividness in generating mental images. Bars and error bars indicate mean ± standard errors.

Compared with the NRS group, those allocated to the PIS group rated the stimuli materials as more positive (NRS: *M* = 1.10, SD = 1.21; PIS: *M* = 1.49, SD = 1.13; *t* (630) = −4.21 and *p* < .001) and reported more positive feelings during the task (NRS: *M* = 3.33, SD = 0.77; PIS: *M* = 3.92, SD = 0.76; *t* (630) = −9.75 and *p* < .001). However, we noticed a greater perceived vividness in generating mental images in the NRS group compared to the PIS group (NRS: *M* = 3.52, SD = 1.00; PIS: *M* = 3.10, SD = 0.96; *t* (630) = 5.31 and *p* < .001) (Figure 4). This may be because participants in the NRS group were instructed to recall specific details regarding their recent experiences (e.g., breakfast they ate yesterday) and familiar objects (e.g., furniture at home), whereas the PIS group was instructed to imagine future scenarios. In summary, we considered that our manipulation was successful in inducing more positive affect among participants in the PIS group.

##### Immediate intervention effect on affective evaluation of the pandemic and vaccine‐related news

**FIGURE 5:**
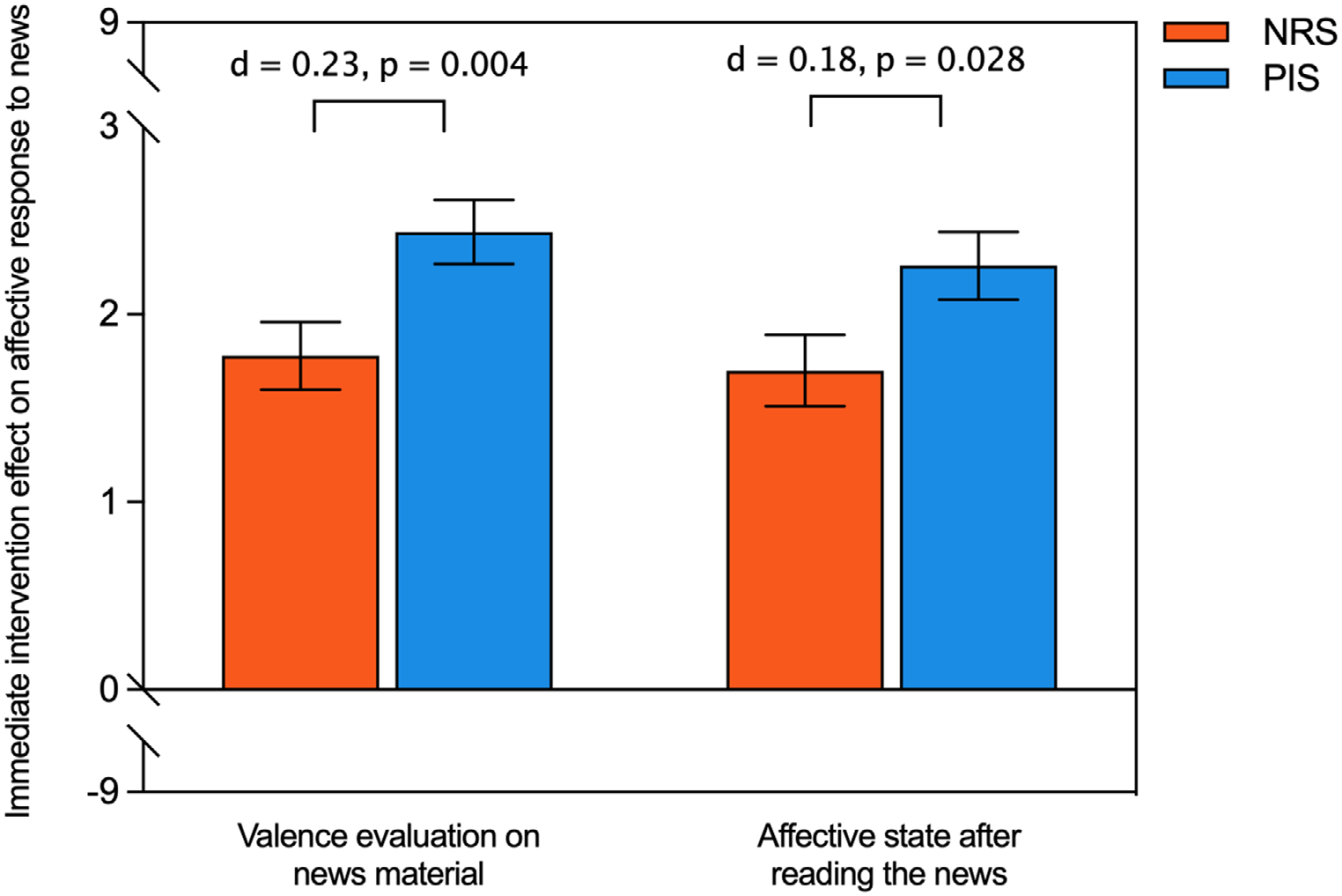
Immediate ratings on news valence evaluation and affective state between the intervention group and the control group. Orange bar represents the mean rating score of NRS group; blue bar represents the mean rating score of PIS group. Gaps in the *Y*‐axis were used to aid interpretation. The sum score of the news valence and affective state rating ranges from −9 to 9, where a higher score indicates a more positive valence and affect rating. Bars and error bars indicate mean ± standard errors. Cohen's *d* and *p*‐value were calculated based on the estimated marginal means in ANCOVA models controlling for baseline ratings.

This study aimed to test H2a that PIS compared with NRS can increase parental positivity when processing pandemic and vaccine‐related information. As Figure 5 shows, ANCOVAs revealed significant main effects of PIS on the post‐intervention rating of news valence (*F*(1, 627) = 8.46, *p* = .004) and their affective state (*F*(1, 627) = 4.88, *p* = .028), indicating that H2a is supported. Notably, we did not observe a significant interaction effect between condition and parental psychological distress levels on affective response to pandemic and vaccine‐related news in the two‐way ANCOVA models, indicating the absence of effect modification by baseline psychological distress level (Table S4). Therefore, H2b was not supported.

#### Post‐intervention assessment at T2

##### Participants at T2

As part of a non‐preregistered exploratory analysis, all participants who had joined the intervention and provided consent for us to contact for a future follow‐up survey were re‐contacted 2 weeks after the intervention. Of the 632 participants, 328 (52%) responded to this post‐hoc assessment survey, including 159 in the PIS group and 169 in the NRS group (Figure 1). Baseline assessments and demographics were comparable between the participants who completed the T2 assessment and those who did not (Table S5).

##### Impact on specific vaccine‐hesitant attitudes at 2 weeks post‐intervention

**FIGURE 6:**
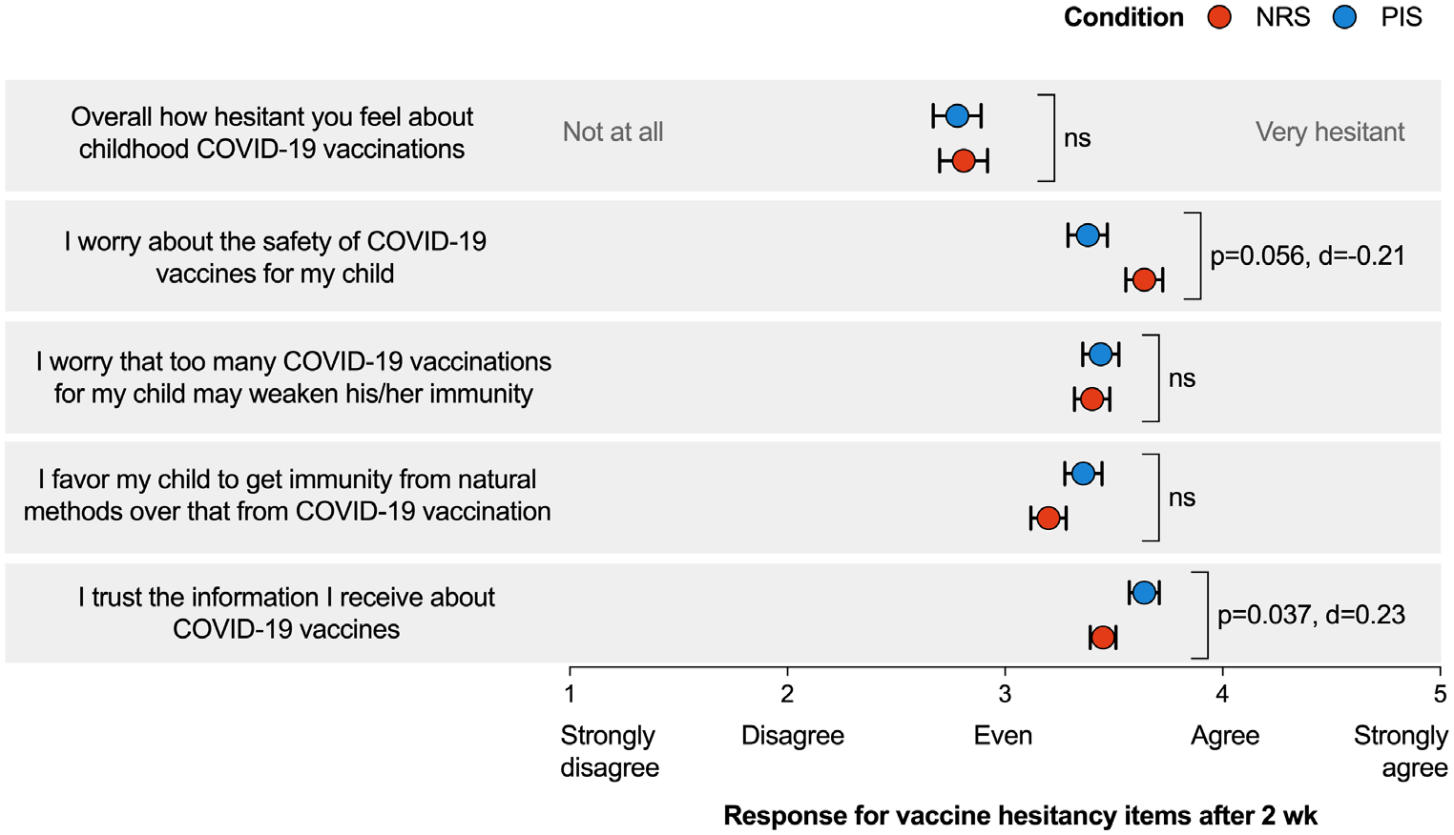
Post‐intervention assessment for vaccine‐hesitant attitudes towards childhood COVID‐19 vaccinations between the intervention group and the control group. Analyses were conducted among 328 parents who completed the T2 assessment. Points show the mean responses (with a 95% CI) between the conditions. ‘ns’ indicates no significant difference between conditions. The orange and blue points represent the mean rating scores for the NRS and PIS groups, respectively. Cohen's *d* and *p*‐values were calculated based on the estimated marginal means in ANCOVA models controlling for baseline ratings.

The post‐hoc assessment was aimed at testing H2c. We conducted an exploratory analysis to examine the impact of our intervention on parents' vaccine‐hesitant attitudes after 2 weeks. After accounting for the influence of baseline vaccine‐hesitant attitudes as covariates, our analysis revealed significant differences between the NRS and PIS groups in two specific vaccine‐hesitant attitudes 2 weeks after the intervention (Figure 6). Specifically, the main effect of the PIS significantly improved trust in COVID‐19 vaccine‐related information (*F*(1, 323) = 4.39, *p* = .037), and the reduction in COVID‐19 vaccine safety‐related worry was marginally significant (*F*(1, 323) = 3.68, *p* = .056). However, there were no significant differences between the conditions in the overall vaccine hesitancy rating or value‐based attitudes (e.g., preference for natural immunity) (*p‐values* ranged from .09 to .88). Using the composite score by aggregating all five attitude items did not reveal a significant group effect (*p* = .41). Overall, H2c was supported, indicating an effect of the PIS in enhancing trust in vaccine‐related information and reducing parental concerns about vaccine safety at a 2‐week post‐intervention point.

##### Post‐intervention effectiveness on vaccine‐related attitudes through an increase in positivity during information processing

**FIGURE 7:**
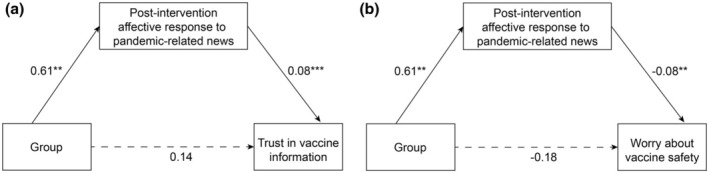
Path analysis model of the association between condition and specific vaccine‐hesitant attitudes via affective response to pandemic and vaccine‐related news (*N* = 328). Standardized estimates were provided on each path. The solid lines indicate statistically significant paths, whereas the dotted lines represent non‐significant results. **p < .01 and ***p < .001. (a) used trust in COVID‐19 vaccine‐related information as a dependent variable, controlling for baseline trust rating. (b) used worry about COVID‐19 vaccine safety as the dependent variable, controlling for baseline safety concern rating.

In H2d, we hypothesized that improving positive affective evaluation during parental information processing would lead to increased trust in vaccine‐related information and reduced concerns regarding vaccine safety. To assess this, we aggregated the valence evaluation rating score and affective state rating score at each news item level and then calculated a mean score across all post‐stimuli news items. It served as an overall indicator of the post‐intervention affective response to pandemic and vaccine‐related news. Figure 7 shows that the impact of the PIS on increasing trust in vaccine‐related information (indirect effect: 0.05, 95% CI: 0.01 to 0.10) and reducing vaccine safety concerns (indirect effect: −0.05, 95% CI: −0.13 to −0.01) was fully mediated by the increase in positivity observed in the post‐intervention affective response to pandemic and vaccine‐related news, even after adjusting for baseline trust or safety‐related ratings. Therefore, H2d was supported.

## DISCUSSION

Our study revealed that parental psychological distress was associated with a more negative evaluation of pandemic and vaccine‐related news, which in turn correlated with lower acceptance of childhood COVID‐19 vaccination. To address distress‐induced negativity in information processing, we utilized a randomized online experiment to assess the impact of a PIS intervention on enhancing positivity in information processing. We observed an immediate increase in the positive evaluation of pandemic and vaccine‐related news in the PIS group compared to the control group. Furthermore, improving positive affective evaluation in parental information processing helped to further enhance trust in vaccine‐related information and alleviate vaccine safety concerns 2 weeks after the intervention.

Recent studies have demonstrated that psychological distress diminishes vaccination acceptance by increasing beliefs in conspiracies, mistrust in medical science (Simione et al., 2021) and inducing negativity during information processing (Yuan, Dong, et al., 2023; Yuan, Lam, et al., 2023). Our current study demonstrated that parents with heightened psychological distress exhibited greater negativity in their information processing, as indicated by their more negative ratings of the valence of real‐world news and their affective state after reading the news. The heightened negativity in information processing may be regarded as a bias, as the news presented was neutral overall. Moreover, this negativity bias in information processing was linked to a tendency to refuse or delay vaccination, as indicated by low vaccination acceptance. Existing literature has consistently found an association between parental psychological distress and vaccine hesitancy, but fails to explain why this association exists (Horiuchi et al., 2021; Ie et al., 2023; Xu et al., 2021). Our study indicates that a potentially plausible psychological mechanism through which psychological distress increases vaccine hesitancy may be that psychological distress induces a more negative affective response to the pandemic and vaccine‐related news. Such information processing biases lead to an automatic evaluative process by encoding incoming stimuli with a more negative valence (Ferguson et al., 2005). Therefore, reducing parents' negative information processing is critical to enhancing childhood vaccination acceptance.

Our study also demonstrated that imagining personally relevant positive life scenarios led to increased positive affective responses during subsequent information processing. This effect applied to parents with low and high levels of distress. Studies have consistently found that emotional factors, rather than cognitive factors, exert a greater influence on preventive and vaccination behaviours, especially during stressful and uncertain pandemic contexts (Sobkow et al., 2020; Yuan, Dong, et al., 2023; Yuan, Lam, et al., 2023). This holds particularly true for distressed individuals, who rely on affective heuristics for complex decisions (Finucane et al., 2000). Our intervention leveraged positive psychology and personalized imagery techniques to enhance parental positivity. A previous systematic review demonstrated that positive psychology activities, such as envisioning the future, emphasizing social connections and expressing gratitude, can significantly increase participants' positive affect (Bolier et al., 2013). Tailoring these activities to individuals' characteristics further enhances engagement and positive affect (Lyubomirsky & Layous, 2013) by improving involvement in self‐relevant scenarios during imagery tasks (Schubert et al., 2020). Studies found that personalized mental images elicit stronger positive emotions compared to less detailed elaborative imagination (Demeyer & De Raedt, 2014; Schubert et al., 2020). Our study demonstrated that incorporating personalized images and providing step‐by‐step guidance increased positive responses during subsequent information processing. Interestingly, the control group, which recalled neutral things in daily life, also showed a slight increase in positive emotions. This could be due to shifting people's attention away from negative stimuli, although such an effect is more evident by making positive stimuli salient in one's mind (Holmes et al., 2009). We found that the pandemic situation was generally similar at T0 and T1 (Figure S1), thereby minimizing the potential impact of external factors (e.g., improvement in pandemic situations) on the observed changes in participants' affective responses to news.

Our intervention had a spillover impact on reducing parental safety concerns about COVID‐19 vaccination and increasing trust in vaccine‐related information after 2 weeks. Our path analysis showed that this was due to participants' more positive processing of information after completing the positive imagination tasks, leading to a more positive evaluation of other pandemic‐related information, including COVID‐19 vaccine‐related information. Although our intervention did not include any vaccine‐related information, it is possible that our survey contained vaccine‐related cues that subtly primed parents to associate the intervention with vaccination. Sustained positivity can maintain parents' attention to positive stimuli and facilitate positive evaluative processes, thereby improving trust in vaccine‐related information and alleviating concerns about vaccine safety. Although the latter outcome should be interpreted with caution due to its marginal significance (*p* = .056), the directionally positive change provides initial evidence that positive affect priming has the potential to spillover and influence how people interpret COVID‐19 vaccines. These findings are also consistent with a recent study on the sustained impact of imagination simulation on improving risk appraisals by enhancing learning from emerging information (Sinclair et al., 2021). We did not observe significant changes in overall parental vaccine hesitancy or value‐based attitudes, such as a preference for purity and distrust in overmedication. These deeply held attitudes were less likely to be altered solely by positive information processing. Instead, future interventions targeting parents' underlying values may be necessary to address parents' vaccine hesitancy due to attitude roots (Hornsey et al., 2018).

### Practical implications for future risk communication

Our study suggests that positive framing, as opposed to fear or threat appeal, holds promise for mitigating negative emotions and addressing vaccine hesitancy during a pandemic when people have already suffered from multiple stressors (Bavel et al., 2020). By incorporating positive cues, we can reduce distress‐induced negativity in information processing and direct parental attention towards the positive aspects of vaccine‐related information. Furthermore, we observed a significant improvement in vaccine‐related attitudes after 2 weeks of the intervention, and this improvement was mediated by the increased positivity in post‐intervention news evaluation. This highlights the potential effectiveness of utilizing a light‐touch communication style to induce positive affect instead of solely providing information. In contrast, the traditional ‘hard‐sell’ approach by repeatedly emphasizing the benefits of vaccination may lead to psychological reactance, particularly when individuals become fatigued with excessive vaccine‐related information (Stamm et al., 2023). Additionally, positive imagination storytelling can be integrated into healthcare settings, where doctors can guide parents to share positive life stories before providing vaccine‐related information to enhance parental vaccination decisions for children (Tates & Meeuwesen, 2001). It can also be implemented in large‐scale online intervention, offline campaigns and even media narratives, where vaccine‐related information can be arranged following positive cues or information, or in parallel, explicitly, or implicitly, instead of merely presenting factual and risk‐based information.

### Limitations

Our study has some limitations. First, potential factors that may modify the effects of intervention effectiveness, such as pandemic‐related media consumption habits and imagination ability, were not examined in our study. Second, we observed a relatively high dropout rate at T2; this sample size should be able to detect a small effect size of *d* = 0.30 with 80% power. However, the observed effect sizes were 0.21 and 0.23, which suggest the potential for our findings to be underpowered. Therefore, the impacts of our intervention on parental vaccine hesitant attitudes should be interpreted with caution and warrant replication in future research with larger sample sizes. Third, our study focused on parents' pandemic and vaccine‐related information processing and COVID‐19 vaccination decisions for their children, generalizability to other contexts requires further investigation. Fourth, a significant increase in COVID‐19 confirmed cases was observed during the T2 data collection period (Figure S1), which may have influenced parents' perceived risk of COVID‐19 and their vaccination attitudes. However, since data collection for the intervention and control groups occurred concurrently, this contextual factor is unlikely to be a concern for confounding effects. Furthermore, we only compared the effects of PIS versus NRS, which also induced vivid pictures that might affect parents' information processing. Future research should use a third ‘control’ condition by presenting existing factual or fear‐appeal information to participants to comprehensively assess which communication strategies in the pandemic context yield better effects. It is important to note that over 90% of our participants were married, a percentage comparable to that of the general population (Social Indicators of Hong Kong, 2018). However, due to the small sample size of unmarried parents, we were unable to compare the intervention effects between married and unmarried groups. Future research should specifically examine the effects on unmarried parents, as they might experience greater psychological distress.

## CONCLUSION

The baseline data revealed that negative information processing contributed to parental vaccine hesitancy, providing an opportunity to enhance positivity in information processing and address vaccine‐hesitant attitudes. In our subsequent randomized controlled online experiment, we demonstrated the immediate effectiveness of a novel positive imagination intervention in fostering parental positivity during subsequent information processing. This positive affect priming resulted in increased trust in vaccine‐related information and alleviated concerns about vaccine safety 2 weeks after the intervention. Our findings highlight the promising effect of positive affect priming to reduce negativity in information processing and, hence, vaccine‐hesitant attitudes.

## AUTHOR CONTRIBUTIONS

**Jiehu Yuan:** Conceptualization; methodology; data curation; investigation; formal analysis; visualization; project administration; writing – original draft. **Meihong Dong:** Conceptualization; data curation; project administration; writing – review and editing. **Wendy Wing Tak Lam:** Conceptualization; writing – review and editing. **Qiuyan Liao:** Conceptualization; methodology; data curation; investigation; supervision; funding acquisition; writing – review and editing.

## CONFLICT OF INTEREST STATEMENT

The authors have no conflicts of interest to declare.

## Supporting information


Data S1.

## Data Availability

The data that support the findings of this study, as well as the codes for reproducing the main results, are available in Open Science Framework at: https://osf.io/6m59u/?view_only=6082caf8866e4cd49d133458f023184f.
