# Supplementary material for: Using positive imagination to reduce negativity in information processing and hesitant attitudes towards childhood COVID‐19 vaccinations in parents: A randomized controlled trial
Source: Br J Health Psychol. 2025 Jun 3;30(3):e12808. doi: 10.1111/bjhp.12808 (PMC12134526; doi:10.1111/bjhp.12808)
Supplement: Supplementary file 1 — Data S1. [file BJHP-30-0-s001.docx]

**Supplementary: Using positive imagination to reduce negative information processing and parental vaccine-hesitant attitudes: a randomized controlled trial**

Contents

[Supplementary File 1: News Materials 2](#_Toc176184425)

[News Materials Before the Stimuli 2](#_Toc176184426)

[News Materials After the Stimuli 5](#_Toc176184427)

[Supplementary File 2: Manipulation Materials 8](#_Toc176184428)

[Positive Imagination Simulation (PIS) Materials 8](#_Toc176184429)

[**PIS Female Version** 8](#_Toc176184430)

[**PIS Male Version** 22](#_Toc176184431)

[Neutral Recall Simulation (PIS) Materials 36](#_Toc176184432)

[Supplementary Table 44](#_Toc176184433)

[Table S1. Comparison of participants demographics between the two samples using different recruitment methods 44](#_Toc176184434)

[Table S2. Key Measurement in the surveys 46](#_Toc176184435)

[Table S3. Standardized model results of associations between variables in the Structural Equation Model 49](#_Toc176184436)

[Table S4. Two-way ANCOVAs for affective response to pandemic-related news to assess potential modification effect with psychological distress 50](#_Toc176184437)

[Table S5. Comparison of participants demographics across the three time points 51](#_Toc176184438)

[Supplementary Figure 54](#_Toc176184439)

[Figure S1. Number of confirmed cases during study period 54](#_Toc176184440)

[Figure S2. Childhood COVID-19 vaccination uptake rates by the study periods and doses in Hong Kong 55](#_Toc176184441)

# Supplementary File 1: News Materials

The below paragraphs show the news rating tasks in our online survey. All the news was originally presented in traditional Chinese to the participants, with random order.

## News Materials Before the Stimuli

**Instruction for Participants:**

Below you will find three brief news articles regarding the COVID-19 pandemic. Each article will be presented on a separate page. Please read through all of them at your own pace and select the options that best describe your feelings at the end.

#News 1 on Page 1:

It has been two years and three months since the fifth wave of the epidemic began in January. Watching the news daily and confronting various unknowns has led many people to experience pandemic fatigue, anxiety, and depression. Additionally, approximately 3,500 restaurants have temporarily closed following the announcement of a dine-in service ban in January. During this period, many employees contracted the virus, preventing restaurants from operating while still having to pay the rent. As of March, 500 different chain groups and small restaurants have permanently shut down.

Currently, the government is working to provide free new COVID-19 oral medications, specifically Merck & Co., Inc.’s Molnupiravir and Pfizer’s Paxlovid, to private medical practitioners. The involvement of private doctors is expected to help control the pandemic, as the public will have an additional avenue to seek medical treatment, allowing those diagnosed to obtain oral medicine promptly. Additionally, studies have shown that these oral drugs can reduce hospital admissions and mortality by 70%. Even if a sixth wave of COVID-19 occurs later, the medical system is expected to cope with the pressure.

NV1: How positive or negative do you think this news is? (You can drag the slider below)

-3 ———————————————●——————————————— 3

| Very negative | Neutral | Very positive |
| --- | --- | --- |

NA1: How do you feel about this news?

-3 ———————————————●———————————————3

| Very pessimistic | Neutral | Very optimistic |
| --- | --- | --- |

[Click and enter to next page]

#News 2 on Page 2:

The Department of Health has indicated that the current number of confirmed COVID-19 cases remains relatively high, suggesting that numerous transmission chains persist within the community. Citizens should not assume that the pandemic situation has improved. On the contrary, it is important for individuals to remain vigilant and carefully comply with the social distance measures. Experts have cautioned against dismissing the likelihood of a new wave of the pandemic reoccurring at any time. Yuen Kwok-yung, a government expert consultant and chair professor of the Department of Microbiology at the University of Hong Kong, has predicted that a sixth wave of the pandemic could occur within the next 6 to 9 months, potentially resulting in approximately 1,500 deaths.

Recently, the Hong Kong government has been actively promoting and enhancing the use of Traditional Chinese medicine in combating the pandemic. The Kai Tak Holding Centre, which accepts elderly individuals with mild COVID-19 symptoms, is working to integrate treatments involving both Traditional Chinese and Western medicine, with support from a Chinese medicine team from Hong Kong Baptist University. The university has reported that Traditional Chinese medicine is effective in treating symptoms such as coughing and phlegm. Meanwhile, the World Health Organization has noted that Traditional Chinese medicine can effectively manage COVID-19 by preventing mild and common symptoms from developing into severe cases, shortening the time needed to clear the virus, and improving the clinical prognosis for patients with mild and common symptoms.

NV2: How positive or negative do you think this news is? (You can drag the slider below)

-3 ———————————————●——————————————— 3

| Very negative | Neutral | Very positive |
| --- | --- | --- |

NA2: How do you feel after reading this news?

-3 ———————————————●——————————————— 3

| Very pessimistic | Neutral | Very optimistic |
| --- | --- | --- |

[Click and enter to next page]

#News 3 on Page 3:

The Hospital Authority announced that since the beginning of March, 13 children who recovered after being diagnosed with COVID-19 have developed multi-system inflammatory syndrome. The ages of these patients range from 3 to 11 years old, and seven of them were admitted to the intensive care unit. None of these children had any pre-existing conditions. Symptoms appeared approximately two to five weeks after recovery and included persistent fever, red and cracked lips, and skin rashes. In severe cases, the children experienced heart failure and required cardiotonic drugs for additional support. More than half of the affected children had not been vaccinated.

Doctors have indicated that children may experience a strong immune response after being infected with the COVID-19 virus. Data show that vaccination can reduce the risk of post-COVID-19 complications by 90%. Statistics demonstrate that vaccinated children aged 5 to 11 years can reduce the risk of infection and hospitalization by more than twofold compared to unvaccinated children, and the risk of death can be reduced by nearly tenfold. Children who received two doses of the vaccine had 90.7% protection against symptomatic COVID-19 infection.

NV3: How positive or negative do you think this news is? (You can drag the slider below)

-3 ———————————————●——————————————— 3

| Very negative | Neutral | Very positive |
| --- | --- | --- |

NA3: How do you feel after reading this news?

-3 ———————————————●——————————————— 3

| Very pessimistic | Neutral | Very optimistic |
| --- | --- | --- |

[Click and enter to next page]

## News Materials After the Stimuli

**Instruction for Participants:**

Below you will find three brief news articles regarding the COVID-19 pandemic. Each article will be presented on a separate page. Please read through all of them at your own pace and select the options that best describe your feelings at the end.

#News 1 on Page 1:

The Centre for Health Protection announced that as of midnight today (April 5^th^, 2022), schools have reported 26 positive COVID-19 cases, involving 24 students and 2 staff members. These individuals are from one kindergarten, 13 primary schools, and 11 secondary schools. In addition, public hospitals reported 5 new cases, with a fatality rate of 0.76%, bringing the total number of deaths to 9,120 in this wave.

Experts indicate that vaccination with the Sinovac or BioNTech vaccines can reduce the risk of severe illness and death. Globally, more than 100 million schoolchildren have been vaccinated. The efficacy rate of the BioNTech vaccines against new coronavirus variants is 90.7%. According to the U.S. Centre for Disease Control and Prevention, the BioNTech vaccine is 98% effective in preventing adolescents from needing intensive care unit admission. The likelihood of deaths following vaccination is also 10-15 times lower compared to unvaccinated individuals.

NV1_b: How positive or negative do you think this news is? (You can drag the slider below)

-3 ———————————————●——————————————— 3

| Very negative | Neutral | Very positive |
| --- | --- | --- |

NA1_b: How do you feel after reading this news?

-3 ———————————————●———————————————3

| Very pessimistic | Neutral | Very optimistic |
| --- | --- | --- |

[Click and enter to next page]

#News 2 on Page 2:

The number of COVID-19 cases in Hong Kong is gradually declining, but new cases continue to emerge. The Department of Health and the Hospital Authority held a press conference on the epidemic this afternoon (April 5^th^, 2022). As of midnight today, there were 321 newly confirmed cases in Hong Kong, bringing the total number of cases in the fifth wave of the epidemic to 1,193,086.

Merck’s oral drug Molnupiravir, currently used to treat COVID-19, has begun introduced by the Hospital Authority. Public hospitals and institutions have prescribed 1,600 courses of treatment. Data indicate that 80% of patients have stabilized and have not experienced a worsening of their condition. According to research conducted by pharmaceutical companies, taking the drug can reduce the risk of hospitalization by 30%.

NV2_b: How positive or negative do you think this news is? (You can drag the slider below)

-3 ———————————————●——————————————— 3

| Very negative | Neutral | Very positive |
| --- | --- | --- |

NA2_b: How do you feel after reading this news?

-3 ———————————————●——————————————— 3

| Very pessimistic | Neutral | Very optimistic |
| --- | --- | --- |

[Click and enter to next page]

#News 3 on Page 3:

The Omicron variant has led to the emergence of numerous subtypes worldwide. In Hong Kong’s fifth wave of the pandemic, the BA.2 subtype of Omicron is prevalent and has further evolved into dozens of subtypes, such as BA.2.1.1, indicating slight viral mutations. The Omicron subtypes BA.4 and BA.5, which have been detected in other countries, may enter the local community in Hong Kong at any time, potentially causing a significant rebound in the pandemic.

Government expert advisor David Hui Shu-cheong has stated that the impact of the mutant virus is minimal. The UK has recorded 700 cases since January, primarily involving Omicron, with a short-term incidence. Even with viral mutations, the vaccine remains effective in reducing severe illness and death. The T-cell response elicited by the vaccine provides protection against severe illness. Three doses of the vaccine can offer more than 90% of protection.

NV3_b: How positive or negative do you think this news is? (You can drag the slider below)

-3 ———————————————●——————————————— 3

| Very negative | Neutral | Very positive |
| --- | --- | --- |

NA3_b: How do you feel after reading this news?

-3 ———————————————●——————————————— 3

| Very pessimistic | Neutral | Very optimistic |
| --- | --- | --- |

[Click and enter to next page]

# Supplementary File 2: Manipulation Materials

## Positive Imagination Simulation (PIS) Materials

Participants assigned to the PIS group were provided with the following instruction:

[SN] First, what is your gender?

- Female (Direct to Female PIS)
- Male (Direct to Male PIS)

In the following interactive task, you will engage with several sets of images designed to help you imagine three different life scenarios that could happen to you. After viewing the images, you will answer a few short questions to assess your imagination ability. This section takes approximately 6 minutes to complete. You will be prompted to pause for several seconds to fully engage in the imagination process. Please proceed with the task at your own pace.

### **PIS Female Version**

**[Next page: Scenario 1]**


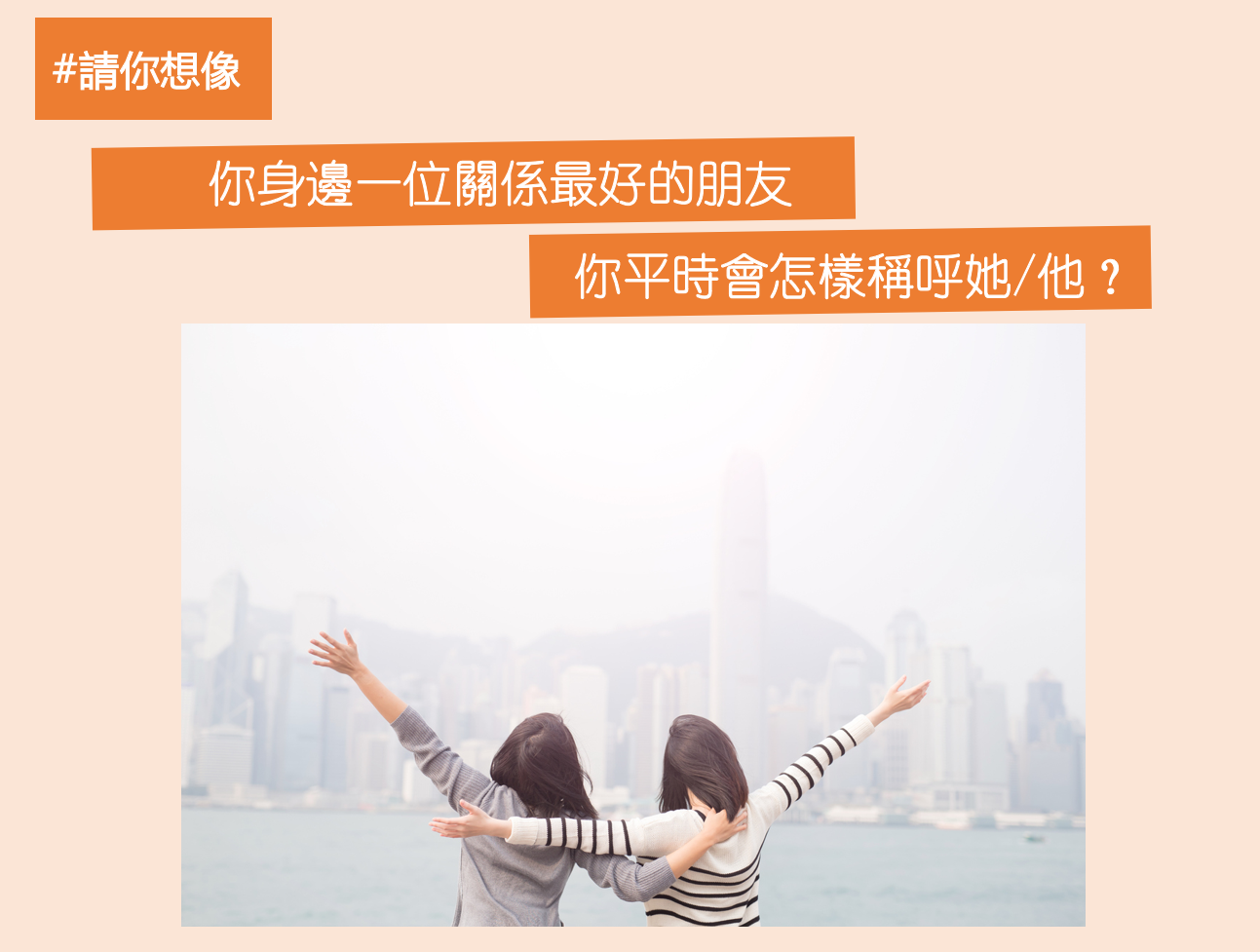


[English]: Please imagine one of your closest friends, how do you usually call him/her?


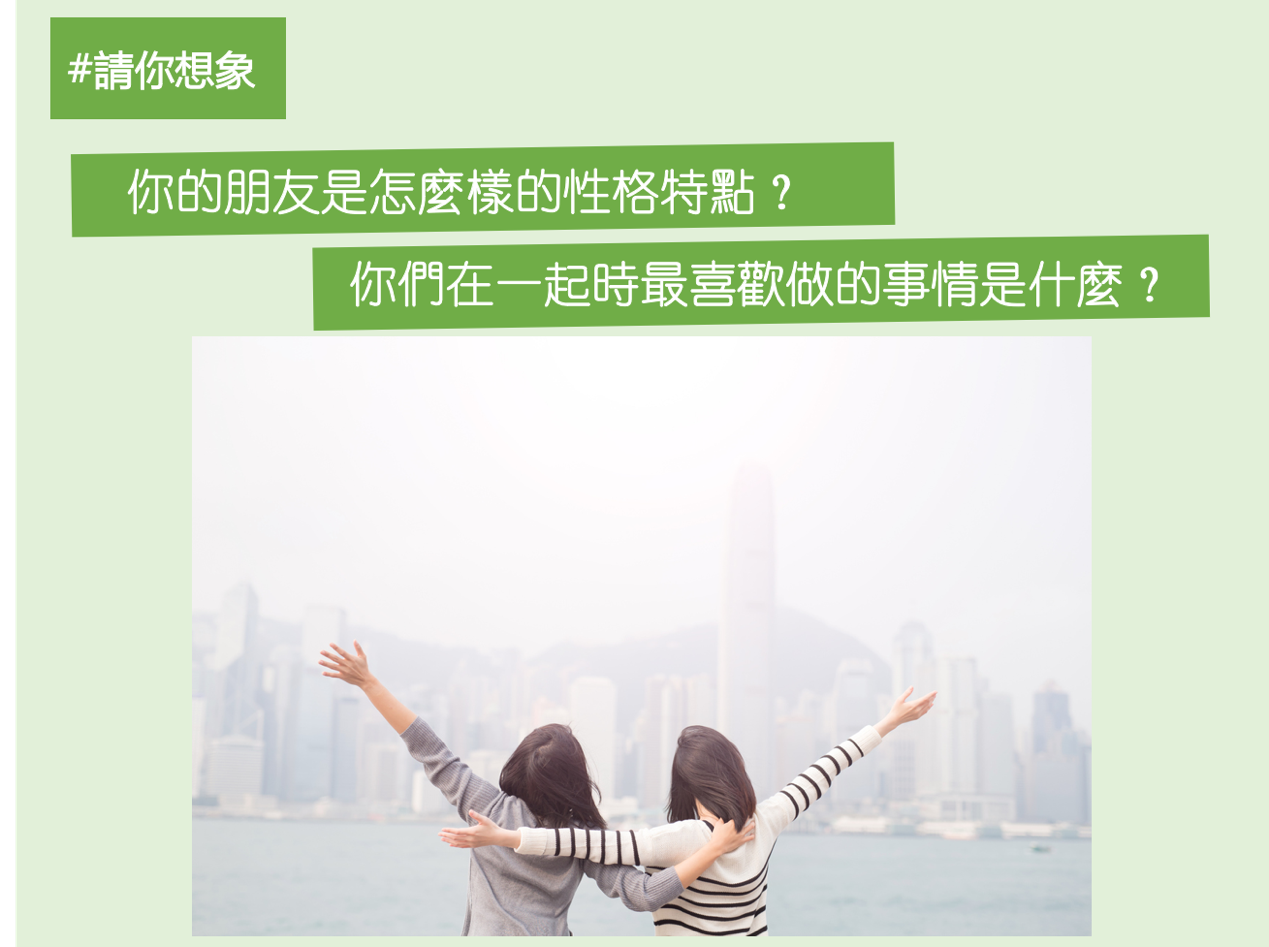


[English]: Please imagine the personality traits of your friend. What are your favorite activities to do together?


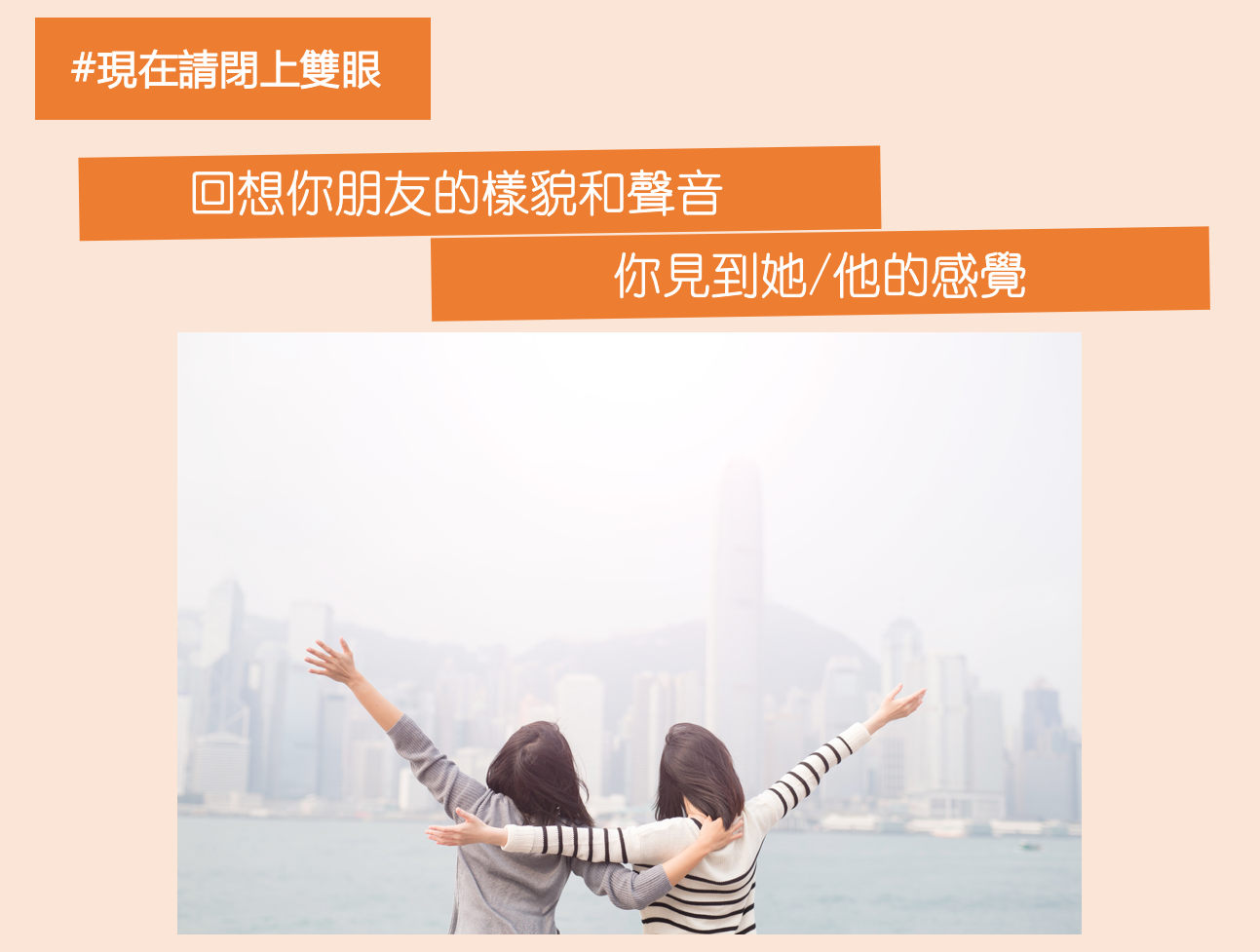


[English]: Please recall the appearance and voice of your friend. How do you feel when you see him/her?

[Pause for 5s before the next page bottom appear]


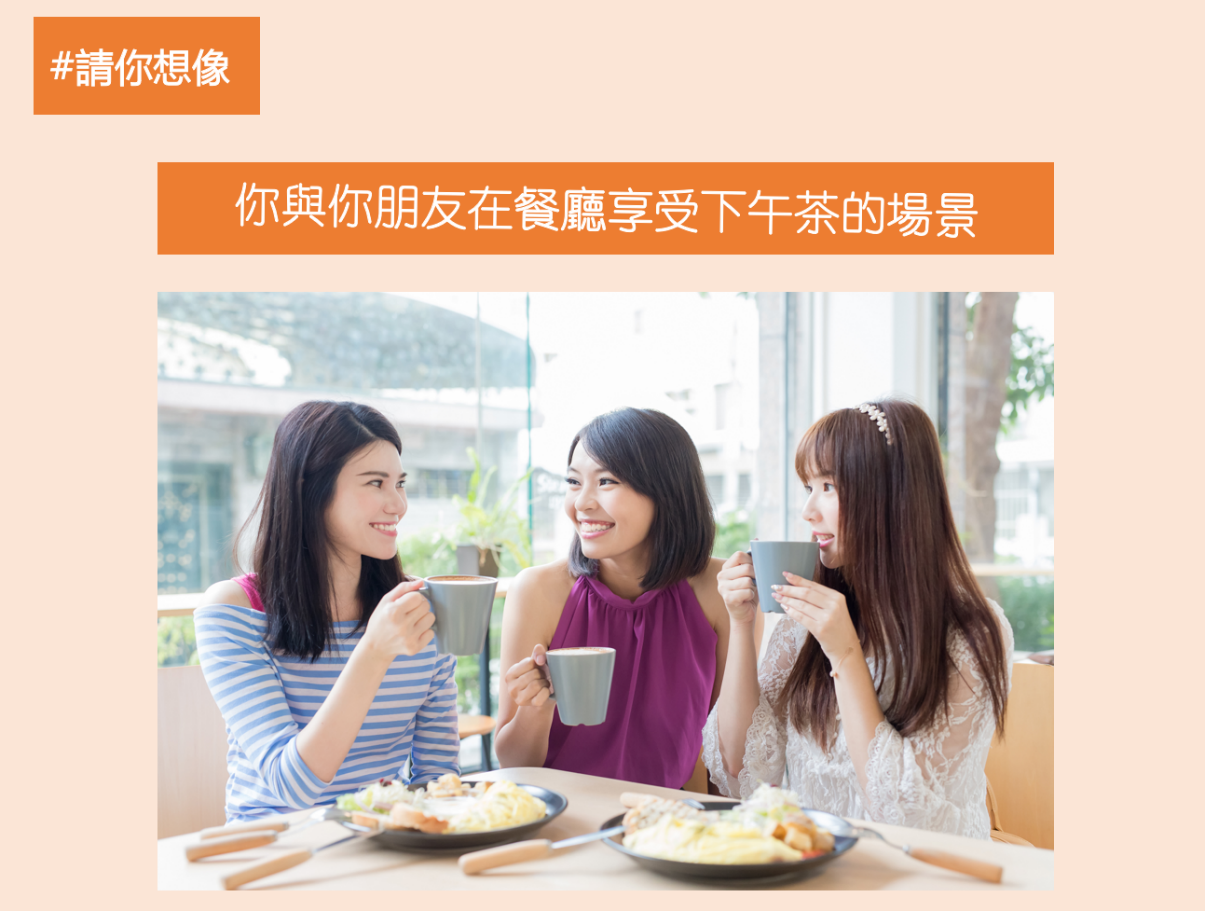


[English]: Now please imagine you and your best friend is having afternoon tea together at a restaurant.


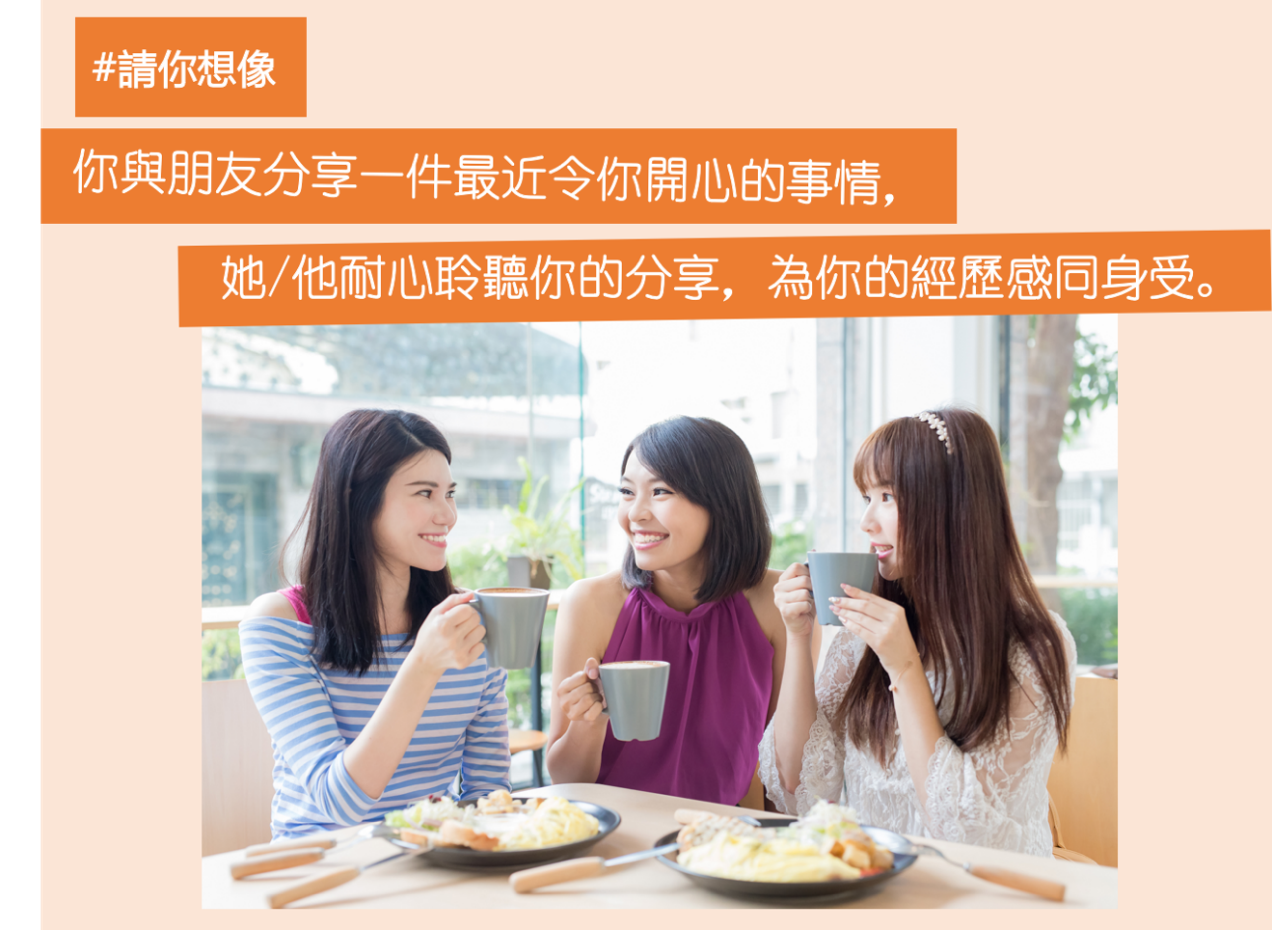


[English]: Continue to imagine sharing a recent happy event with your friend. He/she listens patiently to your story and empathizes with your experience.


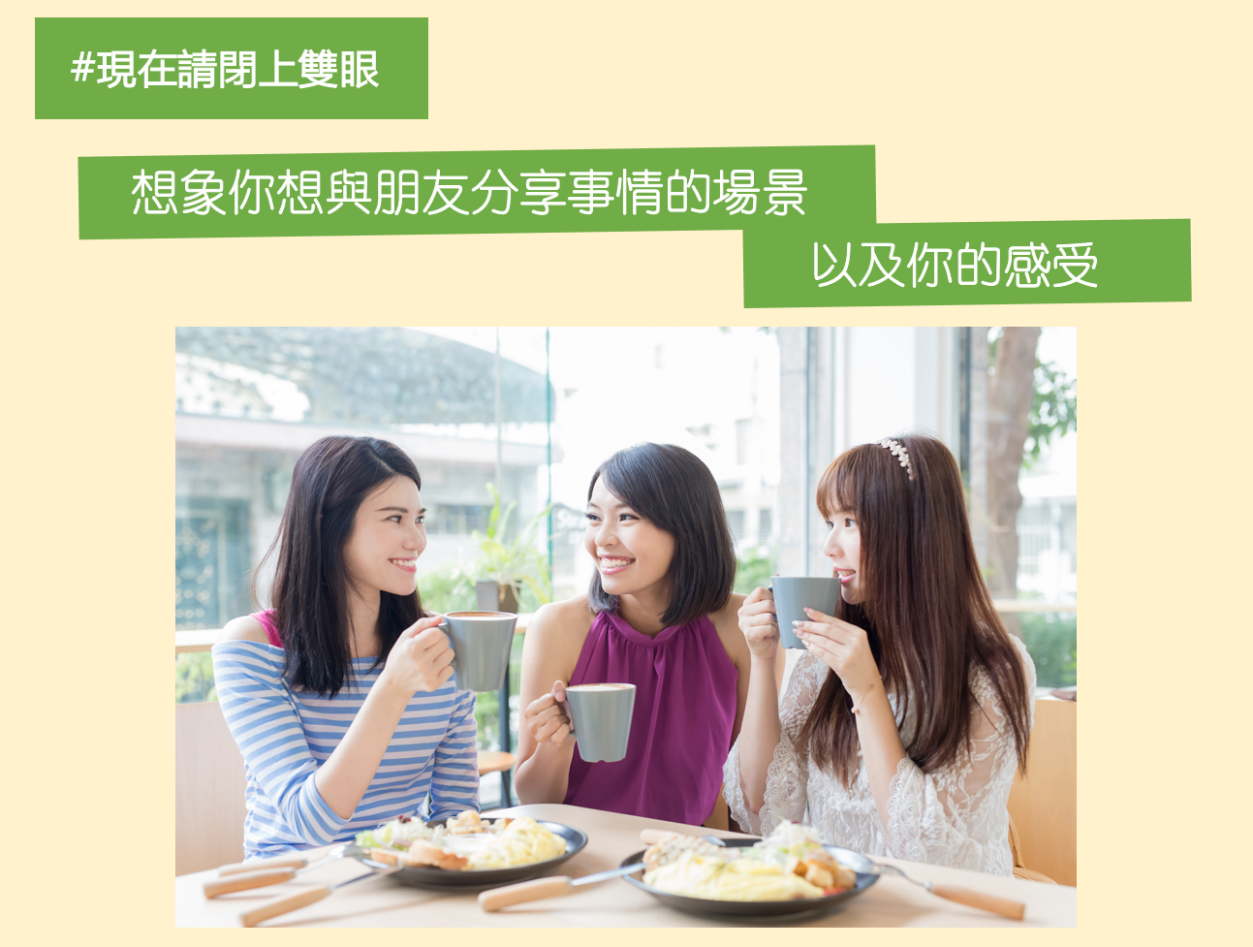


[English]: Now please close your eyes. Imagine this scenario and how you feel about it.

[Pause for 5s before the next page bottom appear]

**[Next page: Scenario 2]**


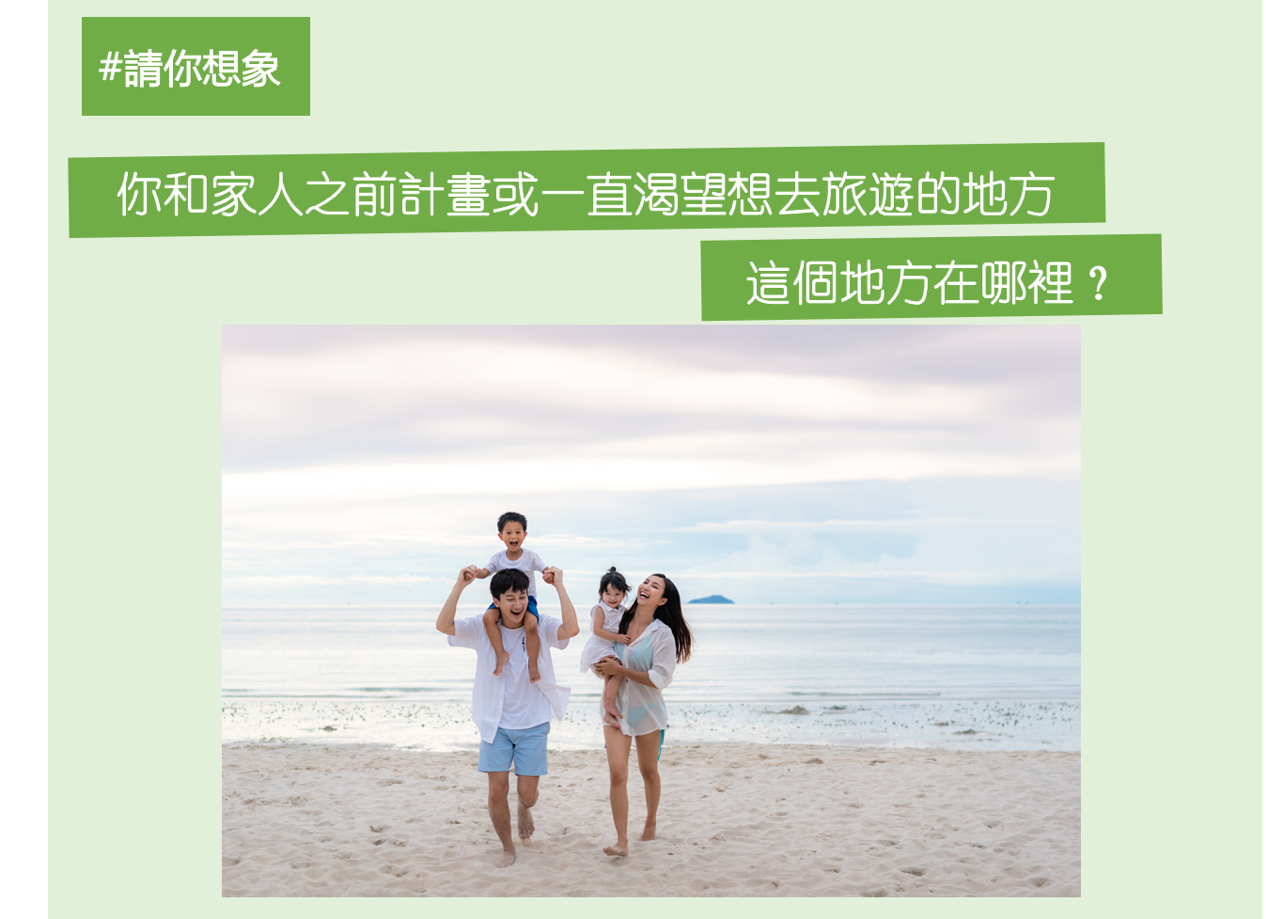


[English]: Please imagine a place you and your family have planned or always wanted to visit. Where is this place?


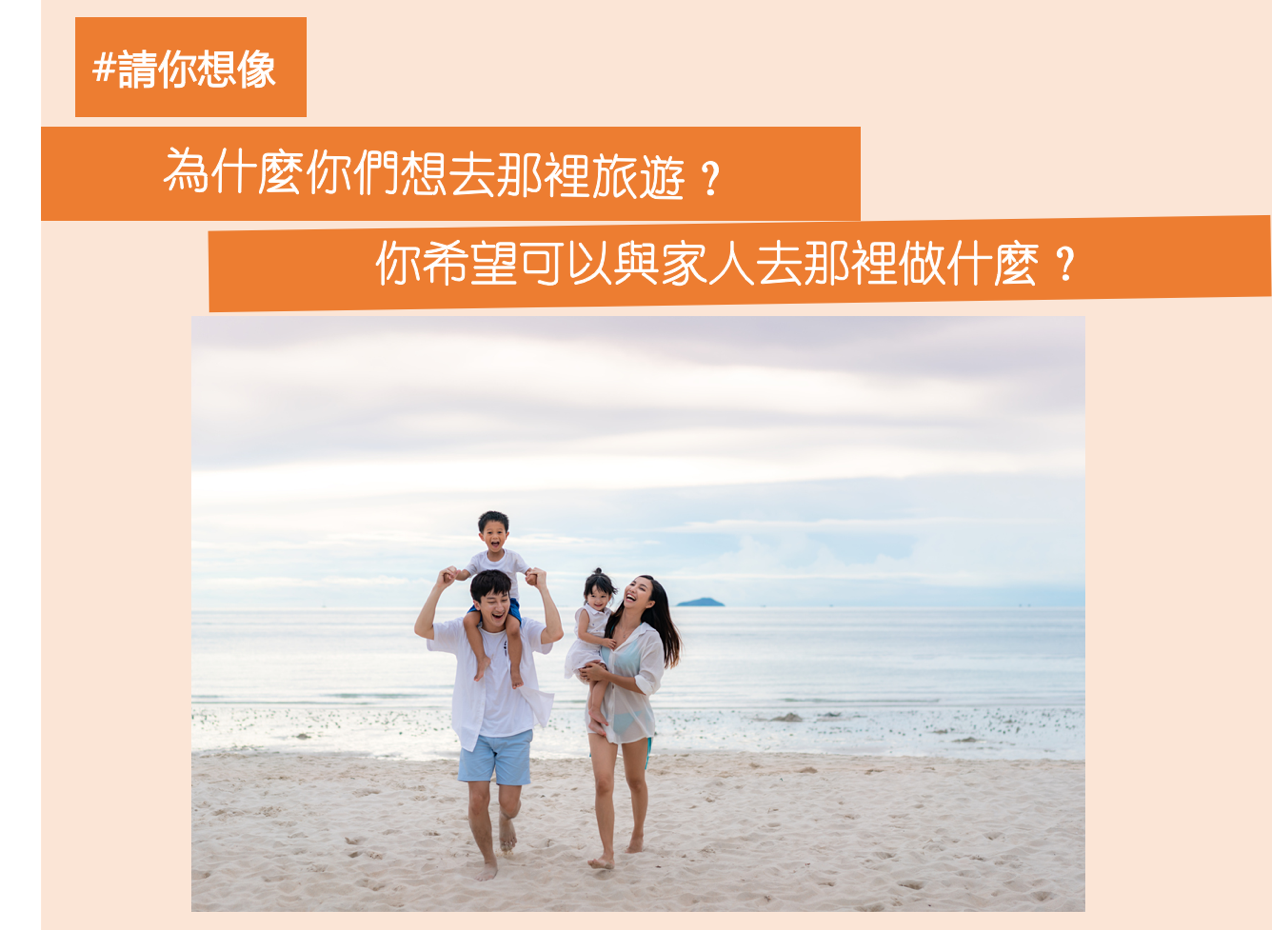


[English]: Why do you want to travel there? What do you want to do with your family during the visit?


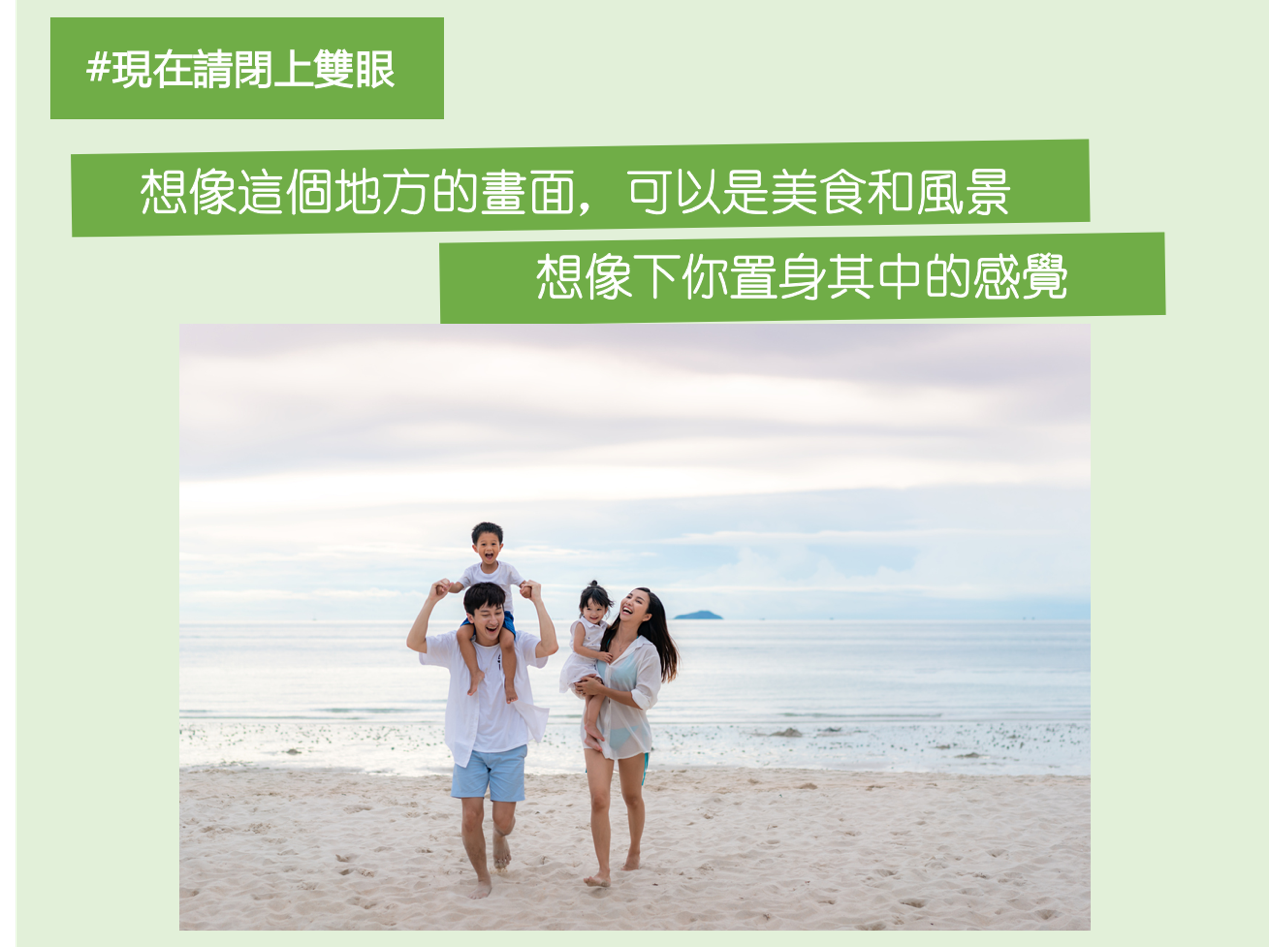


[English]: Now please close your eyes, imagine the sights of this place, including the food and scenery. Imagine how it feels to be there.

[Pause for 5s before the next page bottom appear]


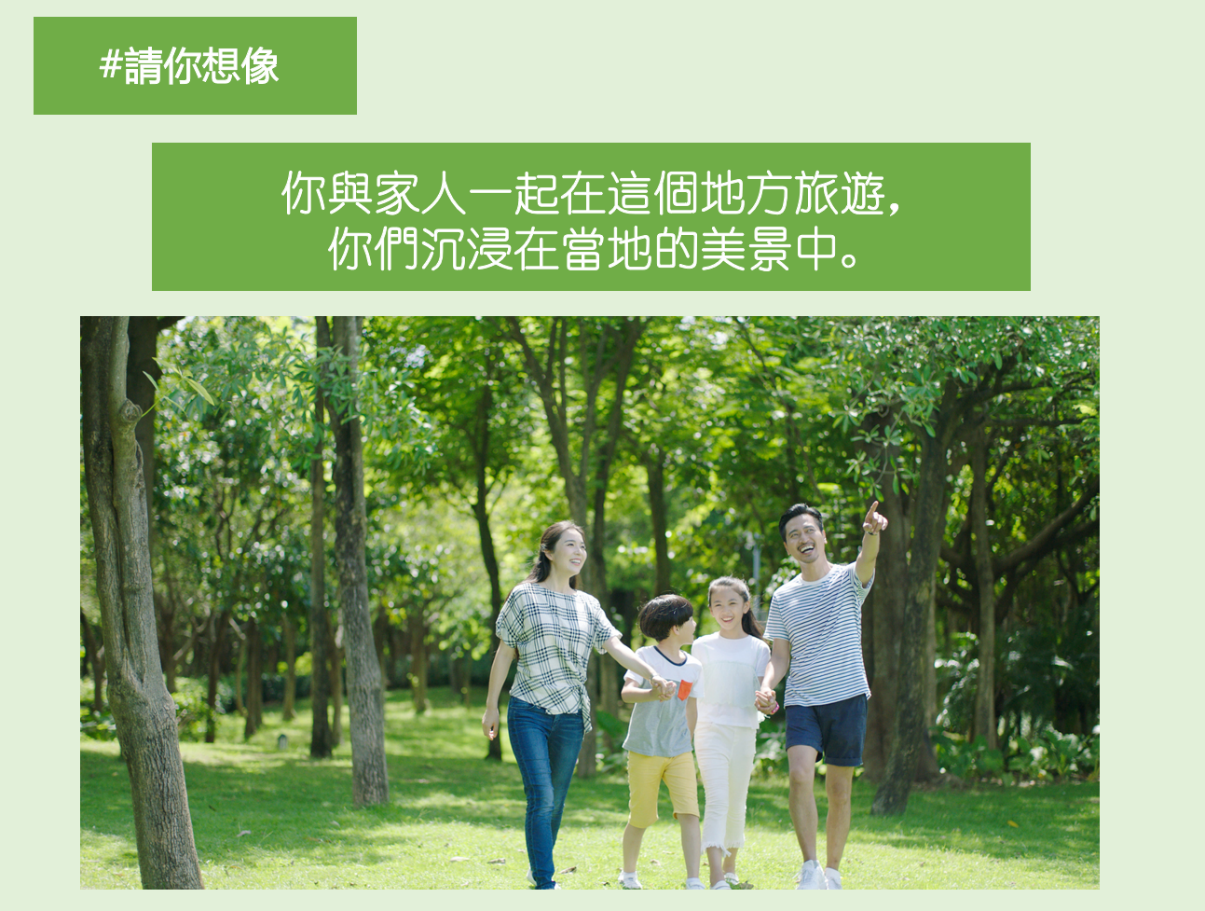


[English]: Please imagine traveling to this place with your family, immersed in the local beauty scenery.

**
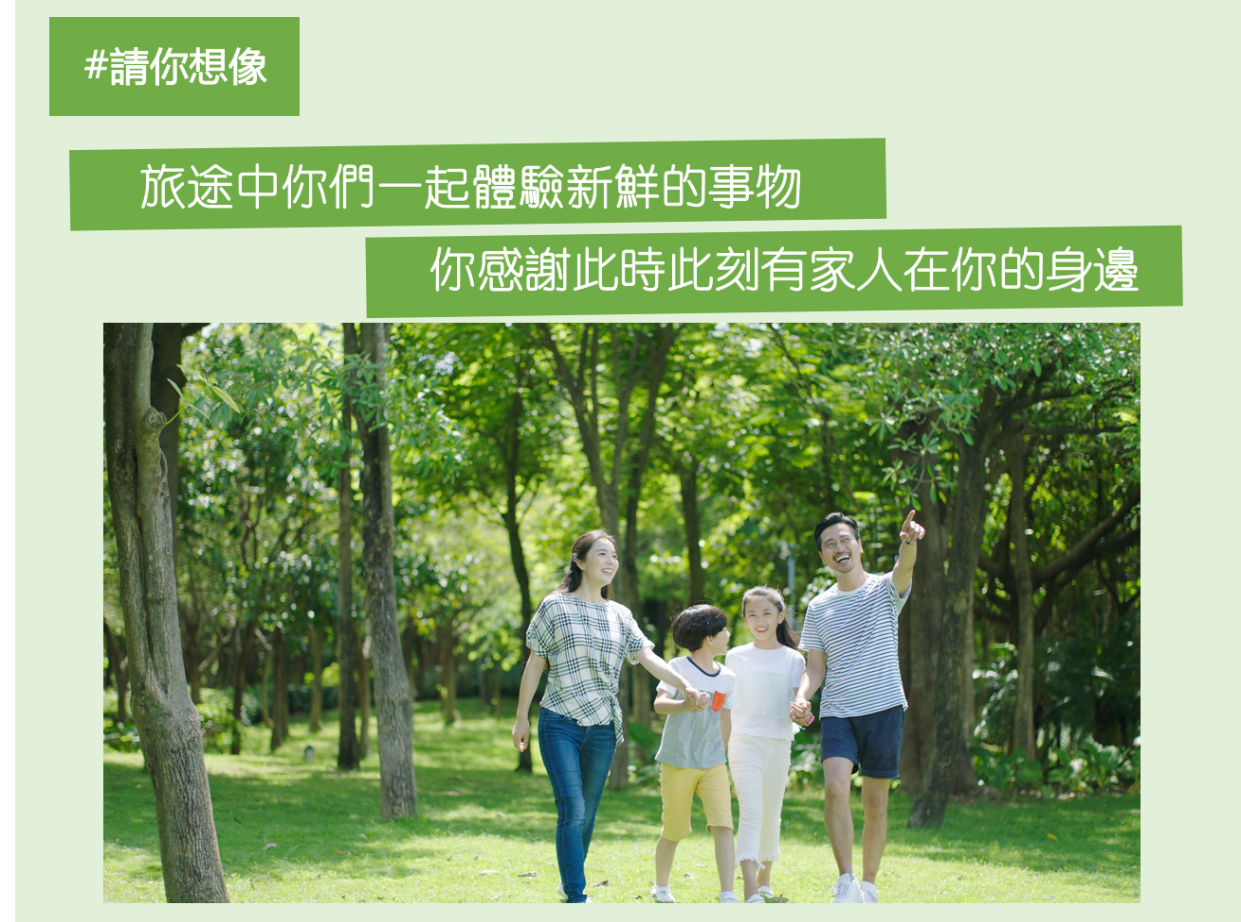
**

[English]: Please imagine experiencing new things together during the trip and feeling grateful to have your family by your side at this moment.

**
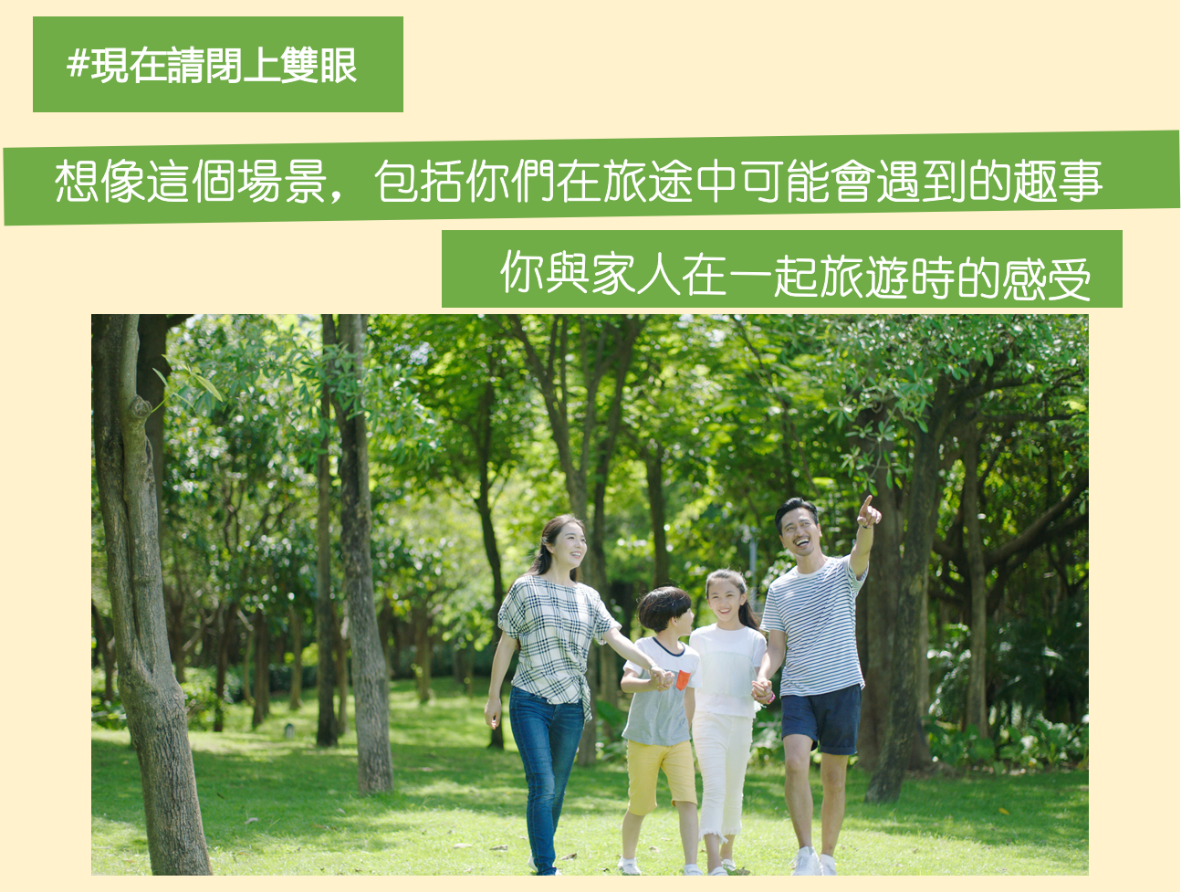
**

[English]: Now please close your eyes, imagine this scene, including the amusing incidents you might encounter on the journey and the feelings you experience traveling with your family.

[Pause for 5s before the next page bottom appear]

**【Next page: Scenario 3】**

**
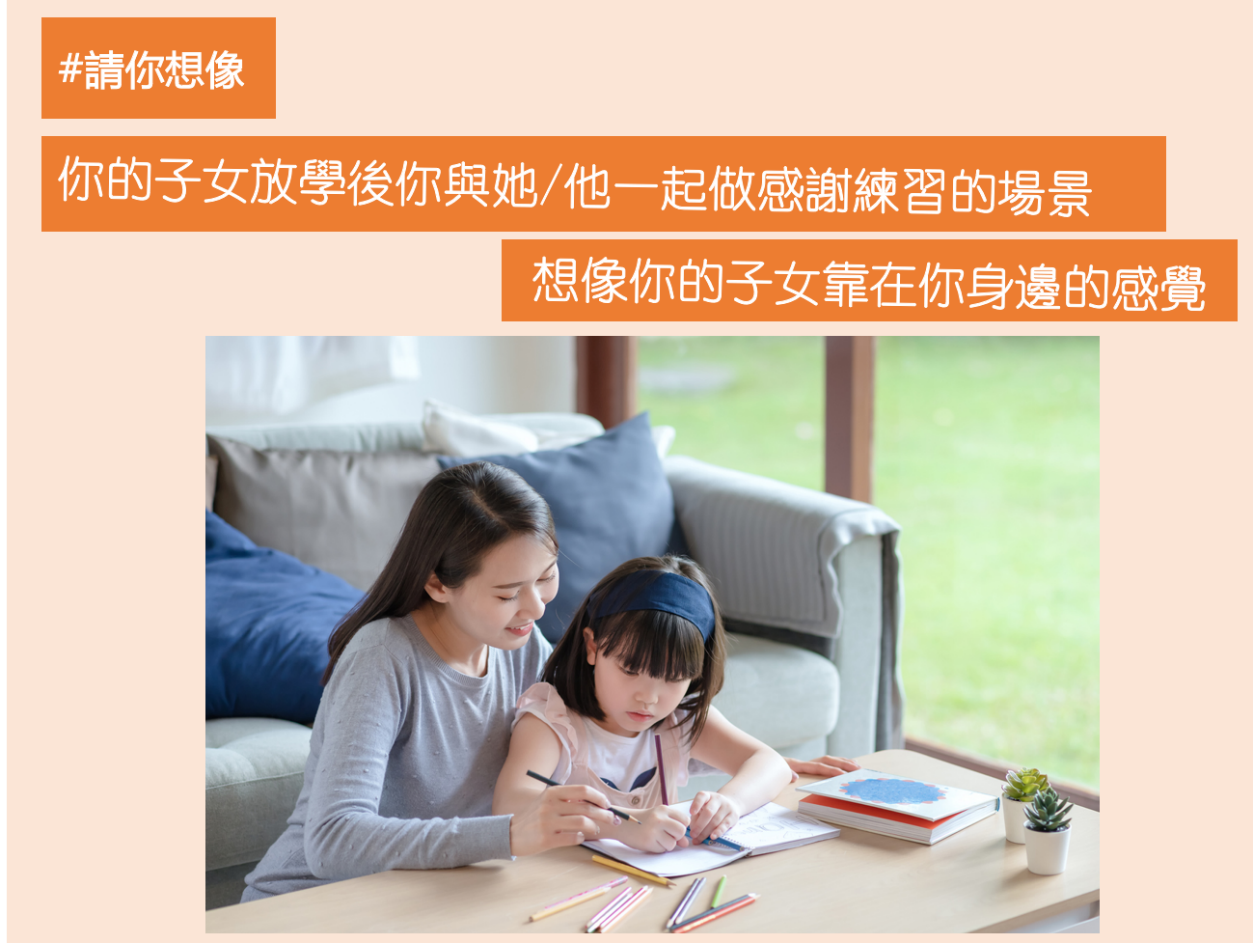
**

[English]: Please imagine the scene of doing a gratitude exercise with your child after school, and the feeling of your child leaning against you.

**
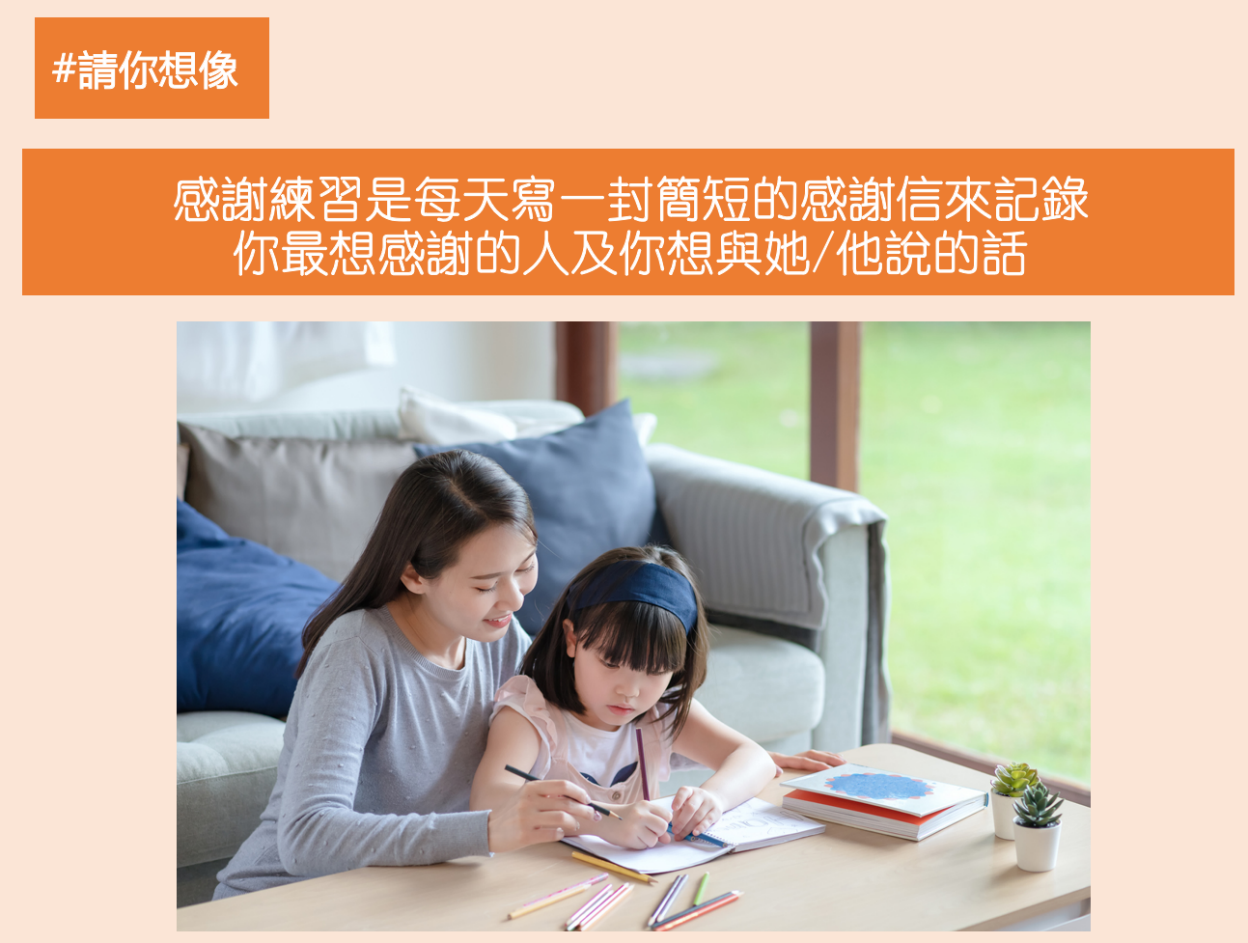
**

[English]: A gratitude exercise involves writing a brief thank-you letter each day to acknowledge the person you are most grateful for and express what you wish to say to them.

**
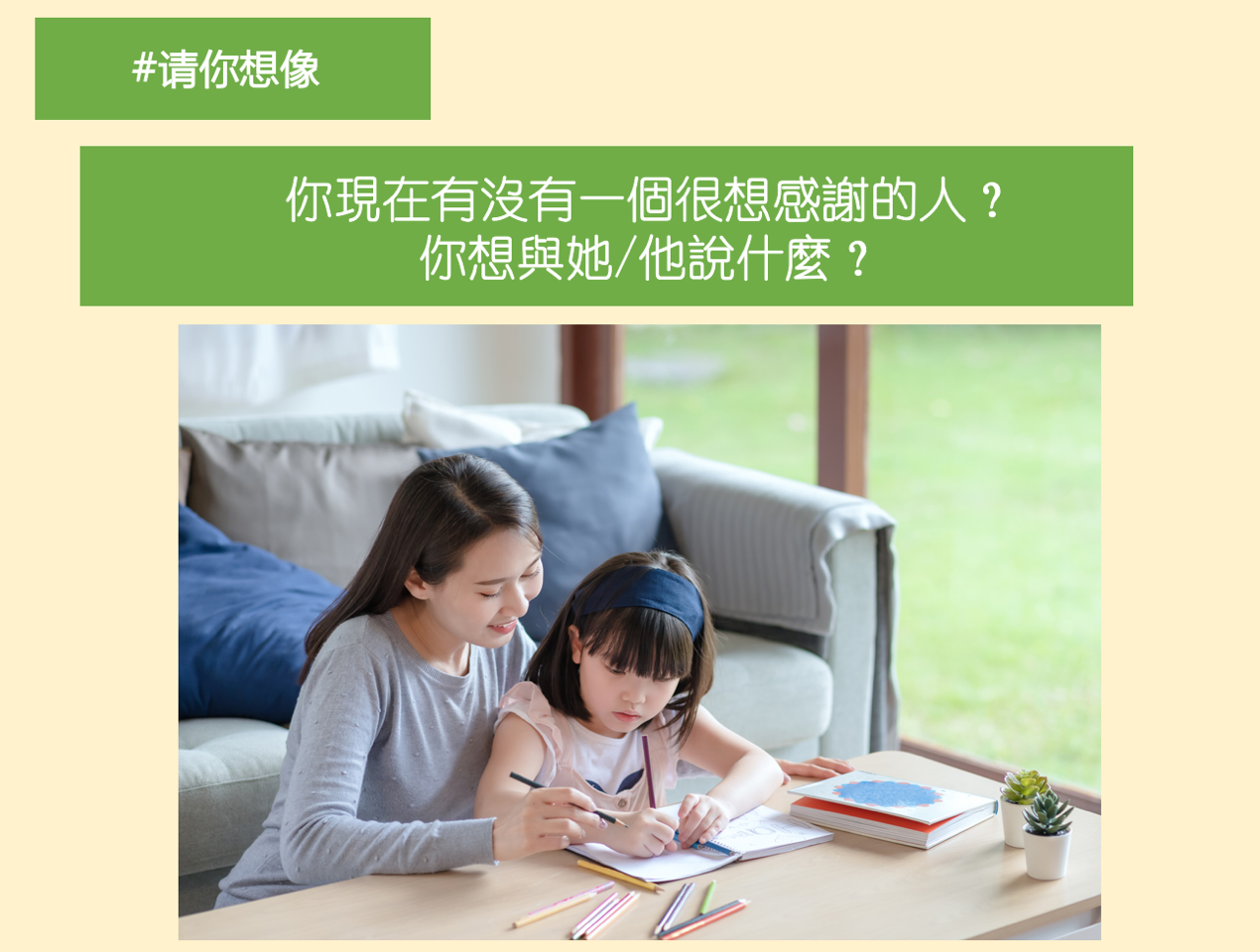
**

[English]: Please imagine someone you are truly grateful for right now. What would you like to say to him/her?

**
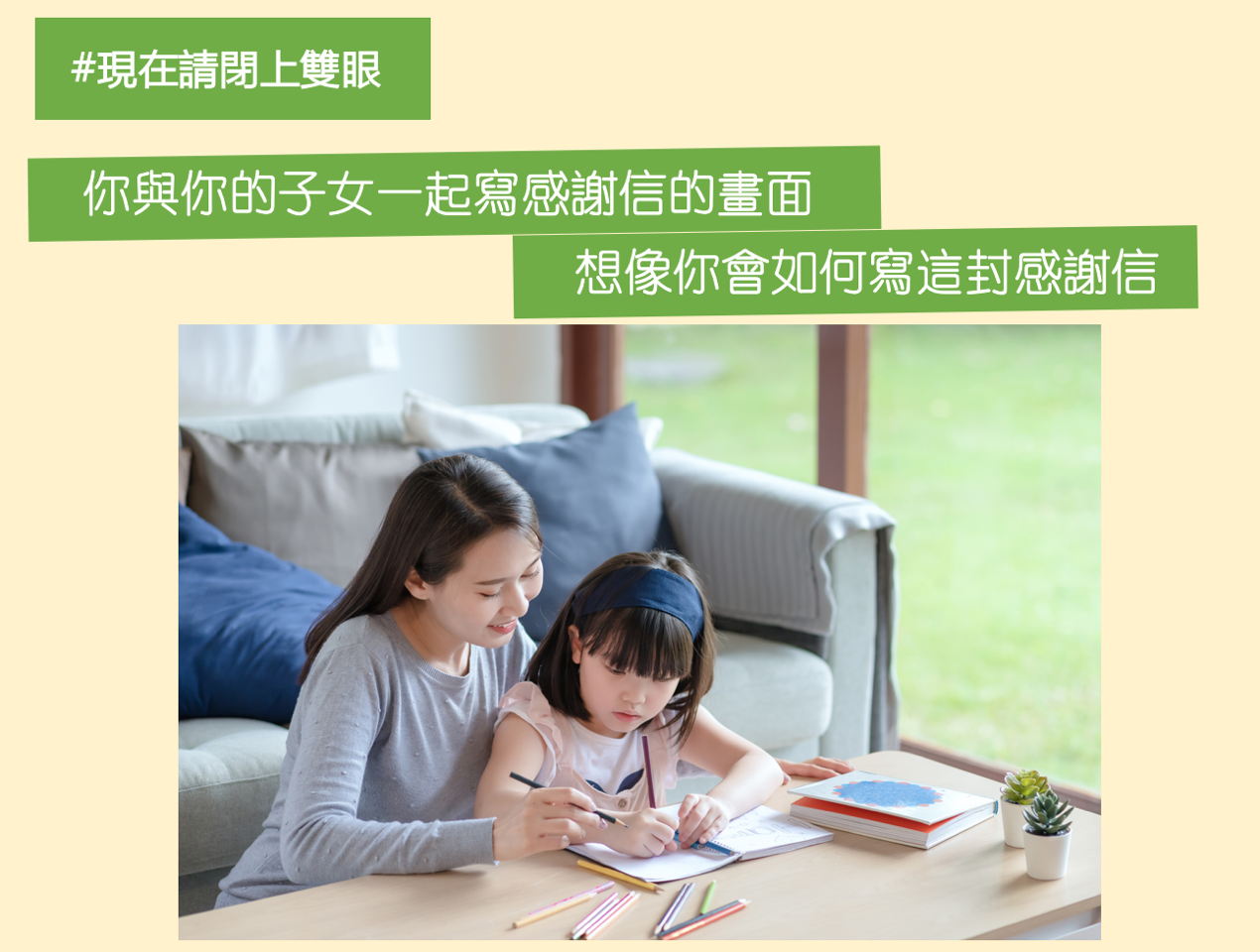
**

[English]: Now please close your eyes and imagine a scene where you are writing a thank-you letter with your child. Imagine how you would write this letter.

[Pause for 5s before the next page bottom appear]

**
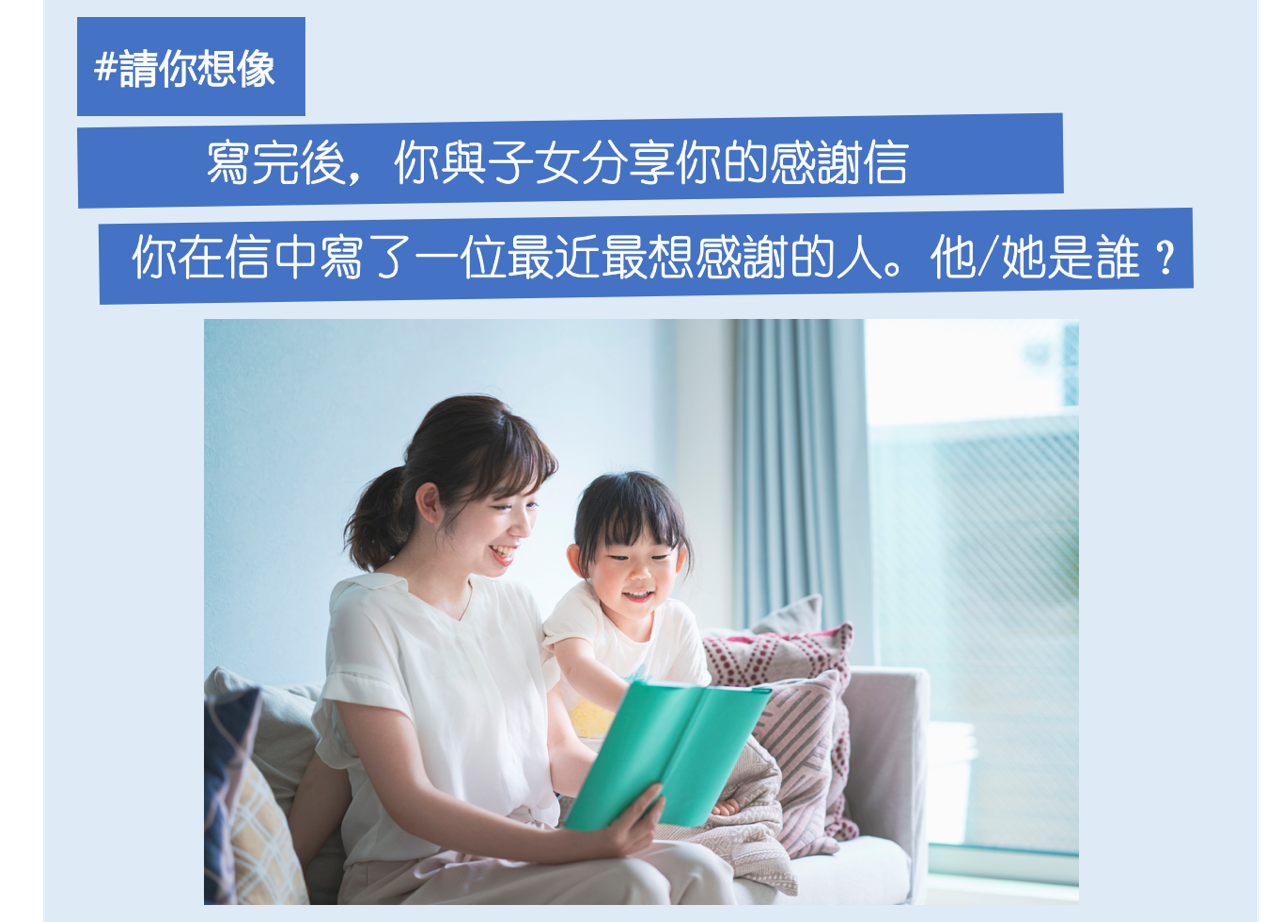
**

[English]: Please imagine sharing your gratitude letter with your child after writing it. In the letter, you write about someone you are most grateful for recently. Who is she/he?

**
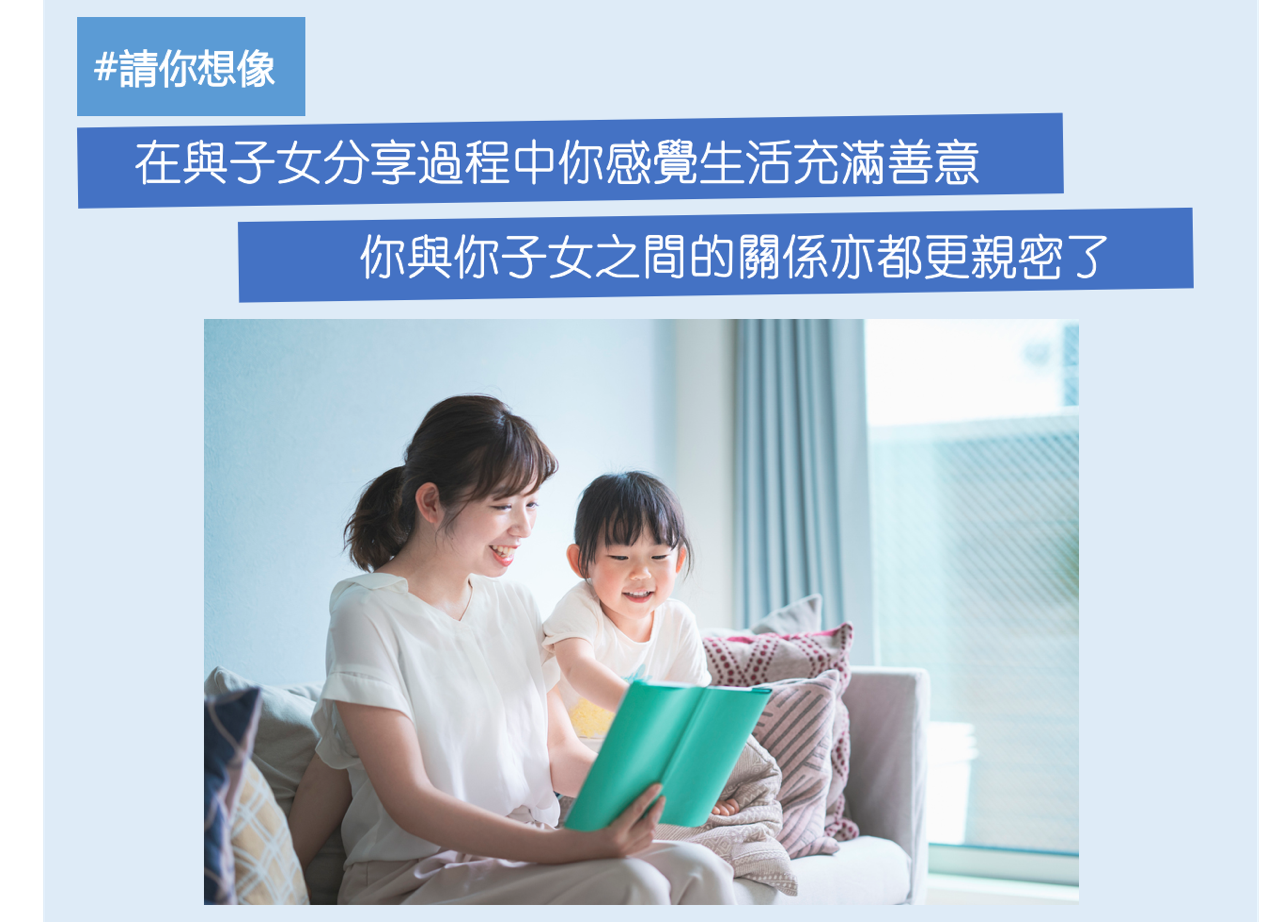
**

[English]: Please imagine that during the process of sharing with your child, you feel life is full of kindness, and your relationship with your child becomes closer.

**
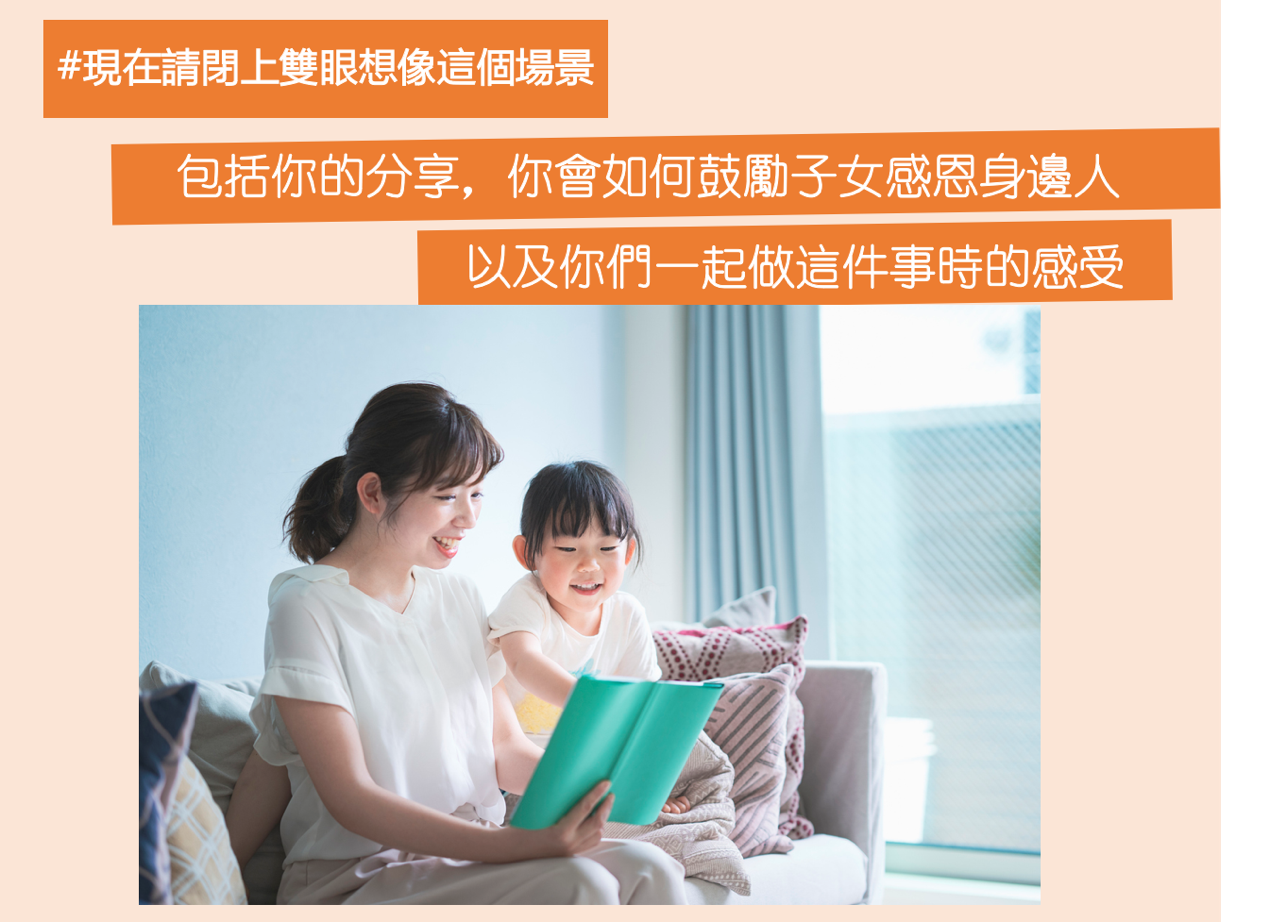
**

[English]: Now please close your eyes and imagine this scene, including your sharing, how you encourage your child to appreciate those around them, and the feelings you both experience while doing this together?

[Pause for 5s before the next page bottom appear]

### **PIS Male Version**

**[Next page: Scenario 1]**


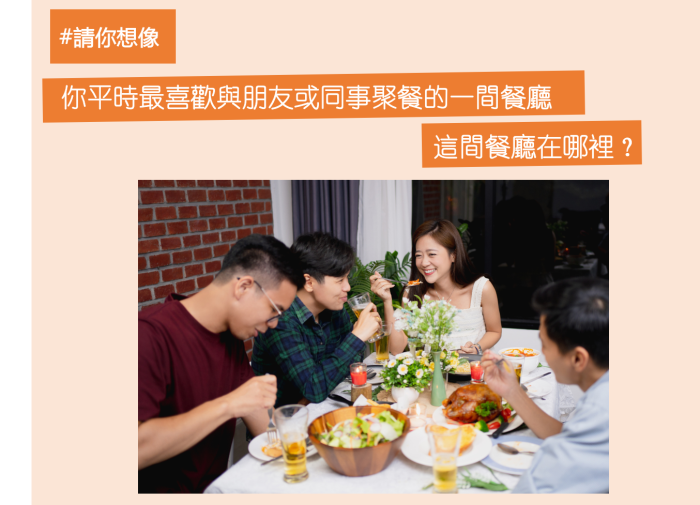


[English]: Please imagine your favorite restaurant where you usually enjoy dining with friends or colleagues. Where is this restaurant located?


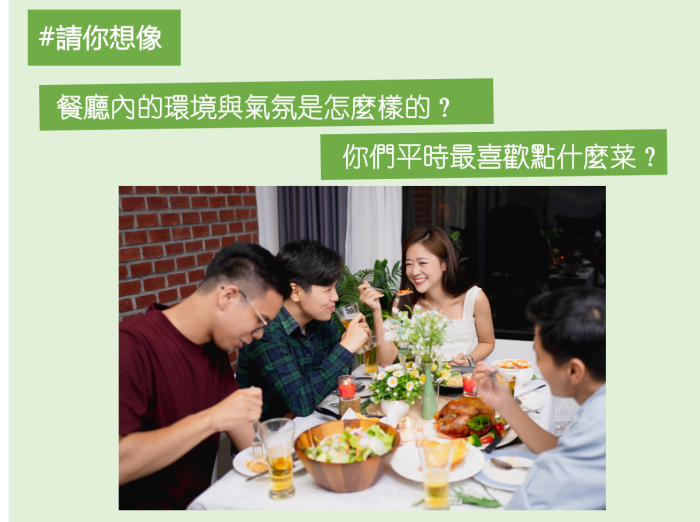


[English]: Please imagine the ambiance and atmosphere inside the restaurant. What dishes do you usually enjoy ordering?


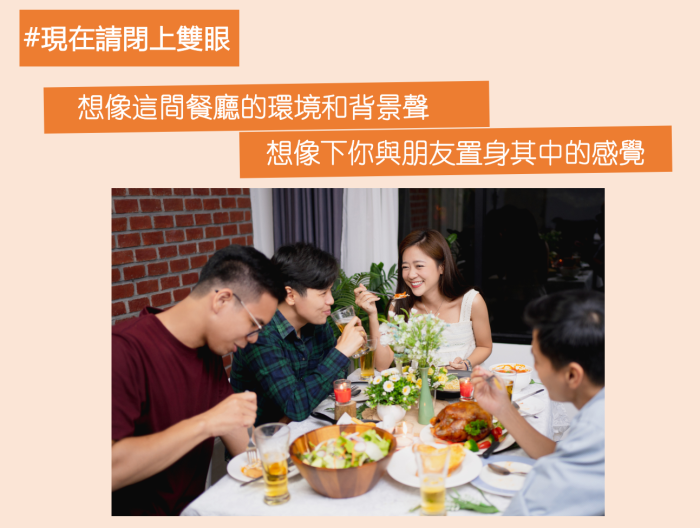


[English]: Now please close your eyes, imagine the environment and background sounds of this restaurant, and the feeling of being there with your friends.

[Pause for 5s before the next page bottom appear]


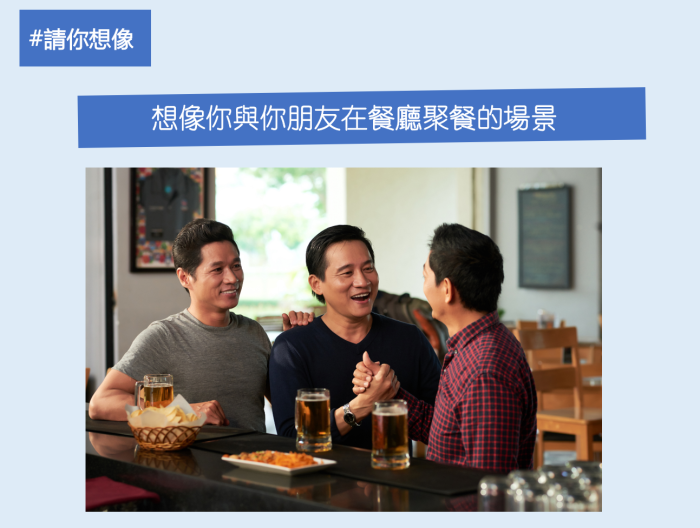


[English]: Now please imagine the scene of dining with your friends at this restaurant.


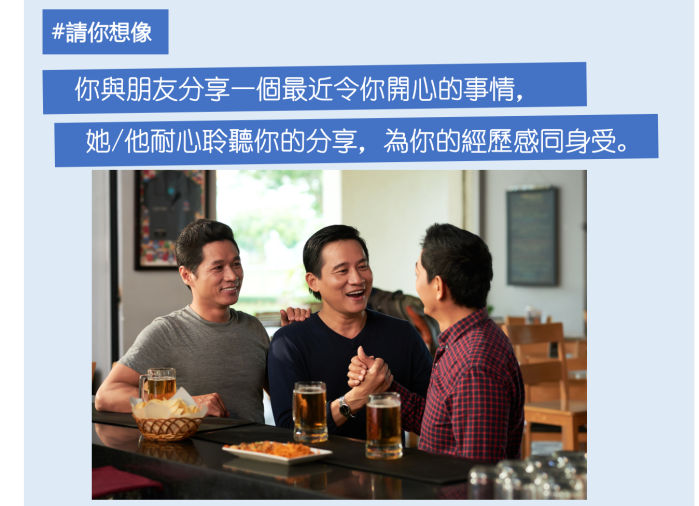


[English]: Continue to imagine sharing a recent happy event with your friend. He/she listens patiently to your story and empathizes with your experience.


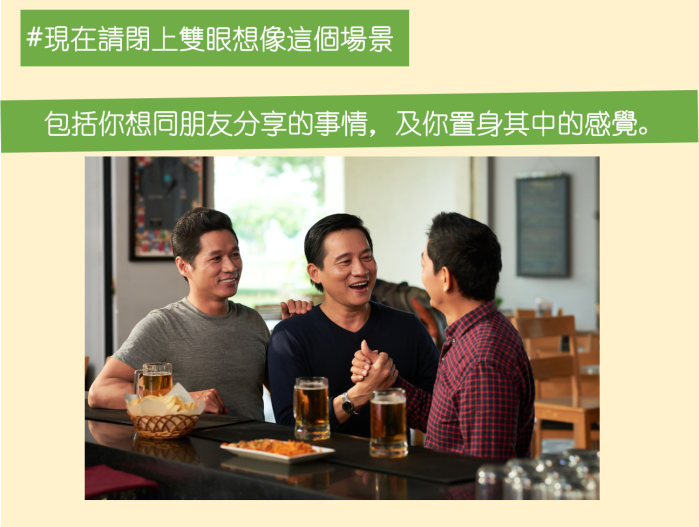


[English]: Now please close your eyes. Imagine this scenario and how you feel about it.

[Pause for 5s before the next page bottom appear]

**[Next page: Scenario 2]**


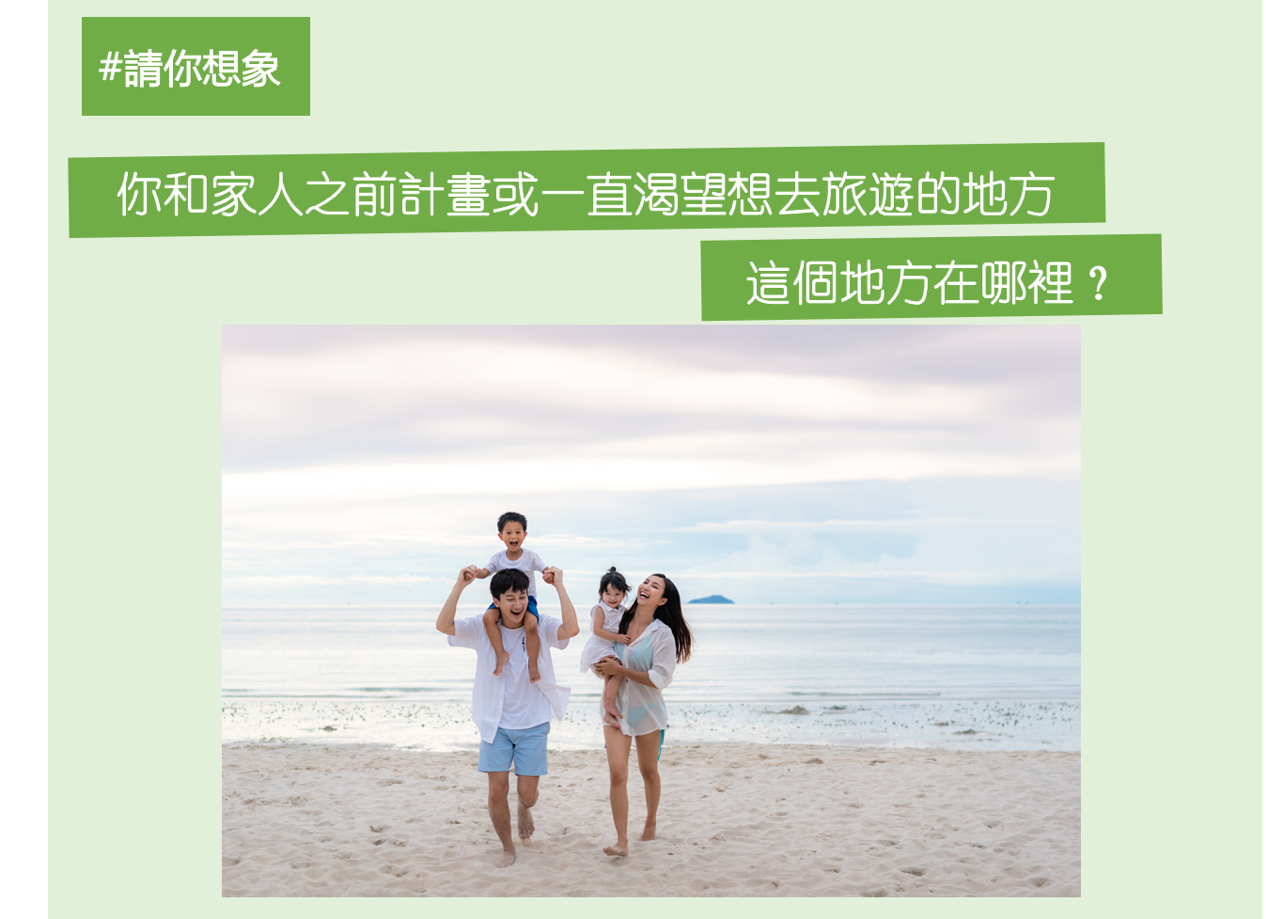


[English]: Please imagine a place you and your family have planned or always wanted to visit. Where is this place?


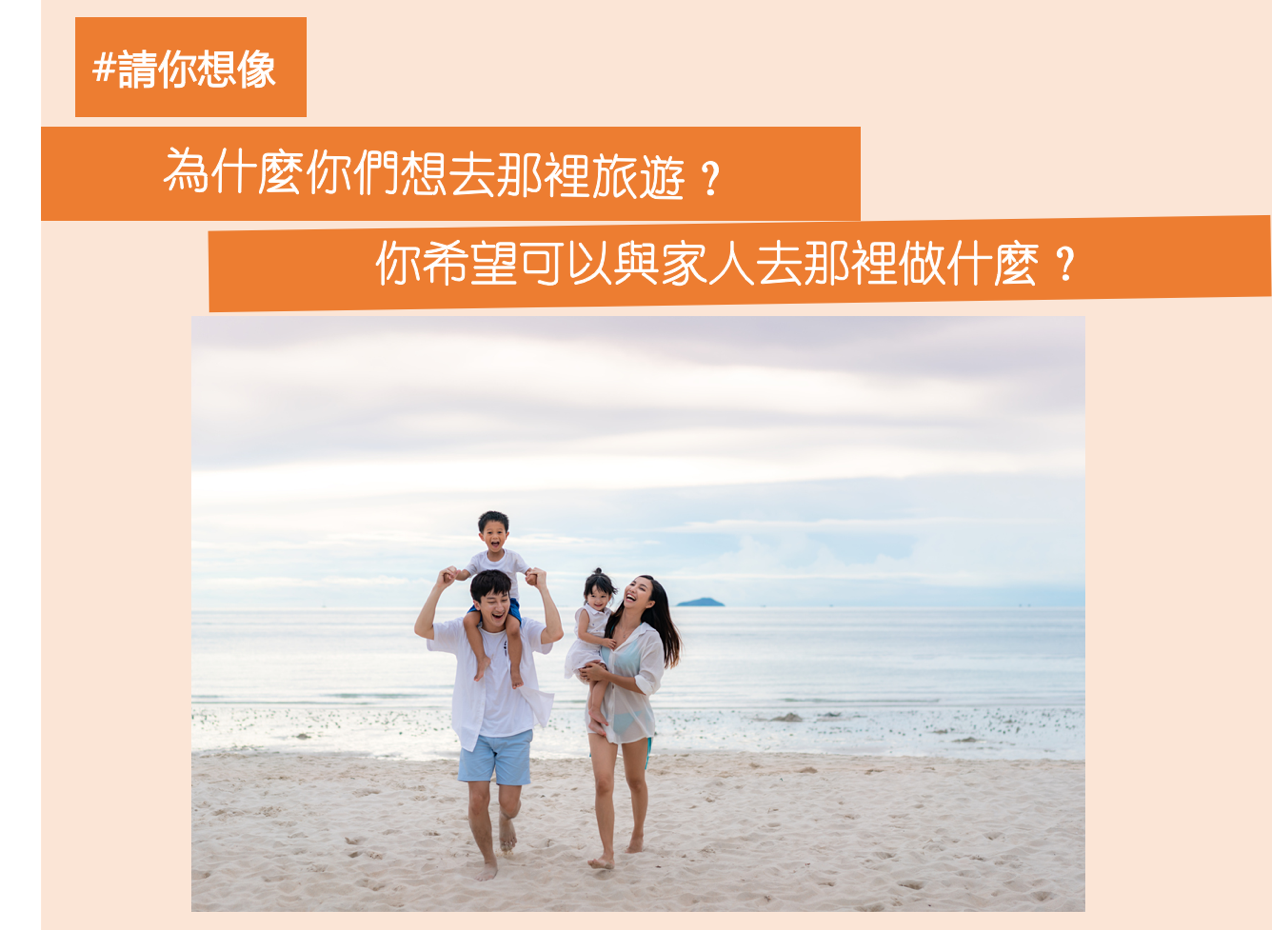


[English]: Why do you want to travel there? What do you want to do with your family during the visit?


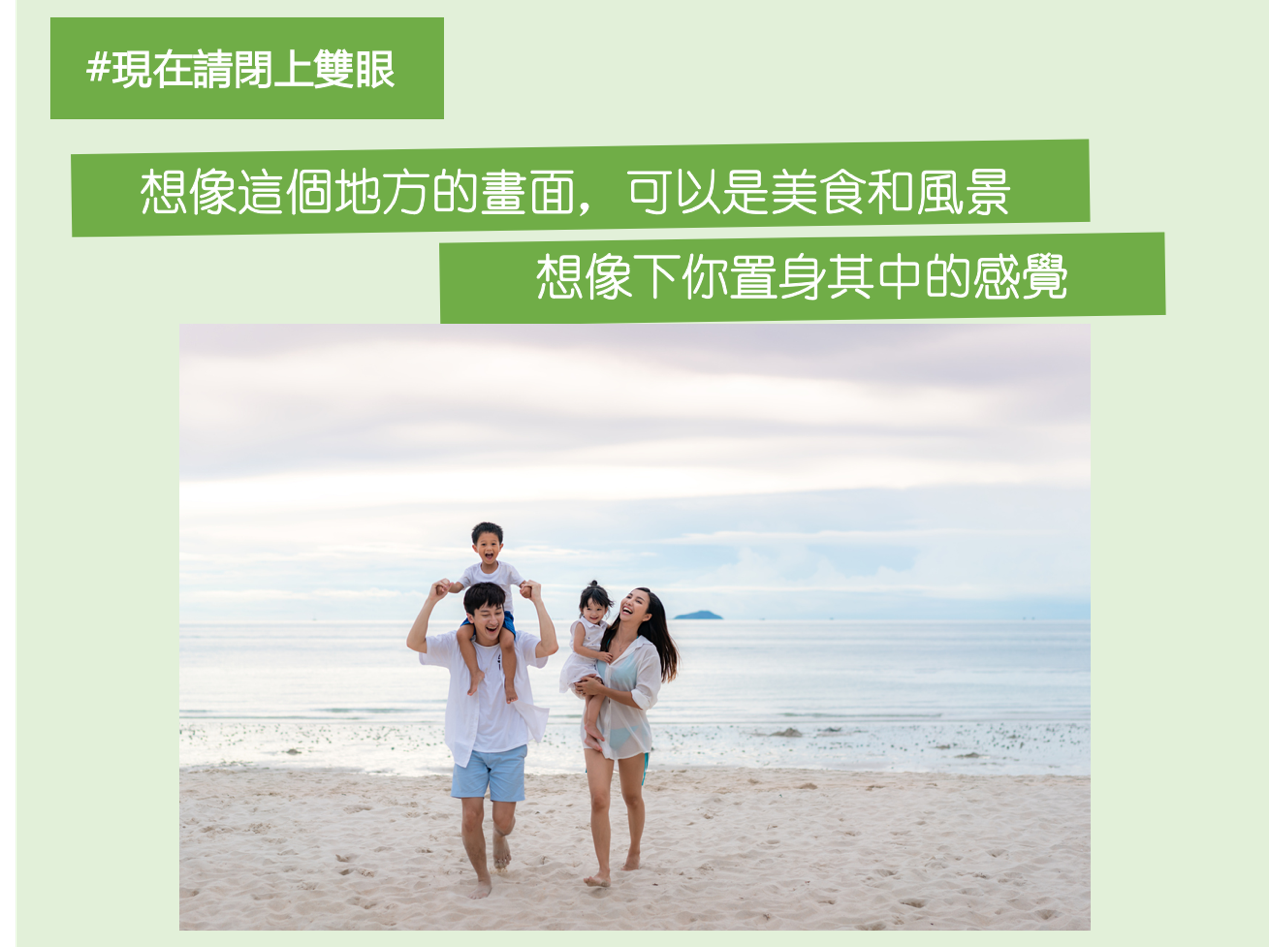


[English]: Now please close your eyes, imagine the sights of this place, including the food and scenery. Imagine how it feels to be there.

[Pause for 5s before the next page bottom appear]


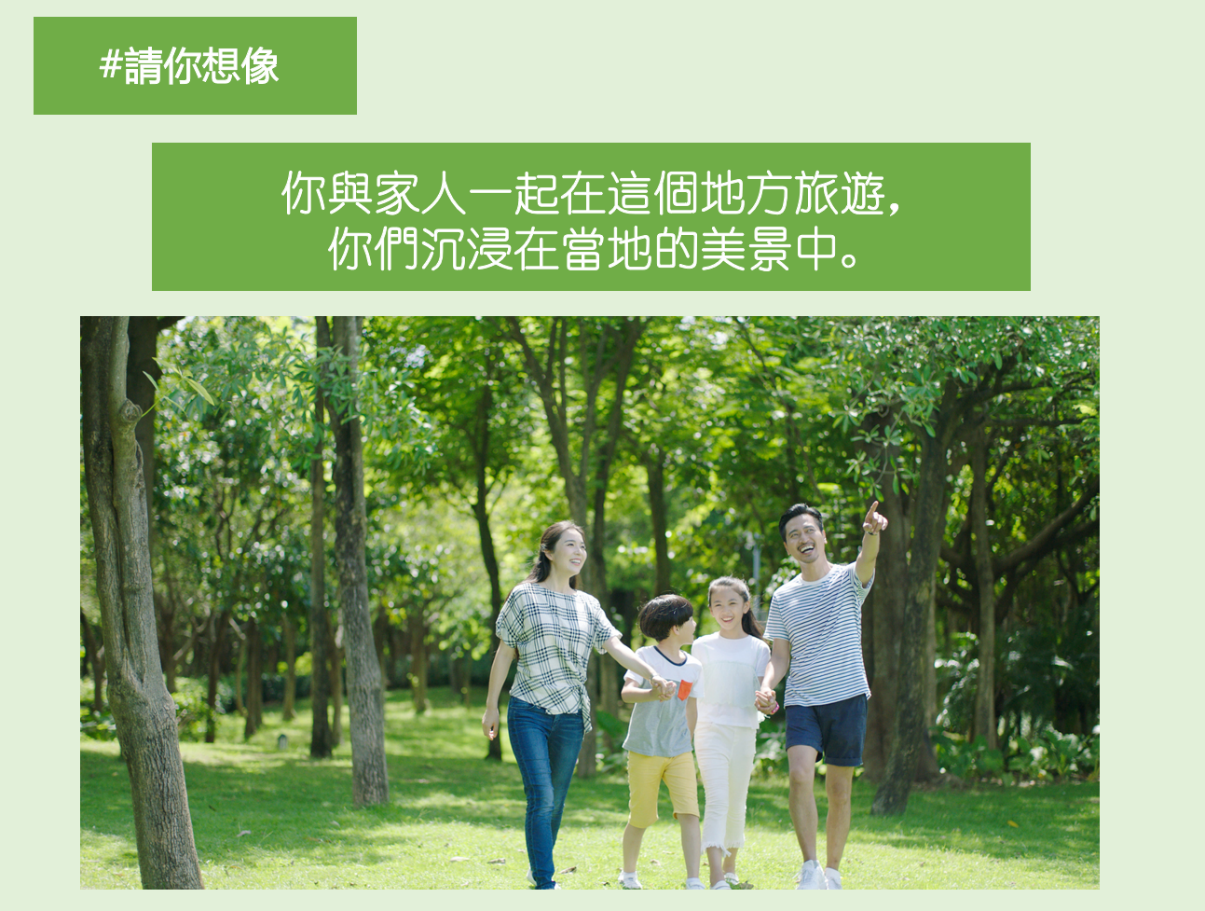


[English]: Please imagine traveling to this place with your family, immersed in the local beauty scenery.

**
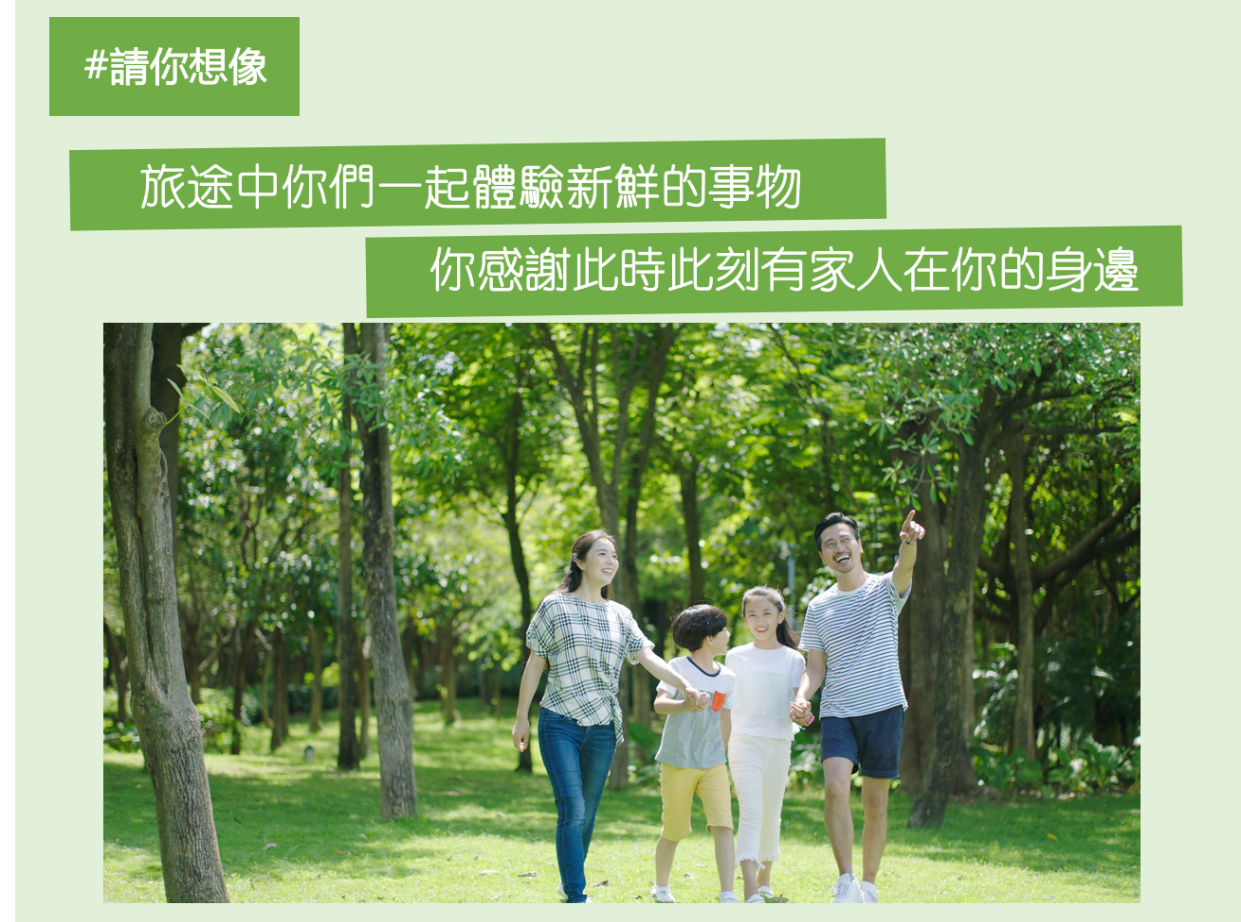
**

[English]: Please imagine experiencing new things together during the trip and feeling grateful to have your family by your side at this moment.

**
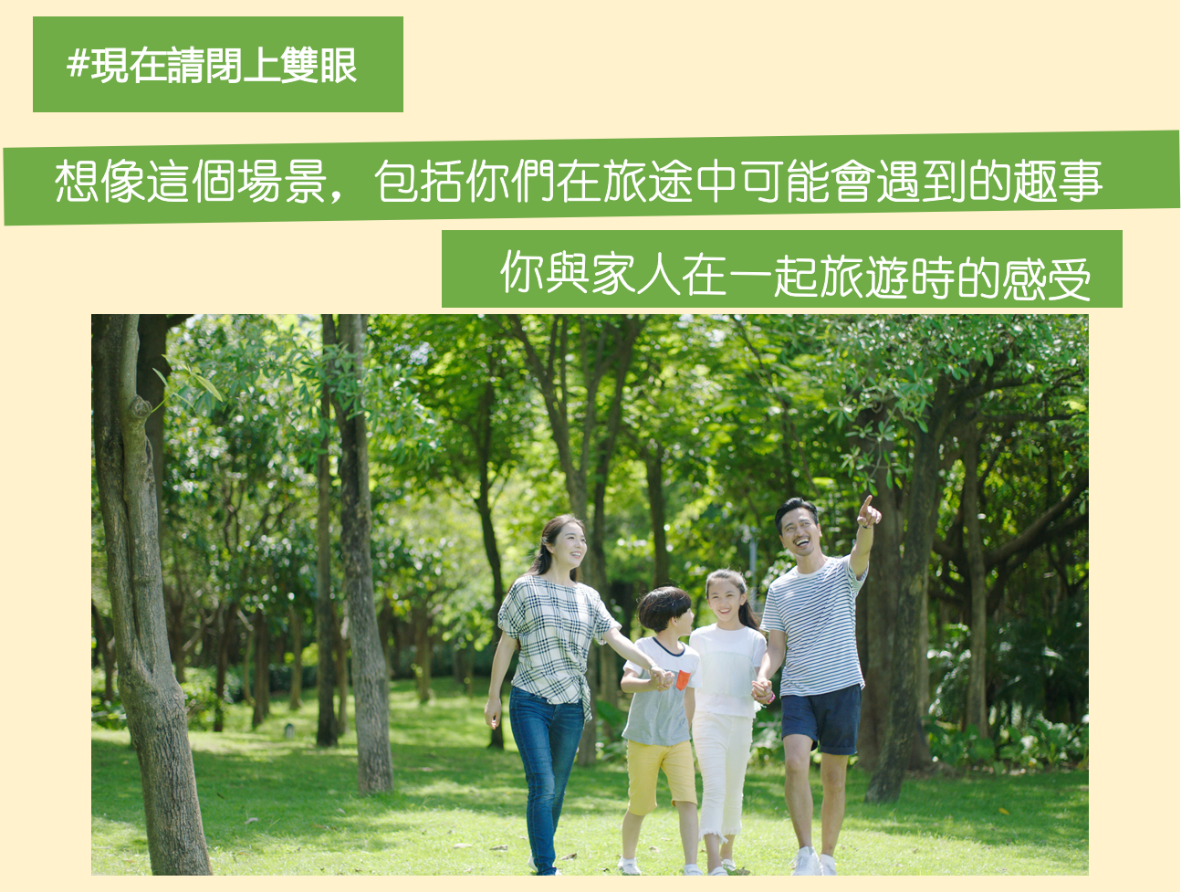
**

[English]: Now please close your eyes, imagine this scene, including the amusing incidents you might encounter on the journey and the feelings you experience traveling with your family.

[Pause for 5s before the next page bottom appear]

**【Next page: Scenario 3】**


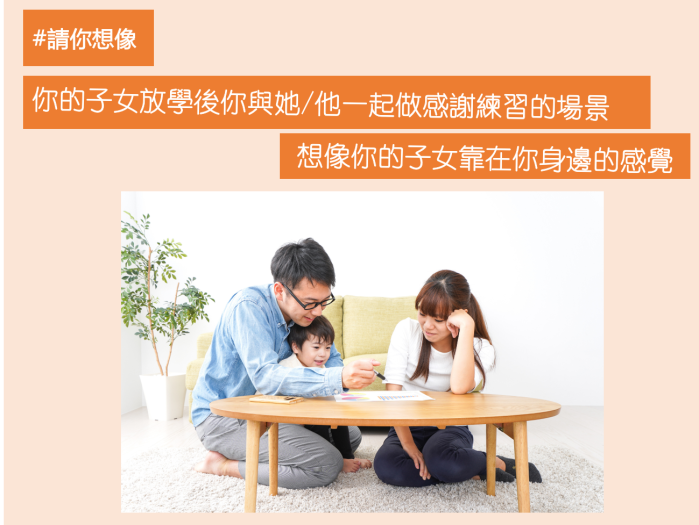


[English]: Please imagine the scene of doing a gratitude exercise with your child after school, and the feeling of your child leaning against you.


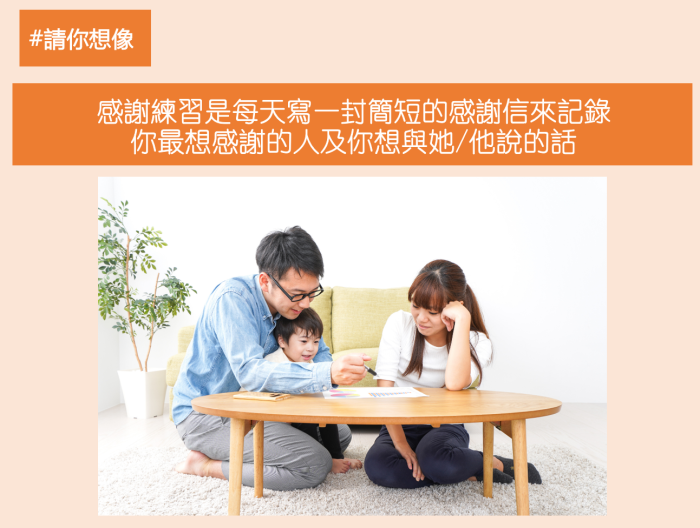


[English]: A gratitude exercise involves writing a brief thank-you letter each day to acknowledge the person you are most grateful for and express what you wish to say to them.


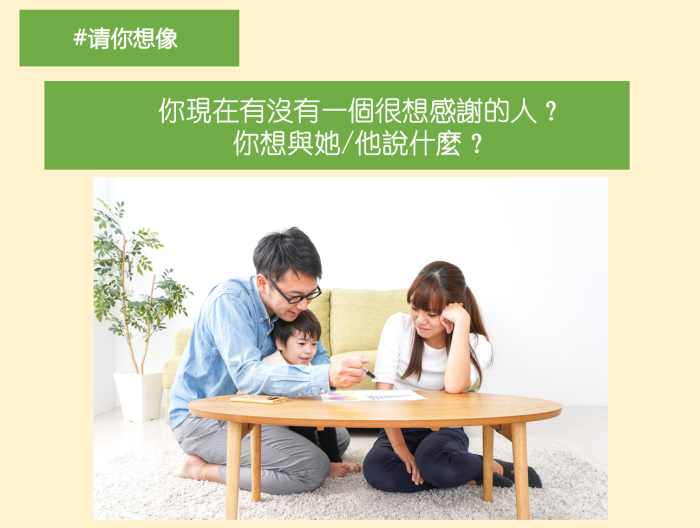


[English]: Please imagine someone you are truly grateful for right now. What would you like to say to him/her?


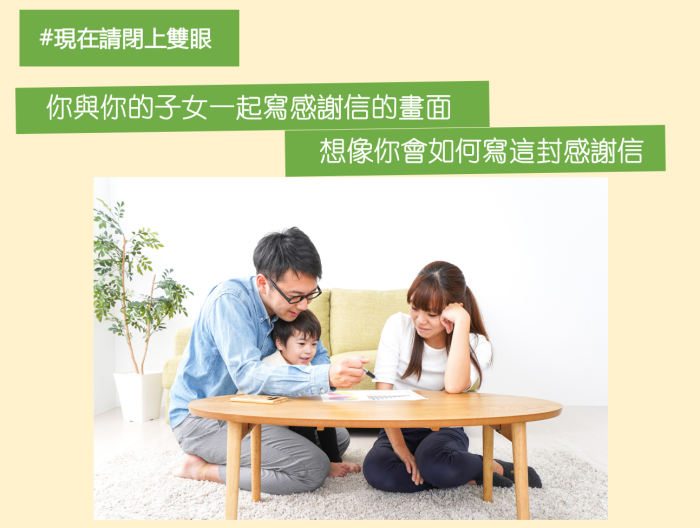


[English]: Now please close your eyes and imagine a scene where you are writing a thank-you letter with your child. Imagine how you would write this letter.

[Pause for 5s before the next page bottom appear]


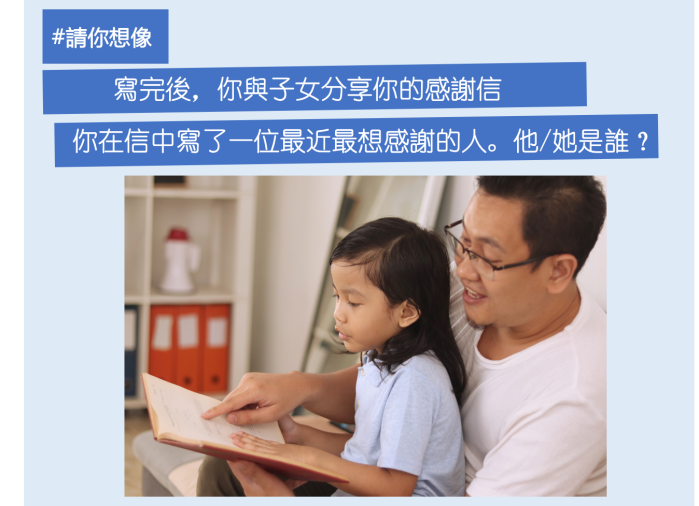


[English]: Please imagine sharing your gratitude letter with your child after writing it. In the letter, you write about someone you are most grateful for recently. Who is she/he?


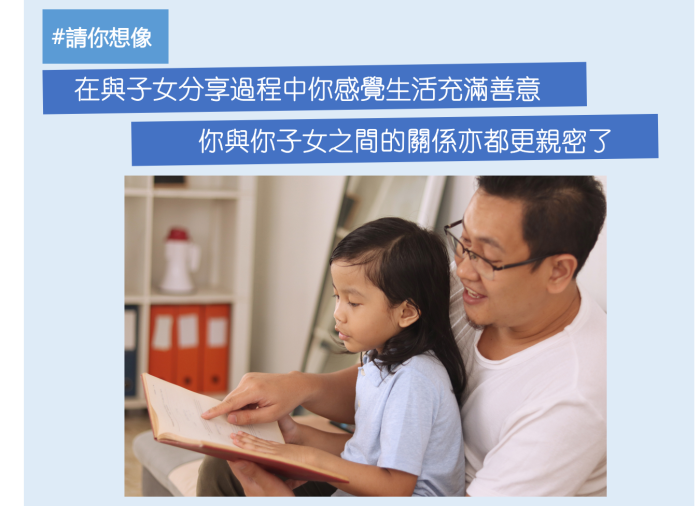


[English]: Please imagine that during the process of sharing with your child, you feel life is full of kindness, and your relationship with your child becomes closer.


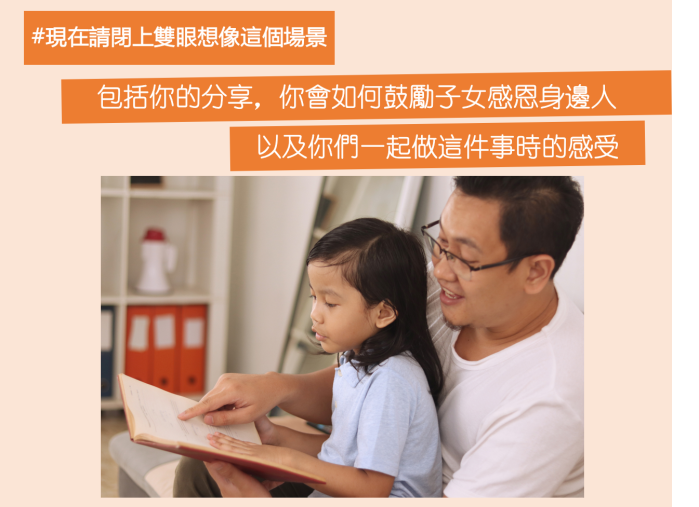


[English]: Now please close your eyes and imagine this scene, including your sharing, how you encourage your child to appreciate those around them, and the feelings you both experience while doing this together?

[Pause for 5s before the next page bottom appear]

## Neutral Recall Simulation (PIS) Materials

Participants assigned to the PIS group received the following instructions:

Below is an interactive task designed to test your memorization ability. In the following pages, you will be asked to recall certain life scenarios that occurred in the past week. To ensure you have sufficient time to complete the recall tasks, we will provide tips on each page to aid your memorization. Please complete the task at your own pace.


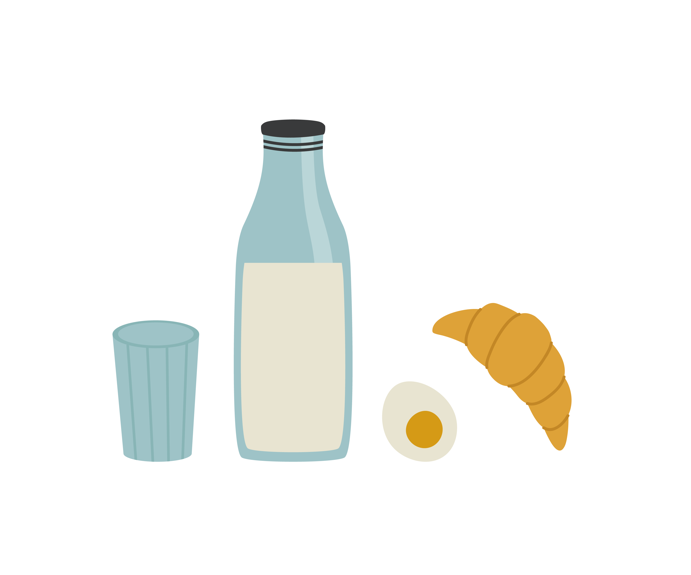


[English]: First, please recall what you had for breakfast yesterday. Where did you have your breakfast?


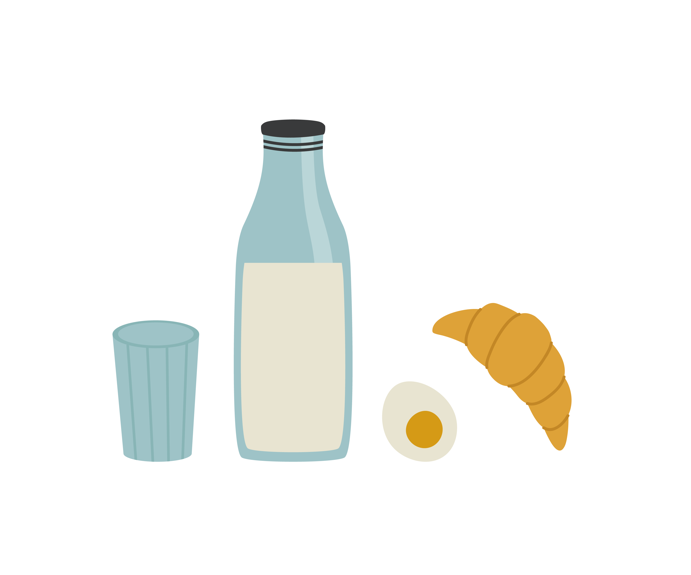


[English]: Please recall what you ate, and describe the shape and taste of the food.


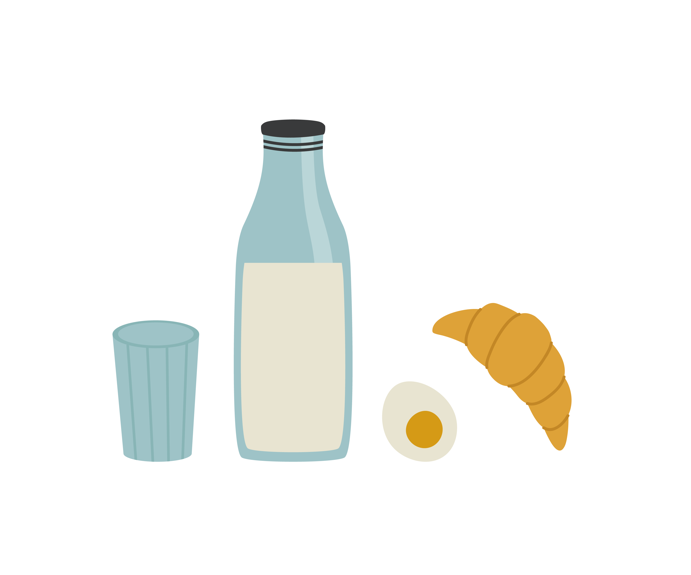


[English]: Now please close your eyes and recall all the details of yesterday’s breakfast. Focus specifically on memorizing the food itself.

[Pause for 5s before the next page bottom appear]


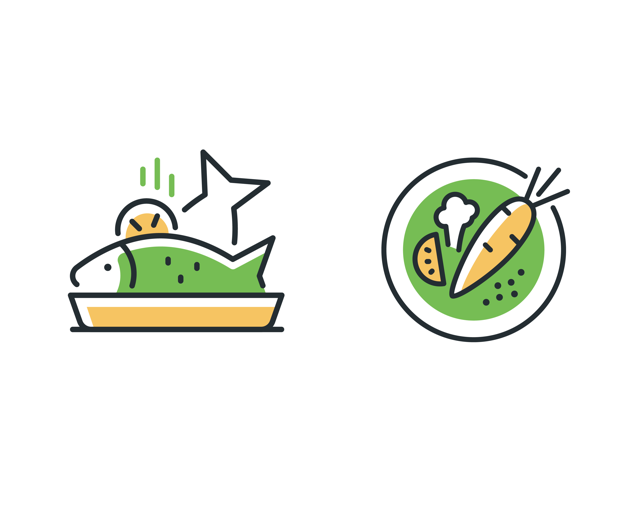


[English]: Next, please recall what you had for lunch yesterday. Where did you have your lunch?


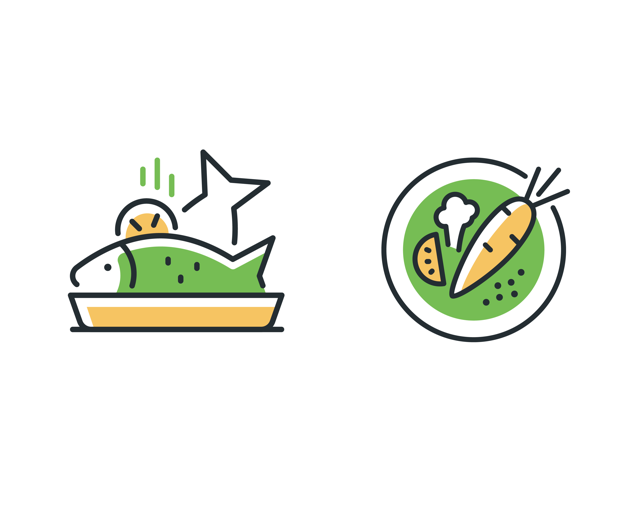


[English]: Please recall what you ate, and describe the shape and taste of the food.


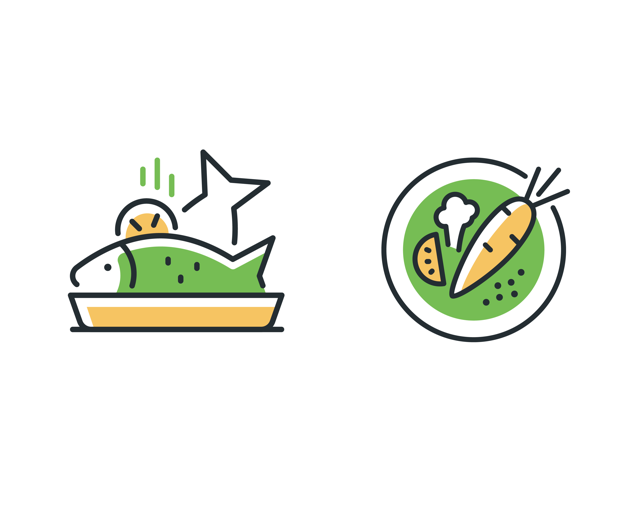


[English]: Now please close your eyes and recall all the details of yesterday’s lunch. Focus specifically on memorizing the food itself.

[Pause for 5s before the next page bottom appear]


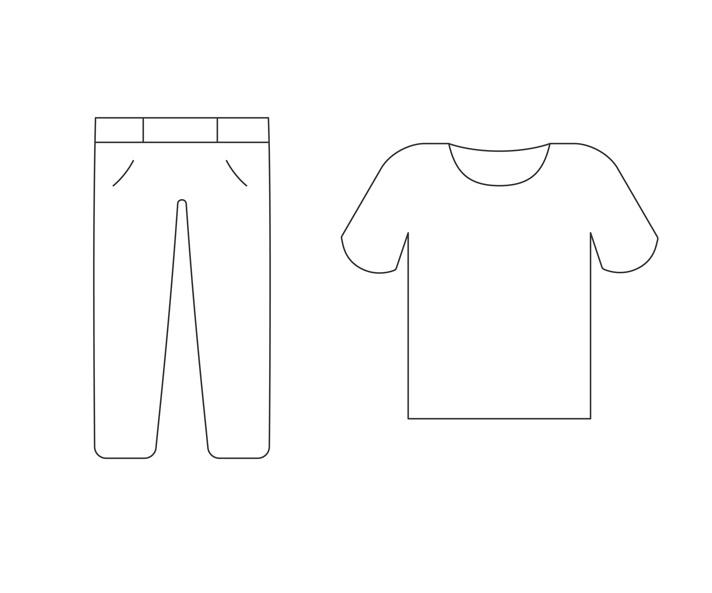


[English]: Please recall what clothes you wore yesterday.


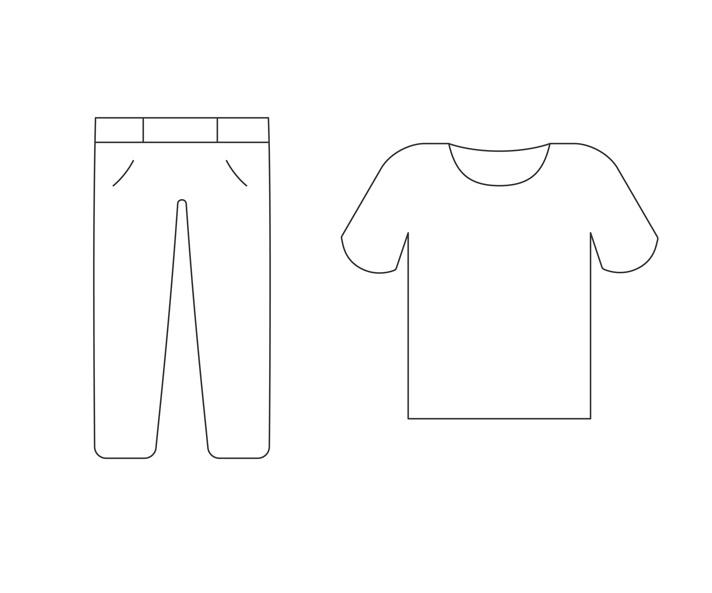


[English]: Please recall the material, color, and style of the clothing you wore yesterday.


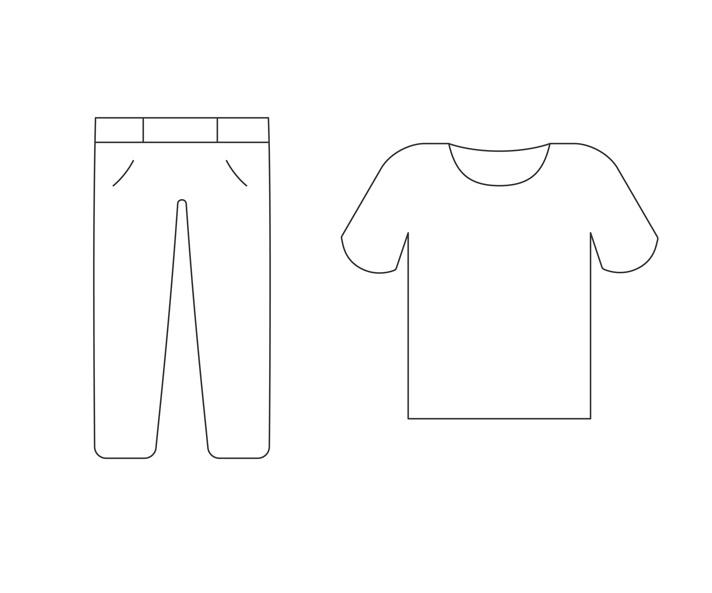


[English]: Now please close your eyes and recall all the details of yesterday’s clothing. Focus specifically on memorizing the closing itself.

[Pause for 5s before the next page bottom appear]


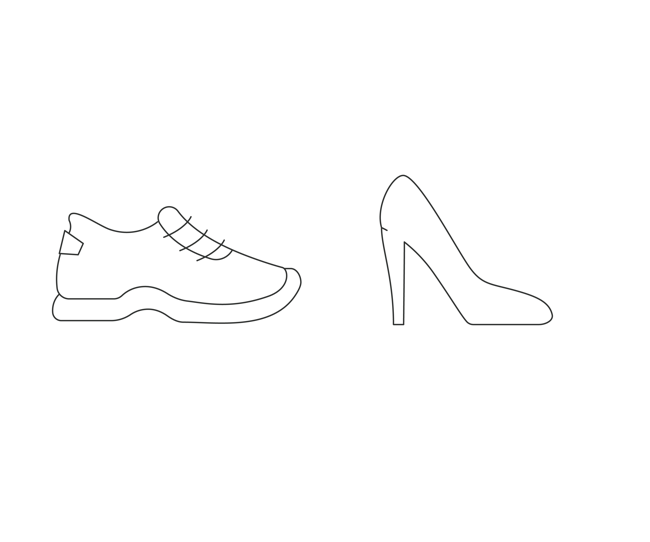


[English]: Next, please recall which shoes you have worn most frequently when going out in the past week. Do you remember the brand of the shoes?


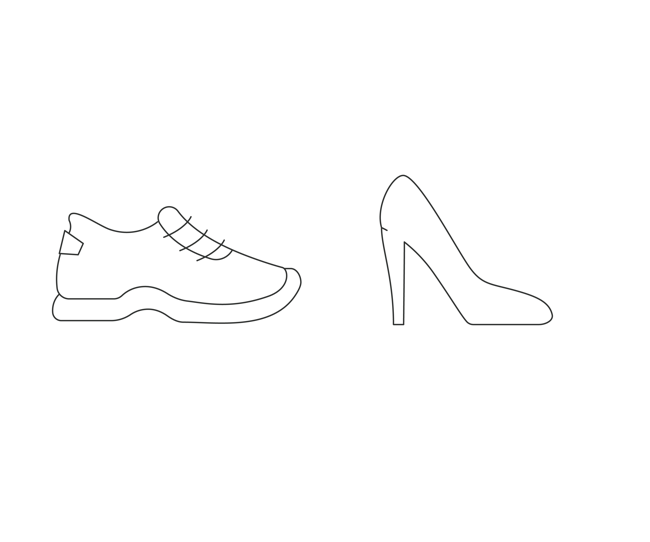


[English]: Please recall the material, color, and style of the shoes you wore.


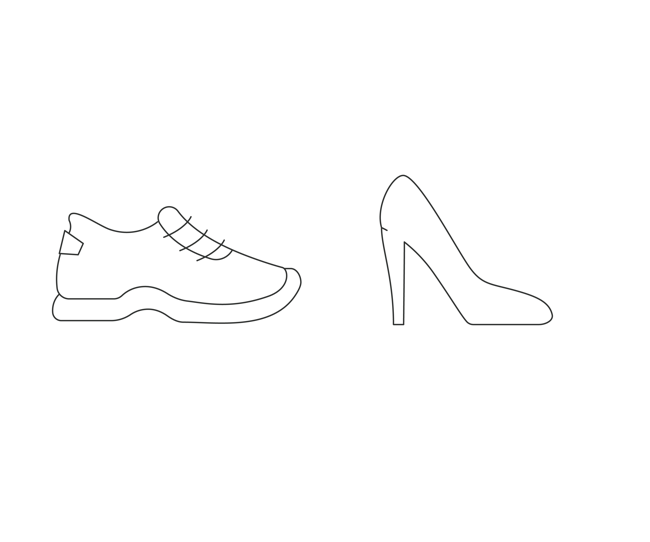


[English]: Now please close your eyes and recall all the details of the shoes you ware. Focus specifically on memorizing the shoes itself.

[Pause for 5s before the next page bottom appear]


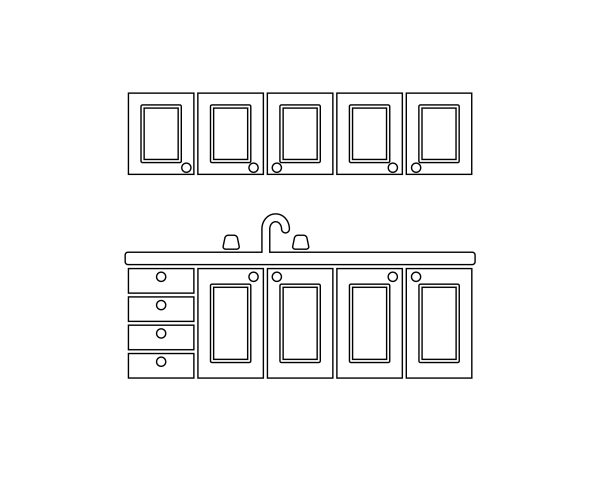


[English]: Please recall how your kitchen looks like.


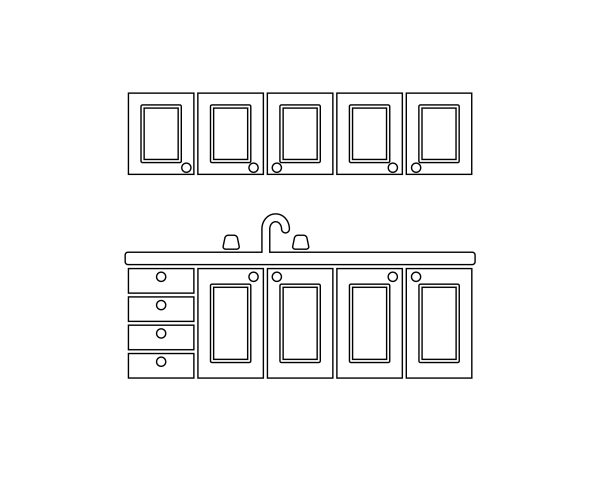


[English]: Please recall the decorations or utensils present in your kitchen.


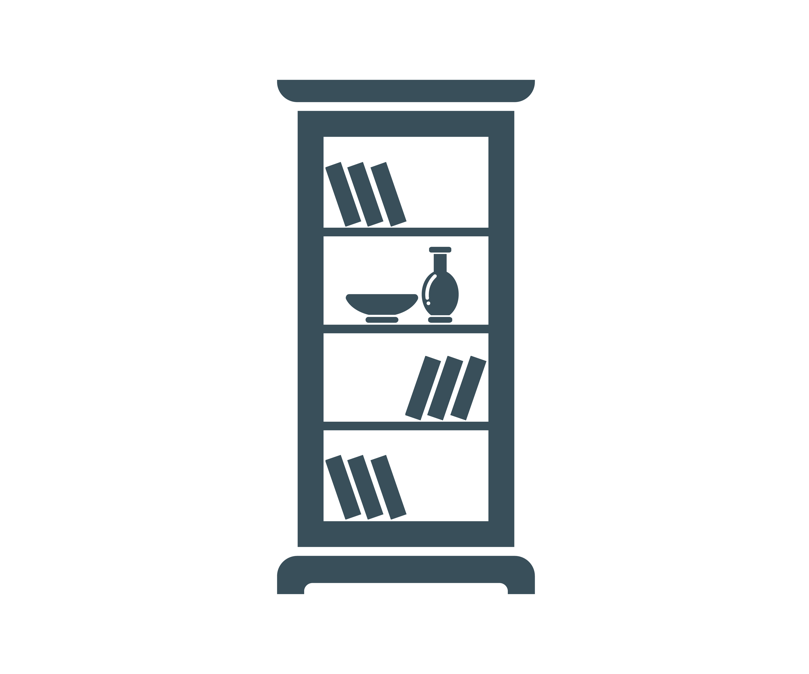


[English]: Please recall the types of plates and bowls that are placed in your kitchen?


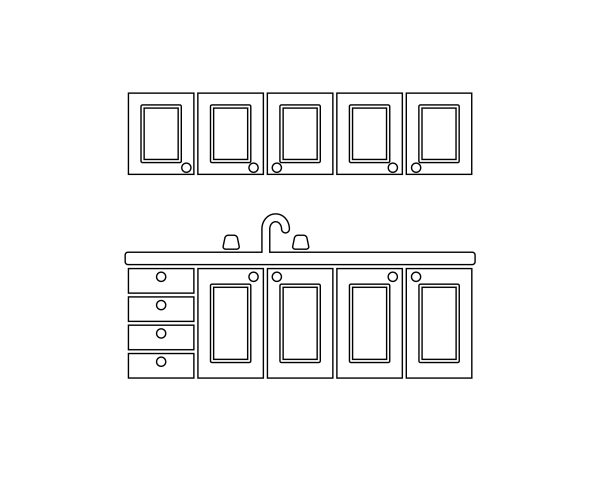


[English]: Now please close your eyes and recall all the details of the kitchen in your house. Focus specifically on memorizing the kitchen itself.

[Pause for 5s before the next page bottom appear]


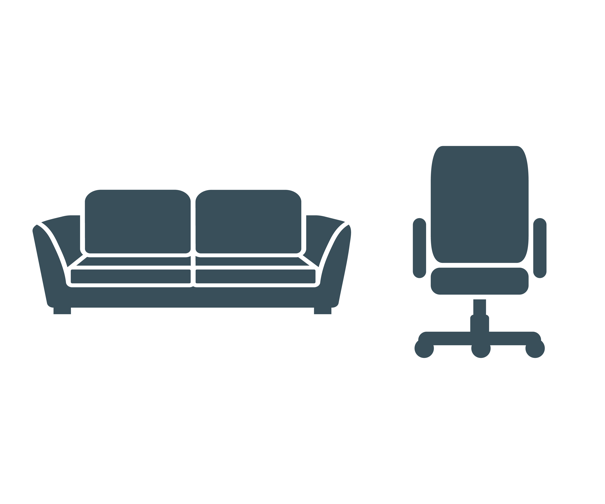


[English]: Please recall a chair in your house. Where is this chair placed in your home?


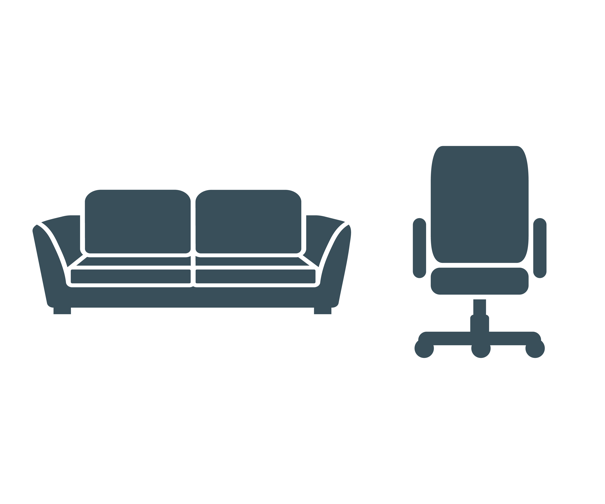


[English]: Please recall the material, color, and style of this chair.


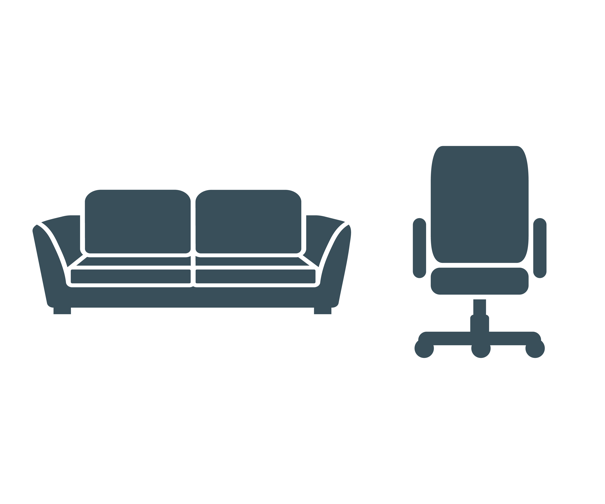


[English]: Now please close your eyes and recall all the details of the chair. Focus specifically on memorizing the chair itself.

[Pause for 5s before the next page bottom appear]

# Supplementary Table

## Table S1. Comparison of participants demographics between the two samples using different recruitment methods

| Characteristics | Random sample (N = 564) | Convenient sample  (N = 83) | Difference *p* ^a^ |
| --- | --- | --- | --- |
| **Sex (Female)** | 483 (86.3%) | 71 (85.5%) | 0.86 |
| **Age groups (yr)** |  |  |  |
| 18-34 | 145 (25.9%) | 22 (2.8%) | 0.74 |
| 35-44 | 342 (61.1%) | 47 (57.3%) |  |
| Above 45 | 73 (13.0%) | 13 (15.9%) |  |
| **Educational attainment** |  |  |  |
| Secondary or below | 281 (50.1%) | 45 (54.2%) | 0.48 |
| Tertiary or above | 280 (49.9%) | 38 (45.8%) |  |
| **Household monthly income ^b^** |  |  |  |
| <30,000 | 235 (45.5%) | 39 (52.7%) | 0.38 |
| 30,000 – 59,999 | 174 (33.7%) | 24 (32.4%) |  |
| >60,000 | 108 (20.9%) | 11 (14.9%) |  |
| **Employment** |  |  |  |
| Housewife | 227 (40.4%) | 33 (39.8%) | 0.27 |
| Part-time employed | 49 (8.7%) | 4 (4.8%) |  |
| Full-time employed | 275 (48.9%) | 42 (50.6%) |  |
| Unemployed/ seeking a job | 11 (2.0%) | 4 (4.8%) |  |
| **Accepting COVID-19 vaccines for children ^c^** | 427 (75.7%) | 53 (63.9%) | **0.02** |
| **Psychological distress level ^d^** |  |  |  |
| Normal distress level | 313 (55.5%) | 44 (53.0%) | 0.67 |
| Mild to severe distress level | 251 (44.5%) | 39 (47.0%) |  |
| **Affective response to the pandemic-related news at baseline** |  |  |  |
| News valence evaluation rating (SD) ^e^ | 1.31 (3.52) | 1.70 (3.51) | 0.35 |
| Affective state rating after reading the news (SD) ^e^ | 1.21 (3.41) | 1.48 (3.45) | 0.28 |

^a^ Difference between parents from the two samples. Significant difference was assessed using χ2 test for categorical variables and independent samples t-tests for continuous variables.

^b^ 1 HKD = 0.13 USD

^c^ Participants who accepted COVID-19 vaccines were those whose child had already received a COVID-19 vaccine or those who reported that they would ‘certainly’, ‘very likely’ or ‘likely’ to take their child for COVID-19 vaccination in the next three months.

^d^ Proportion of participants with mild to severe psychological distress was calculated based on the cut-off ≥3 for the PHQ-4 sum scores.

^e^ Sum score was calculated by aggregating participant’s ratings on the affective response of the three pandemic-related news items, with range from -9 to 9.

## Table S2. Key Measurement in the surveys

| Study measures | Items | Response |
| --- | --- | --- |
| **Baseline assessment at T0** | | |
| Psychological distress | PS1. In the past two weeks, how frequent do you feel not being able to stop or control worrying? | 0 = Not at all  1 = Several days  2 = More than half the days  3 = Nearly every day |
|  | PS2. In the past two weeks, how frequent do you feel down, depressed, or hopeless? | Same as above |
|  | PS3. In the past two weeks, how frequent do you feel little interest or pleasure in doing things? | Same as above |
|  | PS4. In the past two weeks, how frequent do you feel nervous, anxious, or on edge? | Same as above |
| Affective response to pandemic related news ^a^ | How positive or negative do you think this news is? (You can drag the slider below) | -3 to 3  -3 = Very negative  0 = Neutral  3 = Very positive |
|  | How do you feel after reading this news? | -3 to 3  -3 = Very pessimistic  0 = Neutral  3 = Very optimistic |
| Vaccination uptake ^b^ | Has your child received at least one dose of the COVID-19 vaccine? | 0 = No (Go to next question)  1 = Yes |
| Vaccination intention (uptake = 0) ^b^ | How likely will you take your child for COVID-19 vaccination in the next three months? | 1 = Definitely won’t  2 = Very unlikely  3 = Unlikely  4 = Even  5 = Likely  6 = Very likely  7 = Definitely will |
| Vaccine-hesitant attitudes | VH1. I trust the information I receive about COVID-19 shots | 1 = Strongly disagree  2 = Disagree  3 = Evens  4 = Agree  5 = Strongly agree |
|  | VH2. I prefer my child to develop immunity from natural methods rather than from COVID-19 vaccination | Same as above |
|  | VH3. I worry that too many COVID-19 vaccinations for my child may weaken his/her immunity | Same as above |
|  | VH4. I worry about the safety of COVID-19 vaccine for my child | Same as above |
|  | VH5. Overall, how hesitant about childhood COVID-19 shots would you consider yourself to be | 1 = Not at all hesitant  2 = Slightly hesitant  3 = Unsure  4 = Quite hesitant  5 = Very hesitant |
| Demographics | What is your gender? | 1 = Male  2 = Female |
|  | What is your age group? | 1 = 18-34  2 = 35-44  3 = 45 or above |
|  | What is your educational attainment level? | 1 = Secondary or below  2 = Tertiary or above |
|  | What is your occupation? | 1 = Housewife  2 = Part-time employed  3 = Full-time employed  4 = Unemployed / seeking a job |
|  | What is your household monthly income? | 1 = 30,000 or below  2 = 30,000-59,999  3 = 60,000 or above |
| **Intervention and immediate outcome assessment at T1** | | |
| Manipulation check | MN1. How positive or negative do you think the scenes you just imagined / recalled are? (You can drag the slider below) | -3 to 3  -3 = Very negative  0 = Neutral  3 = Very positive |
|  | MN2. How do you feel after completing the imagination / recall tasks? | 1 = Very bad  2 = Slightly bad  3 = No change  4 = Slightly good  5 = Very good |
|  | MN3. Overall, how vivid do you feel about the scenes you just imagined / recalled are? | 1 = No mental image at all  2 = Have some images but mostly are blur  3 = Have relatively vivid mental images  4 = Have very vivid mental images  5 = Have extremely vivid mental images |
| Affective response to pandemic related news ^a^ | Same as assessment at T0 except for using a different set of news | -3 to 3 |
| **Post-intervention assessment at T2** | | |
| Vaccine-hesitant attitudes | Same as assessment at T0 | 1 to 5 |

^a^ The valence and affect ratings were asked after each of the news item.

^b^ Those who reported that their child had received at least one dose of COVID-19 vaccine and those who reported being “likely/ very likely/ definitely will” to have their children vaccinated against COVID-19 were combined as having high vaccination acceptance.

## Table S3. Standardized model results of associations between variables in the Structural Equation Model

| Association | B | SE | P-value | 95%CI |
| --- | --- | --- | --- | --- |
| From PS to AR | -0.25 | 0.05 | < 0.001 | -0.35 to -0.15 |
| From AR to VA | 0.27 | 0.05 | < 0.001 | 0.16 to 0.38 |
| From PS to VA | 0.06 | 0.06 | 0.34 | -0.06 to 0.17 |

PS, psychological distress level.

AR, affective response to pandemic-related news.

VA, COVID-19 vaccination acceptance.

Beta coefficients, standard errors and 95% CIs for indirect effects were computed by the bootstrapping approach.

## Table S4. Two-way ANCOVAs for affective response to pandemic-related news to assess potential modification effect with psychological distress

| Key Variables | Valence evaluation rating  *F* (*P*-value) | Affective state rating  *F* (*P*-value) |
| --- | --- | --- |
| Condition | **8.35 (0.004)** | **6.13 (0.014)** |
| Psychological distress level (PS) ^a^ | **55.84 (<0.001)** | **55.58 (<0.001)** |
| Condition × PS | 0.41 (0.52) | 1.66 (0.20) |
| Baseline affective response rating ^b^ | **133.33** **(<0.001)** | **171.09 (<0.001)** |

Bold font indicates significant *p*-value.

^a^ Psychological distress was input as a continuous variable derived from the PHQ-4 scale

^b^ For the dependent variable of valence evaluation rating, baseline valence rating was input in the ANCOVA model; for the dependent variable of affective state rating, baseline affective rating was input in ANCOVA model.

## Table S5. Comparison of participants demographics across the three time points

| Characteristics | T0 sample  (n=647) | T1 sample  (n=632) | T2 sample  (n=328) | Difference  *p* (T1 vs. T2) ^a^ |
| --- | --- | --- | --- | --- |
| **Sex (Female)** | 554 (86.2%) | 542 (86.3%) | 279 (86.1%) | 0.97 |
| **Age groups (yr)** |  |  |  |  |
| 18-34 | 167 (26.0%) | 162 (25.8%) | 79 (24.4%) | 0.59 |
| 35-44 | 389 (60.6%) | 382 (60.9%) | 199 (61.4%) |  |
| Above 45 | 86 (13.4%) | 83 (13.2%) | 46 (14.2%) |  |
| **Educational attainment** |  |  |  |  |
| Secondary or below | 326 (50.6%) | 317 (50.4%) | 165 (50.8%) | 0.94 |
| Tertiary or above | 318 (49.4%) | 312 (49.6%) | 160 (49.2%) |  |
| **Household monthly income ^b^** |  |  |  |  |
| <30,000 | 274 (46.4%) | 264 (45.8%) | 150 (48.9%) | 0.36 |
| 30,000 – 59,999 | 198 (33.5%) | 195 (33.9%) | 101 (32.9%) |  |
| >60,000 | 119 (20.1%) | 117 (20.3%) | 56 (18.2%) |  |
| **Employment** |  |  |  |  |
| Housewife | 260 (40.3%) | 252 (40.0%) | 136 (41.7%) | 0.29 |
| Part-time employed | 53 (8.2%) | 53 (8.4%) | 27 (8.2%) |  |
| Full-time employed | 317 (49.1%) | 310 (49.2%) | 159 (48.5%) |  |
| Unemployed/ seeking a job | 15 (2.3%) | 15 (2.4%) | 4 (1.2%) |  |
| **Vaccination acceptance ^c^** | 480 (74.2%) | 467 (73.9%) | 252 (76.8%) | 0.12 |
| **Psychological distress,** Mean (SD), range ^c^ | 2.94 (2.84)  0-12 | 2.52 (2.48)  0-12 | 2.27 (2.47)  0-12 | 0.29 |
| **Affective reactions to news at baseline** |  |  |  |  |
| News valence evaluation, Mean (SD), range | 1.36 (3.51)  -9-9 | 1.39 (3.52)  -9-9 | 1.28 (3.61)  -9-9 | 0.56 |
| News affective rating, Mean (SD), range | 1.24 (3.42)  -9-9 | 1.26 (3.42)  -9-9 | 1.10 (3.51)  -9-9 | 0.29 |

^a^ Difference between parents who completed T2 assessment and those who did not. Significant difference was assessed by χ2 test for categorical variables and independent samples t-tests for continuous variables.

^b^ 1 HKD = 0.13 USD

^c^ Vaccination acceptance included those who already received a COVID-19 vaccine for their children or reported to have a ‘Certain’, ‘Very likely’ or ‘Likely’ vaccination intention in the following three months.

# Supplementary Figure

Figure S1. Number of confirmed cases during study period


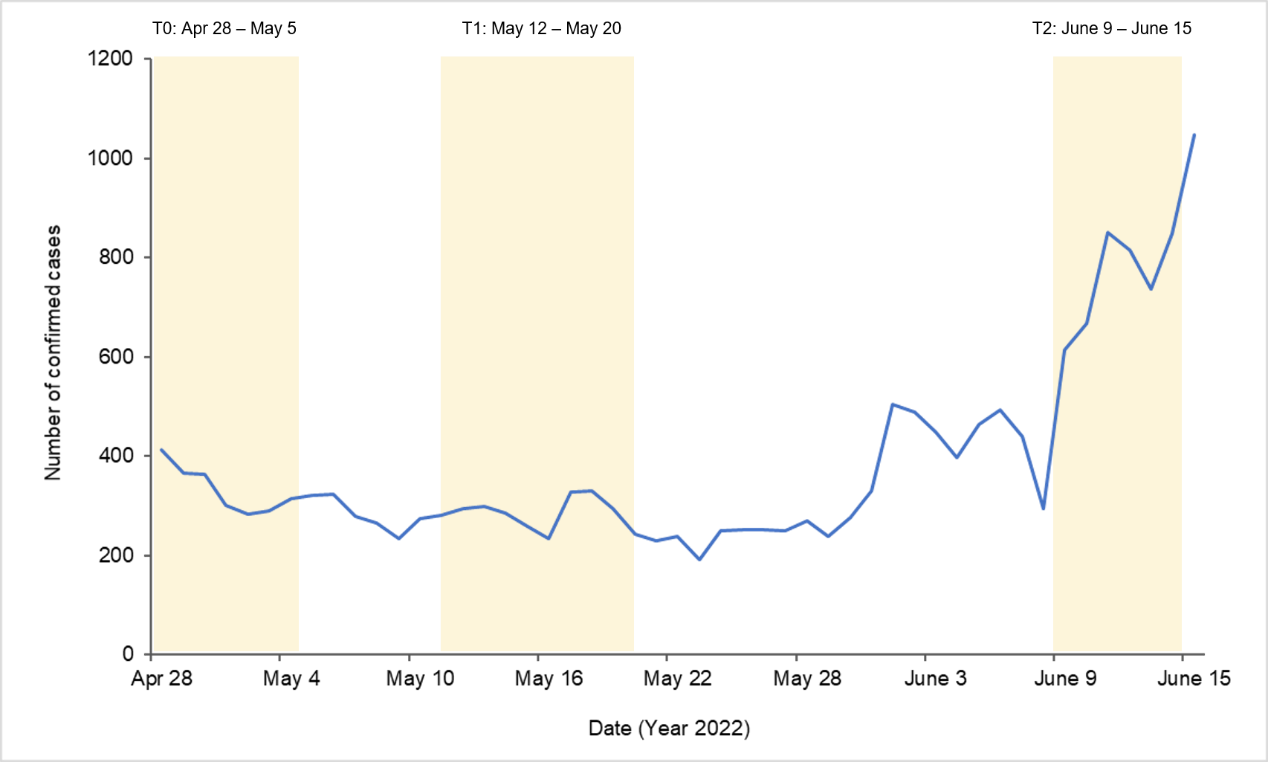


*Shaded yellow indicates data collection period. The confirmed cases were obtained from the Real-time dashboard developed by the University of Hong Kong, School of Public Health:* [*https://covid19.sph.hku.hk/*](https://covid19.sph.hku.hk/)

## Figure S2. Childhood COVID-19 vaccination uptake rates by the study periods and doses in Hong Kong


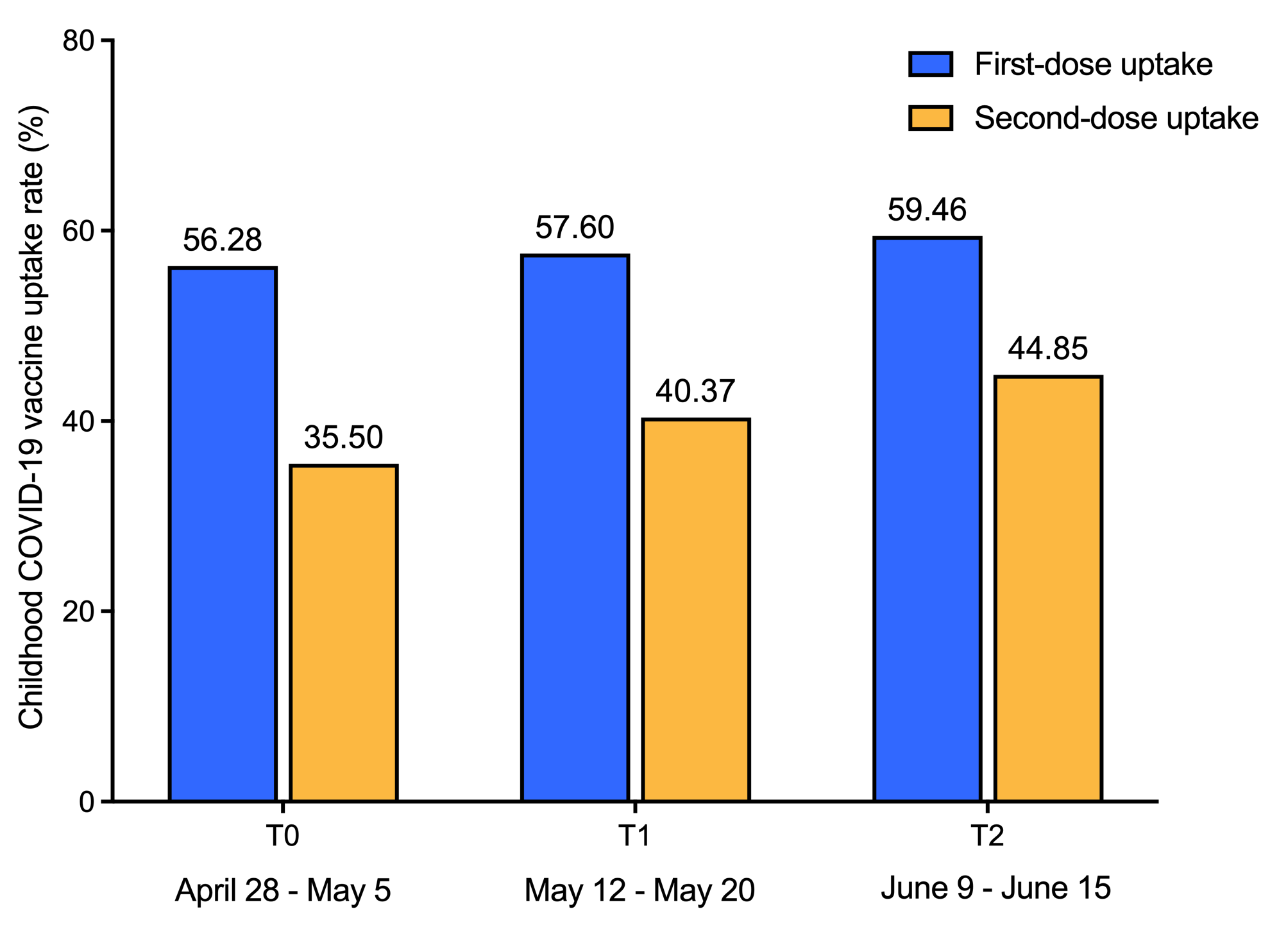


*Figure shows the raw percentage of childhood COVID-19 vaccination uptake in 2022. The blue color represents the percentage of first-dose COVID-19 vaccine uptake by the survey dates; while the orange color represents the percentage of second-dose COVID-19 vaccine uptake by the survey dates. Vaccination uptake data were obtained from the publicly available government webpage:* [*https://www.healthbureau.gov.hk/download/opendata/COVID19/vaccination-rates-over-time-by-age.csv*](https://www.healthbureau.gov.hk/download/opendata/COVID19/vaccination-rates-over-time-by-age.csv)*. The total number of children aged 0 -11 was sourced from the 2021 census data:* [*https://www.censtatd.gov.hk/en/EIndexbySubject.html?scode=600&pcode=B1120117*](https://www.censtatd.gov.hk/en/EIndexbySubject.html?scode=600&pcode=B1120117)
